# Supplementary material for: Genomic insights into CKX genes: key players in cotton fibre development and abiotic stress responses
Source: PeerJ. 2024 May 30;12:e17462. doi: 10.7717/peerj.17462 (PMC11144395; doi:10.7717/peerj.17462)
Supplement: Supplemental Information 9 — The sequences are available at the Cotton Functional Genomics Database, https://cottonfgd.net. [file peerj-12-17462-s009.docx]

**Supplementary File 1**. Sequences of all identified CKX genes in four cotton species.

>GaCKX02

MKSIYYSLPFLFVVVCSLSWVNASANSHENFLDCLSSYHPQESSSISKVIYTETNSSYSEILESSIQNYRFFTTNTPKPLVIVTPLNISHVQATIHCSKKHGLQIRIRSGGHDFEGLSYVSQVPFVVIDLVHFRSIDVDVENEEAWIQSGAITGEVYYRINERSTNLTFPGAVGHTVGIGGFISGGGYGLLFRKYGLAADNVIDAMFVDANGRVLDRKSMGEDLFWAIRGGGGGSFGIVLSWKVKLVHVPSTVTVAAVRRTLEQNATQLLHRWQYVAPNLPNDVYSVVSISTTNSTENGERTVVATFVSVFQGGANEFIPLMQERFPELGLVREDYIEMTWIESILLLTGLTNQTSEVLLDRSYKNFFLSPSFKGKSDYMRKPMPEIVIQGLWSRLLEDEARISTLNIIAYGGKMDEIPETEIPFPHRKGTLYKISYYVGWQEEDNSNPQRYISWIRKVYKYMGPFVSKYPREAYLNYRDLDIGRNNNEGKASYKQASVWGRKYFKENFDRLTYVKAKIDPENFFRHEQSIPPRFH

>GaCKX03

MKSLHGCLLLLFVVVFSLSWVNASANSNDYFLDCLSSYHPDESTSFSKVIYTETNSAYSAVLESSIRNPRFSTPNTPKPLVMVTPLNISHVQATIHCSKKHGIQIRTRSGGHDFEGLSYVSQVPFVVIDLVHFRSIDVDVKKEEAWIQSGAITGEVYYRINERTTNLTFPGALCHTVGIGGFISGGGYGFLFRKYGLAADNVIDAQFVDANGRVLDRRLMGEDLFWAIRGGGGASFGIVLSWKVKLVHVPSTVTVFSVGRTLEQNATQLLHRWQYVAPNLPNDVYSIVSISSMNSTENGERTVLATFTSVFQGVADELIPLMQERFPELGLLKEDYIEMTWIESILFWNQLSNETSEILLDRSNSNSLMPLSYKSKSDYVRKPMPEIALQGLWSRLLEVDETSTAVVNIISYGGKMDEIPETETPFPHRKGTLYKINYNIVWQEEENSNPQRYISWMRTLYSYMGPFVSRSPRAAYVNYRDLDIGWNNDDGKTSYKQGSVWGRKYFKNNFDRLVYVKTKIDPENFFKHEQSIPPRFH

>GaCKX04

MKSLYRSLPLLFVVVCSLSWVNVSANSHDDFLDCLSSYHPDESSSISKVTYTETNSSYSAVLESSIHNHRFSTPNTPKPLVIVTPLNISHVQATIHCSKKHGLQIRTRSGGHDYEGLSYVSHVPFVVIDLVRFKSVEVDVENEEVWVQSGAIVGEVYYRINERSTNLTFPGAFGYTVGIGGFISGGGDGLLSRKYGLAADNVIDVQFVDASGRVLDRRSMGEDLFWAIRGGGGGSFGIVLSWKIKLVHVPSIVTVFSVGRTLEQNASQLLHRWQYVAPNLPNDVYSLLTISKWNAGENETRTVLVTFTSLFQGGANELIPLMQERFPELGLVKEDFVEMTWLESVLFINGLSNETSEVLLDRYRPLLPPSFKSKSDFVNEPVPEIALQGLWPQLLEVDEAISAVQTFIAYGGMMDEISETETPFPHRKGTLYKIHYNIGWQEEENIRSQRYISWMRKLYSYMGPFVSKSPRTAYVNYRDLDIGRNNDDGKASYKQASVWGRKYFKNNFDRLVSVKTKIDPENFFKHEQSIPPHFH

>GaCKX05

MKSLNRSLSLLFIVVCSLLWVSASANCHDDFLECLYSYHPKESSSITQVIYTETNSSYSAVLDSSIRNHRFSTPNTPKPLVIVTPLNISHVQATIHCSKKHGLQIRTRSGGHDYEGLSYVSHVPFVVIDLVNLRSVDVDVENEEAWVQSGATVGEVYYRINERSTNLTFPAAVGRTVGIGGSISGGGDGLLFRKYGLSADNVIDALLVDANGRVLDRRSMGEDLFWAIRGGGGGSFGIVISWKIKLVHVPSTVTVFSVGRTLEQNATQLLHRWQYVAPNLPNDVYSVVTISTTNASENGTKTVLATFISFFRGGANEFIPLMQERFPELGLVKEDFIEMTWIESLLLMNGASNETSEILLDRSNRYSLLPPSFKSKSDYVREPMPEIALQGLWLHLLEIDEGGIAVQNFFAYGGIMEEISETETPFPHRKGTLYKISYNIGWQEEENNNSQRYISWIRKLYSYMGPFVSKSPREAYVNYRDLDIGRNKYYGKTSYKQASIWGRKYFKNNFDRLVYVKTKTDPKNFFKHEQSIPPRFH

>GaCKX06

MESLNRSLSLLFIFVCSLSWVSASANSHDDFLECLYSYHPKESSSITQVIYTETNSSYSAVLDSSIRNHRFSTPNTPKPLVIVTPLNISHVQATIHCSKKHGLQIRTRSGGHDFEGLSYVSHVPFVVIDLVNLRSVDVDVENEEAWVQSGATVGEVFYRINERSTNLTFAAAVVRTVGIGGLISGGGDGLLFRKYGLSVDNVIDAQLVDANGRVLDRRSMGEDLFWAIRGGGGGSFGIVIAWKIKLVHVPSTVTVFSVGRTLEQNATQLLHRWQYVAPNLPNDVYSLVAISTTNASENGAKTVLATFTSLFQGDANEFIPLMQERFPELGLVKEDFIEMTWIESLLLMNGVSNETSEILLDRSNRYSLLPPSFKSKSDYVREPMPEIALQGLWPQLLEVDEGGIAVQNFIAYGGIMEEISETETPFPHRKGTLYKINYNIGWLEEENNNSQRYISWMRKLYSYMGPFVSKSPREAYVNYRDLDIGSNNYYGKTSYKQASIWGRKYFKNNFDRLVYVKTKTDPKNFFKHEQSIPPRFH

>GaCKX07

MAFWRISICSLLLLLLSSSTMATASISRPRKRPTSSLIQEKFIQCFTGSSQFYIPLSTAFFTPNNASFTSVLQSTSQNLRYLVPSMPKPELIITPLHESQAQASVICAKRLDIHLRVRSGGHDYEGLSYVSQIESPFVIVDLSKLRLIKVDIQDNSAWVEAGATIGEVYYRIAEKSNIHGFPGGLCTSLGIGGHITGGAYGSMMRKFGLGADNVIDARIVDVNGRVLDRAAMGEDLFWAIRGGGGASFGIILEWKIKLVPIPATVTVFTVTKSLEQGATKLLYKWQTVADKLDEDLFIRVIIQTANAGKNNAKTVTTSYNALFLGDAERLLRVMQQSFPELGLTRKDCTETSWIKSVLYIAGYSSNTPAEILLQGRSTFKNYFKAKSDFVKEAIPETALEGLWKRLLEEDSPLMIWNPYGGMMARISESQIPFPHRQGIKFKIQYLTLWQVEDNNASKHFDWIRRLYNYMAPYVSMFPRGAYVNYRDLDLGMNKNINTSFIEASLWGVRYFKDNFMRLVKVKTRVDPNNFFRHEQSIPPLPVQARY

>GaCKX08

MKDSNSATLPLVTIVLFLSLSWRATSDSSSQVDKFLQCLANSSALMLESIYTPSNSSFESALQAYIRNHRFLTPETPKSVAIVAPTHVSHVQATVVCAKDNGVQIRVRSGGHDYEGLSYRSNVTFVILDMFNLRSIDVDVDNEVAFVQAGATIGELYYKIANQSKDHAYPAGVCLSLGTGGHFNGGGYGNMMRKYGLSVDNILDAQVVGADGNILDQASMGKDLFWAIRGGGGASFAVIVS

>GaCKX09

MAIHCSQNMKFLQFSVLPFLMVILSVSGANLTHHPHRDFLRCLSLRIENSCTITYTHNNPSYPSVLNASIQNTRFSTPTTPKPYAIITPRKTSDVQSTIFCSKNYGFQLRIRSGGHDVEGVSYVSQVPFVVLDLVNFRDVKVDTKNEVAWVQSGATTGELYYGIAAKTQTLGFPAGICHTIGIGGHLSGGGFGILGRKYGLAADHIIDAKMVDANGRVLHRKSMGEDLFWAIRGGGGNTFGVVLAWKIKLVPVPPVVTVFTVNKNLEQNATKIFHRWQQIAHKLPNDLFTTVWVMKVNSSQVGKKTVQASFKGLFLGRIDELIPLIQYAFPELGLARENCTQMSWVQSVLYFGALPIEPVEILLNRSALPRLSLKAKTDYIREPMSEAGFEGFMNMFLEEGTDLAITMMEAFGGKMNEIRENEIPFPHRSGILFESVYIVQWINEEDAGLCINWMRRLYNYMSSYASKSLREAYYNYKDLDLGINNVNGYTSYEQASVWGLKYFKNNFKRLVRVKTMIDPTNFFSNEQSIPPLLSP

>GaCKX10

MHFLRNLIILFLICIAIKINLCVPTIPSSLKTLPIDGHFDFKQVHHAAKDFGNRYSFLPSAVLHPKSASDIATTVKHIWEMGPGSHLTVAARGHGHSLQGQAQAHRGVVINMESLQGPKMKVHTGNFPHVDVSGSELWINILHETLKHGLAPKSWTDYLHLTVGGTLSNAGISGQAFRHGPQISNVRQLEVVTGKGEVVNCSEKQNSDLFYSVLGGLGQFGIITRARISLEPAPKMVKWIRVLYTDFATFAKDQEMLISGGSTFDYIEGFVIINRTGLLNNWRSSFNSNDSAQASHFKSDGRTLFCLELAKYFNPEEMAIVNQEIMTSLSQLNHIPSTLFQSQVSYIEFLDRVHISEIKLRSKGLWEVPHPWLNLLIPRSNIHIFAQLVFGNILTNTSNGPILIYPVNKSKWDNRTSVVLPEEDVFYLVAFLSSAAPSSTGSDGLEHILSQNKRILELCEIDGLGVKQYLPHYSTNGEWRSHFGPQWEAFVHRKSTYDPLAILAPGQRIFQKAAPLSL

>GaCKX11

MIAYLGRIVHDNDAESKLDDDVSSISKSLDLQGSIENGDVSSLASKDFGGLYSVKPLFLIKPSGAEDISRVVKLASRTSNLTVAARGNGHSINGQAMAEGGLVIDMRSTEKNHFEFLPINGSHYIDVSGGALWEDVLTRCVSRYGYAPRSWTDYLSLTVGGTLSNAGVSGQAFRYGPQTSNVTELEVVTGKGEITVCSETLNSELFFGVLGGLGQFGIITRARIKLQQAPDMVRWIRVVYSEFEEFTRDAEFLVTQKEGESFDYVEGFVFCNNDDPFNGWPSVPLDPGHEFNPTHISQTAGSILYCLEVAFHYRNSDHPTVDTAVNGLLGRLRFVEGLKSQVDVSYTKFLLRVNRAEEQVKANGPGDGPHPWLNLFVSKSDVVNFDRTVFKTMLKDGVGGPMLIYPLLRSKWGDRTSVVLPEGEIFYIVALLRFVPNGPSVEKLVAQNREIVNWCIKVGLDFKLYLPHYQSKGDWERHFGNRWSRFVERKASFDPMAILAPGQNIFRRDPSNIIISREF

>GaCKX12

MRGLPILVSIILFSISLVTSKSYPNSVVDNFLQCLPKHYSNPSKPIAKAIYTPTHPSFQSVYELHTYNLRVLTQANATHKPMAIVAALHESHVQAAVICAKESGLQVRTRSGGHDYEGLSFSSSVPFVIIDLSNLRSIKIDMKTQTAWVQAGATTGELYYRIAEKSKVHAFPAGVCTTLGIGGHFTGGGYGNMLRKFGLSIDNVVDAQLIDAKGRILNRKSMGEDVFWAIRGGGGTSFGIILSWKIKLVRVPPKVTVFQVAKTLEQGATVLVHKWLQVSHKLDKDIFIRIMPVTVDGTGNGNSTVRVSFIGHYLGRINRLLPLVNASFPELGLQRKDCTEMSWIESTLYWAGFPIGTSTDVLLNRVPNKVFFKTKSDYLKNVMPKAGLETLWKVMMEIGNMWMQMSPYGGRMAEISESETAFAHRAGTLYIVQYTAHWSEGSSEATKKYVELMRKLYAEMAPYVSSKPREAFLNYRDLDIGSNNTDFEAAKVYGAKYFKGNFQRLAEVKAKIDPHNFFKNEQSIPPFPSF

>GaCKX13

MATKLLLTFAICRLIVTVGLTLDPTELLLLGVDAQLSVDPTDVKAASLDFGLLIGAQPPLAVMHPASSQDVAQFVKAAYGSNFGFTVSARGHGHSINGQAQTANGVVVQMSGSKGGSGMASGRKPPHPRVWPQERFVDVWGGELWIDVLRSTLQHGLAPKSWTDYLYLSVGGTLSNAGISGQAFNHGPQISNVHELDVVTGKGELLTCSEEQNSEMFHAVLGGLGQFGIITRARISLEPAPQRVRWIRVLYSNFSIFTSDQEYLISLHEQPASQKFDYVEGFVIVDEGLINNWRSSFFSPHNPVKISSLDPNGGVLYCLEIAKNYHESTASTVDQEVESLLKKLNFIPASVFTTDLPYVDFLDRVHKAELKLRSKGLWEVPHPWLNLFVPKSKIADFDKGVFKGILGNKTSGPILIYPINKNKWDHRSSAVTPDEDVFYLVALLRSALDNGEETHSLEYLTNQNRQILRFCDEAGITVKQYLPHYTTHQEWVDHFGNKWDRFYRLKMEFDPRHILASGQQIFTPTNMASWR

>GaCKX14

MATKLLLTFAICRLIVTVGLTLEPTELLRLGLTVDPVDVESASVDFGLMTKVEPLAVLRPSSAEDVAQLVKAAYESSHGFTVSARGHGHSINGQAQTGTGVVVQMSGVRSGGKPSVWGGDMYVDVWGGELWIDVLKSCLEYGLAPKSWTDYLYLSVGGTLSNAGISGQAFHHGPQISNVHELDVVTGKGELMTCSKEQNQELFIAVLGGLGQFGIITRARISLEPAPQRVRWIRVLYSNFSAFTKDQEYLISLHGNQKFDYVEGFVIVDEGLINNWRSSFFSPRNPVKISSLGSNNNGGVLYCLEITKNYHESTAETIDQEIEALLKKLNFIPTSVFTTDLPYVDFLDRVHKAELKLRSKGMWEVPHPWLNLFVPKSKIADFDRGVFKGILGNKTSGPILIYPMNKNKWDDKSSVVTPDEDVFYLVAFLRSALDNGEETQSLEYLTNQNRQILKFCDEDGIKVKQYLPHYKTQSEWKEHYGSKWDRFQRMKMKFDPRHILASGQNIFTPTFLSSSKMASW

>GaCKX15

MAVALPSFFTAIMIMSRLMAFIGISKNNDMSSKLQALDIAPKLSHDPSAIESASQDFGHIVKAAPQAVLLPSSPRDIASLVNFSYSNSVPFSIAARGNSHSVNGQAMAKNGVVIDMTSMKSGNGTGIRIASDGSYVDVGGQQLWIDVLNATLGLGLTPVSWTDYLYLTVGGTLSNAGISGQTFRYGPQISNVYEIDVITGTADFVTCSPNNNSDLFYAALGGLGQFGIITRARIPLEPAPKRVKWVRMLYTDFLDFTRDQELLISKNGRNNNKALNYLEGSLLLDQGSLDNWRSSFFPPQDQPKIISLITKFRIVYCLEIVKHYDGQTKTTVDKDLQQLLKGLSYLPGFMFEKDAKYEEFLNRVHSEELKLKAKGLWDVPHPWLNLFIPKSKISDFNDGVFKSIVLKRNITTGPVLVYPMNRKKWDDRMSAVIPDEEIFYTVGLLQSSGFDDWRTFEDQNKEILQFCEKAGIKVKQYLPHYTTKVGWVNHFGSKWSTFQKRKLQFDPKLLLSPGQRIFNNNQ

>GaCKX16

MIACLGRIVHDSDAESKLDDDVSTIFNSLNLQGSIENGDVSGIASKDFGGLYSVKPLYLIRPSGAEDISRVVKAAAGTPHLTVAARGNGHSINGQAMADGGYVIDMRSTGENHFKLLTVNGSPCIDVSGGALWEDVLRRCVSRFGLAPRSWTDYLSLTVGGTLSNAGVSGQAFRYGPQTSNVTELEVVTGKGDITVCSETQNPELFFGALGGLGQFGIITRARVKLQLAPDMVRWIRVVYAEFEEFTRDAEFLVSQKEDESFDYVEGFVFCNNDDPVNGWPSVQLNPDQEFNPAHLPQTAGPVLYCLELAFHYRNSDQPSTVDMAVSRLVGGLGFVDGLISQVDVSYMGFLLRVKRAEQDAKANGVWDNPHPWLNLFVSKSDIVDFDQTVWDSRTSVALPEGEIFYIVALLRFVPKGPSVEKKVAENREIVKWCIKEGLDFKLYLPHYRAKEDWKRHFGNQWTRFEKRKANFDPMAILAPGQRIFKRTNQ

>GaCKX17

MPNPMRPYLLLSLVFFFNLYHSMAVSDPTHQALLQCLTQSIPTDTASSIIVSKSNPSYTSVLRAYIRNARFNTSSTPKPLIIITPLDESHVSAAVICSQKLGFQLKIRSGGHDYEGLSYVFDKPFFVLDMFNLRSITVNMADETAWVGAGATLGELYYNIWKNSKVHGFPAGVCPTVGVGGHLSGAGYGTLIRKYGLSVDHVVDAKLVDVKGKILDRKAMGEDLFWAIRGGGAASFGVVLSYKIKLVPVPETVTVFRIERLLTENATDITFKWQTIAPTTDENLFMRMLLQPVTRNKKKTARITVIALYLGDSDSLVSLLQKDFPELSIGKSNCNETTWIDSVLWWANFDLGTPPTALLDRDLNDAGFLKRKSDYVQTPIPKSGLESLWQKMIELGKVGMVFNAYGGRMDQIKPDETPFPHRAGNLYKIQYSVNWDERGNEADKNFTTQAKLLHDFMTPFVSKNPRSAYFNYRDIDVGSTKKWSYEEGKVYGESYFNGNYERLVDVKTAVDPNNFFRNEQSIPPRSSFILHSSHGSATYLITIIHTCWYYIVFIFSLCSDLF

>GaCKX18

MAISWPLVVSVLLSISSLVTSASNSDSVHEAFVQCLLDNSHPSHPISEAIFTPQSPSYATVLQSYIRNLRFNETYTPKPFLILTALHQSHIQAAIICAKKGNIQMKIRSGGHDYDGLSYVATVPFFVLDMFNLRSIDIDTETETVWVQSGAILGELYYRISELSKTHGFPAGVCPTVGVGGHFTGGGYGNMMRKYGLTVDNIVDAYFIDVNGRIHDRKSMGEDLFWAIRGGGAASFGVVLAYKIKLVHVPEIVTVFRVEKTLEDNATDIVDQWQHVASKLPKELFVRLVIDVVNSSTRTGGSTVRVSFISLFLGDSKTLVSIMNENLPLLSLSQSDCIETSWIRSVLFWTNIAIDSPTDVLLNRTPSLSYLKRKSDYVKQPIPKTALEGIWEKMIELQPAQMIFNPYGGRMAEIESTATPFPHRAGNLWKIQYLANWNQGGAETAQRYIGLTRKLHRYMTPFVSKNPREAFLNYRDIDLGVNHNDRGSYLEGRVYGIKYFKGNFNRLVHIKTKVDPTNFFRNEQSIPTLPH

>GaCKX19

MNSSSSKLFILSTSFLLSISSVTSNSVLDNFLQCLPIHSNTSNPITNSIYVPNNSSFQYLYELRANNLRIISSSTISKPLAIITARHASHVQAAVICAKIHSIQLRIRSGGHDYEGLSYKSDIPFVILDLFNLRSTKINITSETAWVQAGATTGELYYKIAEKSKVHGFPSGVCTTLGIGGHFTGEDVFWAIRGGGGTSFGVILSWKIKLVHVLRKVTVFKVQRTLDQGATDIAYSWQHIAPKLPKHLFIRLQPEPITIGNGNKTIRVSLIGQFLGRSRKLMNLMNEYFPELGLQQNDCIEMSWVESTLFWAGFTNGTSIDVLLNRVVENKVFFKTKSDYYKNVIPKQGLVSLWEMLMDIGNIFVQLNPHGGRMDEISETETAVHRRCGYLFKVQYTVYWSESDGGIGAAKRYVEMSRRLYGAMAQYASSDPREAFLNYRDLDIGCNGSNDTDFGVAEVYGTKYFNNNIMRLASVKAILDPESFFKNEESIPPLPSSH

>GaCKX20

MAVSFPIPSYFTAIFIISRLMSIMGISKHLNNKLLPPLDNITDKLSLDPSAIESASQDFGHIVKSIPKAVLQPSSIADIASLINFSYNSSIPFTIAAKGHGHSVRGQAMANDGVIVDMTSMKKHRNGTGIWVSNDGVYADVGGEQLWIDVLNATLKHGAAPVSWTDYLYLTVGGTLSNGGISGQSFRYGPQISNVYEMDVITGKAEIVTCSPNKNSELFYAALGGLGQFGIITRARIPLEPAPKRVKWIRMLYNDFTAFTRDQELLISINGRHDSHALGYLEGSLLMDHGSPDNWRSSFFPPKHHPKITSSIINHRIIYCLEVVKHYDYQTQNTVDKELEQLLKGLSYMPGFMFEKDVLYAEFLNRVQRGELKARSQGLWDVPHPWLNLFIPKSQIQRFNDGVFKGIVLEGNITTGPVLVYPMNRKKWDDRMSAVIPDEEIFYTVGFLHSSGFDDREAFDDQNKEILKFCEEAGIGVKQYLPHFTSKDEWVHHFGSKWETFQQRKFQFDPKMILSPGQRIFNNN

>GaCKX21

MIQKMPPVVGSNLHKTIQYENTSNFWLFEPGALGPSGFLEESNGRLVHCYQAKAFTITPGTQLNPTSSYCCSYHNKKENRSPPKQTLFEDPVQCNRTICTLSNSYGAWGDRKDCSVKSVVYPTTEEELRSAVAHANKNKLKVKVVSKFSHTIPKLACPSSLGHHDSLLISTAKYDSSIEIDSVNLEVTADAGVALRDVIDKVEEAGLSLAAAPYWEGVSVAGMISTGAHGSSCMIVPGNESEGYAKVKQIGAQDQLLNAAKVSLGILGVISKVKLSLQRGFKRSITYNFTNDSSIENNFMEHGKKYEFGDITWYPSKHTAGYRYDSRVPMETPGDGINDFLGFQSNEILISKSVRASEKLFESTKSVNGECTLADTTLWYKKQIGNGLKNNGQIFTGYPVVGRQGKMQTSGSCLYSPKTRIDASCAWDPRIKGLFFYESTAIFTATKFGDFIKDVKKLRDLKPENFCGIDHYNGFLIRYIKASKAYLGQSEDSIVVDFNYYRADEASTPRLNQDVMEEVEQMAFFKYGARPHWAKNRNLAFLKVQSKYLNFNKFIAVKKQLDPENMLSSEWSDEILFGKEGVGSDGCALEGLCICSEDRHCSPSQRYFCKPGLVYSEARVCRHSPSSSNL

>GaCKX22

MVNLSLLLLHFLISSLSVSGSAATDQRNIMSCLNYYNISNYTISSNVHNHDYSILLNFSIQNLRFAEPTIPKPIAIILPENKEQLINTVVCCTIGPWEIRVRCGGHSYEGTSSVASDGAPFVIIDMMNLNRVSVDLGNETAWVEGGATLGETYHAIAESSDTHGFAAGSCPTVGTGGHIGGGGFGFLSRKYGLAADNVIDALLLNAEGELLDRQAMGEDVFWAIRGGGGGIWGIVYAWKIKLLKVPKTVTSFIVSRPGTIAHVANLVNKWQHVAPNLEGDMYLSCAVGAGLPQAKSIGISATFNGFFLGRKREAVLILGQVFAELDVSEEACKEMSWIESVLFFSGLGDGAIVSDLKNRYLHDKHYFKAKSDYVRNPISLTGIRTAIDILEKQPKGYIIMDPYGGIMNNISNDSIAFPHRYGNLYTIQYLVEWHQEEKNRSNEYREWIRDFYDAMASHVSWGPRAAYVNYMDFDLGVMELINTSVLSEDTVEMARVWGEKYFLNNYDRLVKAKTLIDPNNVFKNQQGIPPSTTTGLKARAF

>GaCKX23

MVNLSLLLFLISSPSVSGSASPDQRNIMSCLNSYNISNYTISSNVHNHDYSILLNFSIQNLRFAEPTIPKPIAIILPENKEQLINTVVCCTKGPWEIRVRCGGHSYEGTSSVASDGALFVIIDMMNLNRVSVDLGNETDGWKVVQHLVRHTMPLPIQVAFMDSQQDHVLRKYGLAADNVIDALLLNAKGELLDRQAMGEDVFWAIRGGGGGIWGIVYAWKIKLLRVPKTVTSFIVSRPSTKAHVANLVNKWQHVAPNLEGDMYLPCAVGAGLPQAKSIGISATFNGFFLGRKRGAVLILGRVFAELGVAEEDCKEMSWIESVLFFSGLGDGAIVSDLKNRYGNLNTIQYLVEWHQEEKNRSNEYREWIRDFYDAMASHVSWGPRAAYVNYMDFDLGVMELINTSVLSEDTVEMARVWGEKYFLNNYDRLVKAKTLIDPNNVFKNQQGIPPSTTTGLKARTF

>GaCKX24

MKFLQFSVLPFLIVILSLIGATLAHPYGDFLHCLSLRISNSSTISKVIYTQNNPSYSSVLNASIQNTRFSTPTTPKPYAIITPRKTSHVQSTIYCSKNHGFQFRIRSGGHDVEGVSYVSQVPFVILDLVNFRAVKVDAKNEVVWVQSGATTGELYYGIASKTQTLGFPAGICHTIGIGGHLSGGGFGILGRKYGLAADHIIDAKLIDAYGRILNRKFMGEDLFWAIRGGGGNTFGVVLAWKIKLVPVPPVVTVFTVNKNLEQNATKIFHRWQYIAHKLPNDLFTAVWIMKVNSSQVGKKTVQAGFRGMFLGGVDELIPLIQHEFPELGLAKENCTQMSWVQSILYFGGLPIQPVEILLNRNALPRSSLKAKTDFVKEPMPETGIEGFMNMFLEEEADFAITMIEAFGGKMDEIQENELPYPHRAGILFESTYIVQWTNEEDAGTYINWIRRLYSYMASYVSKSPREAYHNYKDLDLGTNNVNGYTSYEQASVWGLKYFKNNFKRLVQIKTMIDPMNFFRNEQSVPPLWSP

>GaCKX25

MRIAYLDRTVHETDGEPKPNGDVSAISKSIDLQGSVETGDKTTIASKDFGGLYSIKPLALIKPAGSGDISRVIKAASRIPRMTVAARGNGHSINGQAMTNGGLVIDMRSTEENHFRLLNINGSFFIDVSGGALWENVLTRCVSRFGLAPRSWTDYLSLTVGGTLSNAGVSGQAFRYGPQISNVTELEIVTGKGDITVCSETRNPELFFGSLGGLGQFGIITRARVKLQPAPDMVRWIRVVYTEFDEFTRDAEFLVSRDDGESFDYVEGFVFCNNDDPVNGWPAVPLDPVHGFNPGIIPQTGASVLYCLEVAFHYQKGDHPSSVDKAVAGLLKRLRFVEGLKSQVDLSYVEFLLRVKRAEEQAKANGIWDAPHPWLNLFISKSNIVDFDQTVFKKMVKDGIGGPMLIYPLMRSKWDNRTSVALPDSEIFYLVALLRFVSRGPSVEESVAQNREIVEWCIKEGLDFKLYLPHYQSKEGWKRHFGDQWTRFVERKASFDPMAILAPGQNIFKRTHLS

>GaCKX26

MGSPVCGFLKQNNIIFLRFFAILVLSCIPNRTNLCSNPSFDTPTIPPHSSSSSIPSSLKTLTLDGYFSFENLKHAAKDFGNICHYLPIAVLHPKSVSDISSTIKHILYMSSVTKLTVAARGRGHSLQGQAQAYQGVVINMESLDRPSMYIENGEVPYVDVSGGELWINILHETLKYGLSPKSWTDYLHLTVGGTLSNAGISGQAFRHGPQINNVYQLEVVTGRGEVVTCSDKENADLFYGVLGGLGQFGIITRARISLVPAKKMVKWIRVLYSEFSTFSNDQEHLISSNNSFDYIEGFVMINRTGLLNNWRSSFNPKDPIQASQFSSDGKILYCLEMVKYFNPEKIDVLNQDIEKLLSELNYIPSTLFLSEVSYVEFLDRVHLSEIKLRSKGLWEVPHPWLNLLIPKSRIFDFAEGVFGNIVKDNNNGPILIYPVNKAKWNNRTSMVTPEEDIIYLVAFLSSALPGTDGLEHIMTQNQHILDFCAKAQLGAKQYLPHYHTQDEWRAHFGTQWETFVQRKSAYDPLAILAPGQRIFQKAISIT

>GaCKX27

MGFSFWLARLRSCSNKSAFSKAFRDSFYHYKSQLNTCQKNLPSTIAEKTKSHAFSWSSCLLPLAFAVSAGSLTFQSHNNHPSLCEPSNLDSRKVTIGGKASTEFVVKGTHKEVPQELIDELKAICQDNMTLDYDERFYHGKPQNSFHKAVNIPDVVVFPRSEEDVSQIVKSCNKHKVPIVPYGGATSIEGHTLSPNGGVCIDMTLMKRVKALHIRDMDVVVEPGIGWMELNEYLEPYGLFFPLDPGPGATIGGMCATRCSGSLAVRYGTMRDNVISLKVVLANGIIVKTASRARKSAAGYDLTRLMIGSEGTLGVVTEVTLRLQKIPEHSVVAMCNFPTIKDAADVAIDTMLSGIQVSRVELLDEVQVRAVNIANGKNLPEVPTLMFEFIGTEAYSHEQTQIVQRIVSEHNGSDFVFTEDPEAKKELWKIRKEALWACFAMEPNFEAMISDVCVPLSNLAELISRSKQELDASSLVCTVIAHAGDGNFHTVILFDPNEEEHRREAERLNQFMVYTALSMEGTCTGEHGVGTGKMKYLEKELGIEALQTMKRIKTALDPNNIMNPGKLIPPHVCF

>GaCKX28

MKFPQFSMLPFLMVVVMLSLSGTTLSHPHWDFLHCLSLRLANYSTISKVIYVQNNPSYSSVLNASIHNTRFSTPATPKPFAIITPLKTSHVRSTLYCSKKHGFQLRIRSGGHDVEGLSYVSEVPFVILDLLNFRAIKVDTKNEVAWVQSGATLGELYYAIAAKTNTLAFPAGMCHTVGAGGHFSGGGYGTLFRKYGLAADNIIDARMIDVNGRILDRKSMGEDVFWAIRGGGGNTFGIVLAWKIKLVPVPAVVTVFTVNKNLEQNATKIFHRWQYIAHKLPDDLFMNVWITKVNSSQVGTKTVQATFRGMLLGGVDELITLIQDKFPELGLAEENCNEMSWIESVLYFGGLPLQSVDILLDKNALPTLSLKAKSDYVKEPIPESGIEGLMSMFLEEEADNAVAMIPAFGGKMDEIPENELPYPHRAGNLFEATYIVQWTKEQNAESGKYISWSRRLYSFMTPYVSKSPREAYVNYRDLDLGINNINDYTSYEQASIWGFKYFKNNFKRLVQVKTKVDPMNFFRHEQSIPPLLSPWKKKGN

>GaCKX29

MKFLQYSVLPFLLVVVILSLSGATLAHSHGDFLLCLSLHLANSSTISKVIYMRNNPSYSSVLNASIQNTRFSTPATPKPLAIITPLKTSHIQSTIYCSKKHGLQLRIRSGGHDFEGLSYVSEVPFVILDLLNFRAVKVDTKNKVAWVQSGATLGELYYGIAAKTKTLAFPAGICPTVGIGGHFSGGGYGILIRKYGLAADHIIDAQLIDANGRILNRKSMGEDLFWAIRGGGGNTFGIVLAWKIKLVPVPAVVTVFTVNKNLEQNVTKILHRWQYIAHKLPDDLYVDVGITKVSSSQAGKKTVQVAFIALFLGGVDELIPLVQERFPELGLAKENCSEMSWAESVLYFGRAPRKSLDILLDKNVVPRTIFKAKSDYVKEPIPESGIEGFLSMFLEKEADFAAMLMVPFGGKMEEIPENELPYPHRAGNLFQASYIVGWRKEENAESGKYISWIRRFYSYMATYVSKSPREAYFNYRDLDIGSNNISGYTSYQQNNFKRLVQVKTMVDPMNFFRNEQSIPPLSSP

>GaCKX30

MLVSFPGQNNMLVLRSFMILFFSCITIKINLCFPSIPSSLKTLSIDGHFDFEQVEHAAKDFGNRYSYIPLAVLYPKSVSDIATTVNHVWQIGGGSELKIAARGHGHSLQGQAQTHRGVVINMESLQGHKMQVHTGNFPYVDVSGGELWINILRESLKHGLAPKSWTDYLHLTVGGTLSNAGISGQAFRHGPQISNVYQLEVVTGKGEVVNCSEKQNSDLFYSVLGGLGQFGIITRARISLEPAPEMVKWIRMVYTDFATFTRDQEKLISGKSTFDYVEGFVIINRTGLLNNWRSSFNPQDPLQASQFKSDGRTLFCLELAKYFNHEDMVLVNQEIKTSLSQLNHIPSTLFISEVQYMEFLDRVHISEIKLRSRGLWEVPHPWLNLFIPKSKIHSFAQEVFGNILTDTSSGPILIYPVNKSKRRCFYLVAFLSSAVPSSTGNDGLEQILIRNKRILDFCEIAGLGVKQYLPHFSTQGEWKSHFGPHWEAFTRRKSTYDPSAILAPGQRIFQKLIAYS

>GaCKX31

MKASQCFSMSLFLLILLISCPWLISANPHLNNFLGCLDSFYSNDISKVIYTQNNASYSSVLNATIQNLRFSTPATPKPLVIVTPLQTSHIQATIRCSRTNGLNLRIRSGGHDFEGLSYVSQVPFVVLDLTNFRSVKIDVKNKVAWVQSGAILGEFYSEIARRSRTLAFPAGICHTVGVGGYLSGGGYGLLLRKYGLAVDNVIDAVFIDVNGRILKRKSMGEDLFWAIRGGGGGSFGVVLSWKVKLVSVPSTVTVFTIRKTLEENATNLVHQWQSVAHKLPGDIFSAVTMRKVNRSGKPTILVAFSSFFLGDTNALIPLMKARFPELGLKKEHCTEMSWIESILYFGQIQNKSIDVLLDRSYKSPLNAPSFKTKLDYVKNPIPKAGFEKIWSKLYEEDAETAAMAFIAYGGKMAEIPESATPFPHRDGNLYHIAYTVGWDGEENTKSQRYMNWIRKFYSFMTPFVSKSPREAYVSYRDDDIGTNNKKGETSYAKASVWGRKYFKNNFDKLIYIKTKVDPHNFFKHEQSIPVSV

>GaCKX32

MGKLKAVVVVTMISTVLLSILWRATLHLEDNENFVRCLLDHSHPSHPISSAIYTPKSSSFSSVLESYIRNLRFNESSIPKPFLILTALHESHIQAAVTCGKSHGVQMKIRSGGHDYEGLSYVSTLPFFLLDMFNLRSIDVDIETETAWVQTGATLGEVFYRIAEKSKTHGFPAGVCPTVGVGGHISGAGYGNLMRKYGVSADNVLDALIIDANGRLLDRQSMGEDLFWAIRGGGGASFAVVLAYKIKLVRVPETVTVFQVDRTLEEDATDIVDQWQHVAYNLPQELFIRLMLDVVVKSSGEKTLRASFVSLFLGDSESLLSIMKERFPKLGLSKSDCIETSWVKSVLFWSNIPLETDIQVLLDRTPQTLDYLKRKSDYVREPIPKAGLESLWKKMMELEKPRMYFNPYGGKMAEIAAEEIPFPHRAGNLWKIQYLANWNEAGIEAANRYIDLTRKLHEFMTPFVSKNPRQAFLNYRDADLGSSSHGKASYSEARLNGMKWFMGNFERLVQIKTEVDPTNFFSYEQSIPLLPQQVHLDDDI

>GaCKX33

MQKHKMKSNIPIPLLFLSILFSFSWAALGLTHQQQHNSFLQCLNRHSGDANSISTVIYTQTNSSFSSVLEFSLRNARFSTPNTLKPLVIVTPSHVSYIQATINCSRTHGLQIRIRSGGHDYEGLSYVSQVPFVIIDLINLRSIDVDAENKTAWIQAGATIGELYYRIAEKSSTLAFPAGICPIVGVGGHFSGGGYGMLMRKYGLAADQIIDAQLVDVNGKFLDKNSMGEDLFWAIRGGGAASFGVVVAWKVKLVPVPSTLTVFTVNRTLKENGTMLVHKWQSVAPKIHEDLYIRLFLRAVNSSQQEGKRTIQASFVSLYLGRADELVDLMQESFPELGIVKEDCIEMSWIQSIMYFPSDIPEDAPLEILLNRTGSAGIFKGKSDYVTQTIPETALEGLWQRFYEDETESLEILFSPYGGNMDDIPETETPYSHRAGILFNIHYVVGWSEEDASESQRYINFMRRLYRYMEPYVSKSPRRAYMNYRDLDLGTNNNGPYTSYKQASKWGLPYFDKNFNRLIHVKTLVDPTNFFRHQQSIPSLSRG

>GaCKX34

MEKWRATSNLRRSLKSILNRQLSSVSEFRYLNEKRSCQSSFNLIRDCKSLGQVNAIQHRCFSSASTLVQRNPSFSTLNFDDISYFKGLLGEKSVIQDEDRLETVNTDWMHKYKGSSKLLLLPRSTEEVAQILRYCNSRCLAVVPQGGNTGLVGGSVPVFDEVIVNVSSMNNIISFDKVSGILVCEAGCILENLISFLDNQGFIMPLDLGAKGSCQIGGNVSTNAGGLRLVRYGSLHENVLGLEAVLANGDVLDMLGTLRKDNTGYDLKHLFIGSEGSLGIVTKVSILTPPKLSSVNIAFLACNDYSSCQKLLMEAKRKLGEILSAFEFLDTEAMNLVLHQFDGVRNPLPASMHNFYILIETTGSDESYNREKLEAFLLSSMEDGLISDGVLAQDINQASSFWRIREGVPEALMKAGAVYKYDLSLPVEKMYDLVDDMRIRLGDMAKVVGYGHLGDGNLHLNVSAPEYDDKILEQIEPYVYEWTSKHRGSISAEHGLGLMKANKIYYSKSAETVQTMASIKKLLDPNGILNPYKVLPHSLNS

>GaCKX35

MAISKPLLVFFSLVFYNLSFSWAALDPTYQSLLQCLSEIIPSLNVSAVIVSNNNPSFASILESPIYNARFNRTATPKPAIIITPSDESHVGFQLKIRSGGHDYEVLSYTFNKPFFLLNMYNHWDVSVDIQDYELYYYIWEKSNVHRFPAGVCPTVGVGGHISGAGYGTMIRKFGLSTDYAIDAKIVDAIRGAGGANFGVVTAYKIKLVKVPLKATVFKVERFLDDNGTEVAFKRQTNAFTTQYESQANNVKVTIMGLYLGDINGLLTLLNKDFPELRLNKENCTEMTWIDSVLWWENFDLGTPPNVLLDRNNKGTKFVKRKSDYMVQNEKVGLTCNSYGGKMDEIDPKESAFPHRKGNFYKIKHSINWDDPNNEADIKYTTQAKAVHEFMTQFVSKNPMRAYLNYTDINIGSAKTWSYEEGKVYGESYFAENFDRMVDVKIVVDPNNFFRNEQSIPPRSTKTA

>GaCKX36

MAFPSISSLFLLLLVLHFSSSTTASTCHAKSFKLNPIQEKFIQCFKANSEIPIPVSTEFFTPNNASFSTVLQSTAQNLRYLEPSVPKPEFIIMPLNESHVQAAVICSKELGIHMRVRSGGHDYEGMSYVSETESPFILVDLSKLRSVKVDIENNSAWIEAGATIGEVYYRIYEKSKIHGFPAGLCTSLGVGGHITGGAYGSMMRKYGLGVDNVIDARIVDVNGRVLDRAAMGEDLFWAIRGGAGGSFGIILQWKIQLVPVPSTVTVFTITKSLQQNGTKIFHRWIEVADNLDDDLFIRVIIQTAMINGEKTVTTSYNSLFLGEADRLVEIMQQSFPELGLTRKDCIETSWIKSVLYIAGYPSNTPPEVLLQGKSTFKNYFKAKSDFVKSNIPETALEGLWKRFMEEDTPLMIWNPYGGMMARISESETPFPHRKGNKIMIQYVSAWQDGDKNESKHIDWIRRLYNYMAPYVSMFPRTAYVNYRDLDLGTNKNASTSFIEASGWGVKYFKDNFNKLVKVKTKVDPENFFRHEQSIPPLPVEARF

>GaCKX37

MSNSMAISLLFSLLFLNISISSAASNPTYQSLLQCLSQSINPSQNVSTILFSNTNPSYASVLQAYIRNARFNTSSTPKPVIIITPLEESHVSAAVICSQKVGFQLKIRSGGHDYEGLSYVSDKPFFVLDMFNLRSISIDMTDESAWVETGATLGELYYNIWEKSNVHGFPAGLCPTVGVGGHLSGAGYGTLMRKYGLSSDYIVDAKIVNVDGKILDRKAMGEDLFWAIRGGGAASFGVVLAYKVKLVRVPETVTVFRLERLLADNATDIALKWQSIAPTTDENLFTRMLLQPVTRNRQRTMRVTVIGQYLGNADGVVALLSKDFPELGLKNENCTEMTWIDSVLWWANFDAGTPPTALIDRNVNDADFLKRKSDYVQTPISKNGLESLWQKMVELGNVGLACNAYGGRMDEIDDKETPFPHRKGNLYKIQYSVNWNEPGNETEMNRTSQAKALHEFMTQFVSKNPRRAYLNYRDIDIGVAENWSYEEGKVYGESYFAGNYERLVDVKTAVDPNNFFRNEQSIPPRTK

>GaCKX38

MAISKLLLVFFSLVFFNLSFSWAASDPTYQSLLQCLNEIIPSPNVSAVIVSNNNPSFASILESRIHNARFNRTSTLKPAIIITPSDESHVSAAVICSQKVGFQLKIRSGGHDYEALSYTSDKPFFLLDMYNLRDVSIDIPDESAWVQTGATLGELYYHIWEKSNVHGFPAGVCPTVGVGGHIGGAGYGTMIRKYGLTTDYVIDAKIVNVNGKILDRKAMGEDLFWAIRGAGGANFGVVTAYKIKLVKVPEKVTVFRVERFLDDNGTEVAFKWQTVGATTDPNLFTRMLLQPNTKDKQTTVKVTVMGLYLGDINGLLTLLNKDFPELRLNKENCTEMPWIDSVLWWANFDLGTPPNVLLDRNNKDTKFVKRKSDYVQTPIPKDGLESLWQKMVQNEKVGLTCNSYGGKMDEIDTKESAFPHRKGNLYKIQYSINWDDPSNEADIKYTTQAKAVHEFMTQFVSKNPRRAYLNYRDIDIGSAKTWSYEEGKVYGESYFAENFDRLVDVKTAVDPNNFFRNEQSIPPRSTKTA

>GaCKX39

MTPPLSTPSLLPLLLVAFNICFSLAASNSIYESFVQCLKTRSNSSDNISDIVYSHSNATYEIVLEQYIRNARFNTSSTPKPVIIITPLTESHVSAAVICSNNIGFQLRIRSGGHDFEGVSYVSDQPFFILDMFNLRSISINMADQSVWVQSGATLGELYYRIWEKSKVYGFPAGVCPTVGVGGHISGAGYGNMVRKYGLSVDYVVDAKIVDVNGNILDRKAMGEDLFWAIRGGGGASFGVILAFNIKLVEVPETVTVFKLERTLEQNATDVVYKWQSVAPTTDDNLFMRMLVQPVTLNKQKTIKISIMALYLGDVNSVVPLLAEDFPELGLVTEDCFEMSWIESALWWASFGKGTSPTVLLDRESYHVKFMKRKSDYVKTPISKDGLQWLWKKMIELEEPGLVFNPYGGKMNEIKETETPFPHRAGNLFKIQYSINWKDMGIEADKRSRSLVNRLHSYMTSFVSKNPRSAYLNYRDLDIGITKNWSYQEGKVYGESYFNGNFERLVDVKTAVDPHNFFRNEQSIPPRTIKAWNEKNGGSIPPSTSKAWNKSKPYVMIILFTAIGII

>GaCKX40

MKASSYTCFIVSIFVLFSISSAASYDPVDFDAFLQCLPKHSDHSVSIAGAILTPNNASFQSIYQLRANNLRVLLSATSRPVAIITALHPSHAQAAVICAKRHGFQLRIRSGGHDYEGLSYISDVPFVILDMFNLKSIDIDMKTETAWVQAGATTGELYYSIAKTSEVHGFPSGVCTTLGIGGHFSGGGYGFLIRKYGLSIDNVIDAQLIDAKGRILDRKSMGEDVFWAIRGGGTTSFGIILSWRIKLVRVPPRVTVFNVQRTLEQGATELAYRWQQVAPKLPKDLFIRLQPVPINNGGNNKTVSVSFIGHFLGQADELLRLMNVRFPELGLTRNDCSEMSWVESALSWAGFPNGTSIDVLLNRVQVDRVFYKTKSDYYKAVIPKQGLETLWQVLMDIEDIFVQFNPYGGRMEEISESETAFAHRGGNLFKAQYGIQWSESDGGINATGRYVEMSRRLYNAMAPYASSNPREAFFNYRDLDVGSNESGETDFEVAKEYGAKYFRNNFLRLTGVKAKIDPENFFKNEQSIPPLPTPPSH

>GaCKX41

MKISSSIFSLISIFILLLISSATSSDFDGFLQCLLQQSNSSLPIIDAILTPNNSTFQSIYQLRANNLRTFLSAISRPVAIITALHPSHAQAAVICAKRHDFQLRIRSGGHDFEGLSYTSKVPFVILDMFNLNSIDIDMSTETAWVQAGATTGELYYRIAEKSNVHGFPSGVWTTLGIGGHFSGGGVTVFTVQRTLEQGATELAYRWQQVAPKLLKDLFIRLQPVPINNGGNNNTVRVSFIGHFLGQADGLLRLMNVSFPELGLTRNHCLEMSWVESTVYCANFPNGTSIDVLLDRVQENKVFSKSKSDYYKALIPKQGLETLWQGLMDIEDILVQMNPYGGRMEEILDSETSFAHRAGNLFMVLYRVQWSESHGGINATERYVEMSRRLYEVMAPYTSSNPREAFLNYRDLDIGSNESDETDFEDAQEYGAKYFRNNFIRLAKAKATIDPENFFKNEQSIPPLPH

>GaCKX42

MKASSYTCFIVSIFVLLSISSAASYNPVDFDAFLQCLPKHSDHSVSIVGAILTPNNASFRSIYQLRANNLRILSSATSRPVAIITALHPSHAQAAVICAKRHGFQLRIRSGGHDYEGLSYISDVPFVILDMFNLNSIAIDMKTETAWVQAGATTGELYYSIAQKSEVHGFPSGVCTTLGIGGHFSGGGYGFLIRKYGLSIDNVIDAQLIDAKGQILDRKSMGEDVFWAIRGGGTTSFGIILSWRIKLVRVPPRVTVFNVQRTLEQGATELAYRWQQVAPKLPKDLFIRLQPVPINNGGNNKTVRVSFIGHFLGQADGLLRLMNVRFSELGLTRNDCLEMSWVKSSLYWAGFPNGTSIDVLLDRVQVNRVFYKSKSDYYKAVIPKQGLETLWQVLMDIEDIFVQFNPYGGRMEEISESETAFAHRSGNLFIVLYRIQWSQSDGTGRYVEMSRRLYTAMAPYASSNPREAFFNYRDLDVGSNESGETDFEVAKEYGAKYFGNNFMRLAGVKAMVDPENFFKNEQSIPPLPTPPSH

>GaCKX43

MKISSSIFPLVSIFSLLLISSATSSDFDDFIRCLPQQSNSSVPITDAILTPNNSTFQSIYQLRANNLRTFLSATSRPVAIITALHPSHAQAAVICAKRHDFQLRIRSGGHDFEGLSYTSDVPFVILDMFNLNSIDIDVSTETAWVQAGATTGELYYRIAEKSNVHGFPSGVCTTLGIGGHFSGGGYGFLMRKYGVSIDNVIDAQLIDANGRILNRKSMGEDVFWAIRGGGTTSFGIILSWRIKLVRVPPRVTVFTVQRTLEQGATELAYRWQQVAPKLPKDLFIRLQPEPINNGGNNKTVRVSFIGHFLGQADGLLRLMNVSFPELGLTRNHCLEMSWVESTLYWANFPNGTSIDVLLDRVQENRVFSKSKSDYYKALIPKQGLETLWQGLMDIEDIFVQMNPYGGRMEEISDSETAFAHRAGNLFMVLYGVQWSESEGGINATERYVEMSRRLYEAMARYASTNPREAFFNYRDLDIGSNESDETDFEDAQEYGAKYFRNNFIRLAKAKATIDPENFFKNEQSIPPLPH

>GaCKX44

MKISSSIIPFISIFILLSISSVTPSDSDDFDDFLRCLPKQSDSSIPIANAILTPNSSSFQYIYQLRASNLRPLLSATSRPVAIITALHPSHAQAAVICAKRHDFQLRIRSGGHDFEGLSYTSDVPFVILDMFNLNSIDIDMSTEIAWVQAGATTGELYYRIAEKSNVHGFPSGVCTTLGIGGHFSGGGYGFLMRKYGLSIDNVIDAQLIDANGRILNRKSMGEDVFWAIRGGGTTSFGIILSWRIKLVRVPPRVTVFTVQRTLEQGATELAYRWQQVAPKLPKDLFIRLQPEPINNGGNNKTVRVSFIGHFLGQADGLLRLMNVSFPELDLTRNDCLEMSWVESTLYWAGFSNGTSIDVLLNRVAENKVFAKDKSDYYKAVIPKQGLETLWQVLMDIENIFVQMNPYGGRMEEISDSETAFAHRAGNLFKVLYGIQWSESEGGVNATARYVELSRRLYNAMAPYASSNPREAFINYRDLDIGSNESDKTDFEDAKEYGAKYFRNNFIRLAGVKAKIDPEYFFKNEQSIPPLPSH

>GaCKX45

MLNPKCVHFLTLLISLLSLPSPTISQSSSLTNFLHCLHYGSDPMVSQSIYIASNPAFQTILQARIKNRRFLNPETLKPVAIVVPTHIDHVQGTVICAKDNGLQIRIRSGGHDYEGLSYRSNVTFIILDMSNFRSIDIDIKTETAWVQSGATLGELYYHIANKTNTHGFPSGVCPTVGIGGHFSGGGYGNLMRKYGLSVDNILDIIAVDALGNVHDRASMGEDLFWAIRGGGAASFAVVVSYKIKLVRVPNKVTVFTKGFTLEQGATDLVHKWQQIAPNINEELFIRVKLQPSFINGNQTVTATFIGFFLGRREKLLPIISKTFPELNLTQQDCHEMRWVETTLFWAGFPIGTPIETLLNRTIWTPLFFKNKSDYVKNVIPKESLNKIWKMTMAMMDRNDINKTRFDLECSPYGGKMNVIPESNTPFPHRKGNLFLIQYSFSWIDEGNNVSFNNIEKLRKLYDGMAPYVSKDPRECFLNYRDLDIGSSRSNETSFDDAKIYGRKYFKDNYTRLTKVKASVDPNDFFKYEQSIPPIN

>GaCKX46

MGFLSLVIIFLLCISMANSDHLKQNKTILQCLTNHSIASPSISSVTFFPTDPSFTSTLQSYIRNLRFTSTTTPKPLFIVVPSHVSHIQASIICCKTHGLEMRIRSGGHDYDGLSYVSKAPFMILDLFNLRSVSVDNGTAWVESGATLGELFYAISQKSKTHGFPAGVCPTVGVGGHFSGGGYGNMMRKFGLSVDNVIDAKLVDVNGNVLDRESMGEDLFWAIKGGGGASFGIIISWKIKLVSVPEIVTVFKIEKTLEQGVTGIVHKWQYIADKIDPNLFVRVVLLPVNKKHLQSIKAKFIGLFLGNGQELISLMNETFPELGLSFDQCIEMSWIESILFWSNYPKGTSLDVLLDRQPQQEKYLKKKSDYVQEPISKENLEGIWNKMIELKRPALTLNPYGGKMSEISEFETPFPHRAGNIYKIQYSVTWKDDGVEASDRSLDQIRKLYDYMTPYVSKSPRSSYLNYRDVDIGINENGNASYSEGVIWGRMYFKGNFQRLVQVKSKVDPGNFFRYEQSIPCLGSWKSITAE

>GaCKX47

MFRSLSLKRSFKTHLKTLIHCPQQPPPPPPPPSPVLFSSIRALSTASSPPSSSSDSELRKYLGYTALLAFCGVATYYSFPFPENAKHKKAQLFRYAPLPEDLHTVSNWSGTHEVQTRHFHQPENLKQLEELVKESNEKRVKLRPVGSGLSPNGIGLARGGMVNLALMDKVLEVDKEKKRVRVQAGIRVQQLVDEIKDYGITLQNFASIREQQIGGILQVGAHGTGAKLPPIDEQIISMKLVTPAKGTIELSKEKDPELFYLARCGLGGLGVVAEVIIQCVERQELVEHTTVSNLKDLKKNHKKMLSESKHVKYLYIPYTDTVVVVTCNPVSKWRGPPKFKPKHTTDEAMQDIRELYKESLKKYRARDITNKSSDSDEPNINEFSFTELRDKLLSLDPLNKDHVMKVNHAEAEFWRKSEGYRVGWSDDILGFDCGGQQWVSETCFPAGTLSKPSMKDLEYIEELKKLIETNELPAPAPIEQRWTARSQSPMSPASSSAEDDIFSWVGIIMYLPTMDARQRKEITEEFFHYRHLTQSQLWDKYSAYEHWAKIEVPKDKEELEALQARLKARFPVDAYNKARRELDPNRILSNNILEKLFPLSDTV

>GaCKX48

MKSLQFSMLLLSLVLAALLSFSTGALPQEDFLRCLSLRSNDSSTISSVIYTRNNPSYSTVLESTIRNLRFNSTNTPKPLVIVTPSRTSHFQATIYCSRKHGLQIRTRSGGHDYEGLSYVSKVPFVVVDLVNFRTVDVDVENRVAWVQAGAILGEIYYRIAEKSRTLAFAGGIFHSIGVGGYISGGGFGLLFRKYGTAGDNVIDAQFIDVNGRILDRKSMGEDLFWAIRGGGGGSFGIVLAWKLKLVPVPAIVTVFSVNRTLEQNATQLILRWQEIAHQLPDEMNPDVTMLSVNSTQDGSKTILASFSSLFLGTIDELLPIMQQRFPELGLSRQDCSEMSWIESVLYFNQLQNQPLEILLNRTFRTSVGGQYYKIKSDYVKEPISETALNGLFSRLSDEEASSAFIIFMAYGGIMDRIPEDATPFPHRAGNLYKIYYDVNWQEQDNVNSQKYIDWSRRVYNYMTPFVSKSPREAYANYRDLDIGSNNVGITSYRQASVWGRKYFKNNFDRLVQVKTKIDPQNFFKHEQSIPPLH

>GaCKX49

MKFLQFSMLLLPILLDALLSFSMGASTHQDFLQCLSLRSNDSTSISNVIYTRNNSSYSFVLESTIRNLRFNLTDTPKPLVIITPSRTSHFQATIYCARKHGLQIRTRSGGHDYEGLSYVARVPFVVVDLVNFRSVDVDVENRVAWVQAGAILGEVYYRIAEKSRTLGFAGGIYFTIGVGGHISGGGFGLLFRKYGLACDNVIDAQFIDVNGRILDRKSMGKDLFWAIRGGGGGSFGIVLAWKVALVPVPPTVTAFSISRALEQNATQLILRWQDIAHQLPDEMNPDVTMFSFNSTQDGRKTILVSFSSLFLGTIDELLPIMQQRFPELGLSGQDCIEMSWIEAVLYYNQLQNQPLETLLNRTFRTPSGGQYYKIKSDYVKEPISETGLNGLFSRLSDEEASSAVIIFMAYGGIMGRIPEDATPYAHRAGNLFKIYYNVNWQEQDNVNSQKYIDWSRRVYKDMTPFVSKSPREAYANYRDLDLGSNNVGITSYTQASIWGRKYFKNNFDRLVQVKTKIDPENFFKHEQSIPPLF

>GaCKX50

MGASYHEDFLQCLSLRSNDSTSISNVVYTRNNSSYSSVLESTIRNLRFNSTDTPKPLVIVTPSRTSHFQATIYCARKHRLQIRTRSGGHDYEGLSYVAKVPFVLVDLVNFRSVDVDAENRVAWVQAGEIVGEVYYRIAKKNRTLAFPGGVYYTIGLGSFISSRGFGLLFRKYDTGGDNVIDAQLIDVNRRILDRKSMGEDLFWVI

>GaCKX51

MTPYVSKSPREAYSNYRDLDIGANKINNDKESYAEARVWGLKYFKNNFDRISASAQPAEEFLRCLSIRFHNSSSIAKLVYTHHNSSYFSVLKSSAQNFRFTTPSTSTPLAIVTPFHASHIQATVYCCRKHGLQVRTRSGGHDFEGLSYTTAYKVPFVVIDLVNLRSVQVNVEKATGWVESGATVGELYYEIARKSRTLAFPAGIGHTVGIGGQLSGGGLGSLFRKYGLASDNVIDARLIDAYGRILDKKSMGEDLFWAIRGGGGGSFGIVLAWKLKLVPVPANVTACTVSKTLEQNATKLVHQWQSIARKFPKEIQSSIAIARVNSSEDGKMTIQASYGSVFLGSIDELIPLMEEKFPELGLVKEDCLEMTWAESVLYSALSIIGLPLETLLNRTQKSALSQTFFKAKSDFVKQPISESGFEGLWPKFYEDEAKSAVMVLVAYGGKMDEIPETEYPYPHRAGNLYSILYVVNWEEEENKNSEKFMNWMRRVYSYMTPYVSKSPREAYVNYRDLDIGTNKFNDEKGSYTEGKVWGLKYFKNNFDRLVYVKTKVDPQNFFSHEQSIPPLS

>GaCKX52

MFPFLFVVLLSLSWRINASALPVQEFLHCLSLRSPNSSIAKLVYTQNNSSYSSVLKSAAQNFRFTTPSTPTPLAIVTPLHASHIQAAVYCCRKHGLQVRTRSGGHDFEGLSYASAYKVPFVVIDLVNLRSVRVNVEKATAWVESGATVGELYDEIARKSKTLGFPAGICPTLGVGGHLSGGGYGLLFRKYGLGADNVIDARLIDVKGRILDKKSMGEDLFWAIRGGGGGTFGIVLAWKLKLVAVPANVTVFTVRKTLEQNATKLVHRWQSIAHKFPKEMHLSIAIARANSSEDEEKMTIQASFTSTFLGSIDELIPLMEEKFPELGLVKEDCLEMSWAESNLYSFLFLLRSPSETLLNRNQKSSISKTFFKAKSDFVKQPIPESAFEGLWSKFYEDEAKSAIMVFVAFGGKMDEILETEYPYPHRAGNLYTILYTVDWEEKENINSEKFMSWMRRVYNYMTPYVSKSPREAYINYRDLDIGTNKINNRKRSYAKARVWGVKYFKNNFNRLVNVKTIVDPQNFFRHEQSIPPLFLLWKRSDHKKHLSS

>GaCKX53

MFPSLFVILLSVSWKSSSTLAYPSEGFLHCLSLHFANSTSISKLVYTQRNSSFSSVLKSSAQNFRFTTPSTPTPLFIITPLQPSHIQAAIYCSRIHGLHVRTRSGGHDFEGLSYTTANYIIPFVVIDLVNLRSVQVDVEKATAWVESGATIGELYYGIAQKSRTLAFPAGLFYTVGVGGQFSGGGYGPLFRKYGLAADNVIDARLIDAKGRILDRKSMGDDLFWAIRGGGGGSFGIVLAWKLKLVAVPANVTVFTVRKTLEQNATKLVHRWQSIAHKFPKELLMSIFISSVKSSEADKKMTIQANFSSMFLGSIDELVPLMEERFPEFGLVRDDCLEMSWVEAILSAQGRVQLETLLERHQKTGVSQTFFKAKFDYVKQPIPEMGLEGLWPMFYEEEAKMANIFLVAYGGKMDEIPETEIPFPHRAGTIYSIIYVVDWDEKDNRNSKRFLNWIRRVYDYMTPYVSNSPREAYVNYKDLQIGSNNMFSCKGSYAQAKIWGRKYFKNNFDRLVYVKTKVDPENFFRHEQSIPPLSCF

>GaCKX54

MFPFLIVVLFSLSWKISDSTQPAEEFLHCLSLRFHNSSSISELVYTQHNSSYSSVLKSSAQNFRLSTPSTPTPLVIITPLHASHIQATVHCCKKHGLQLRIRSGGHDFEGLSYTTTYEVPFVVIDLLNLRSVRINVEKGTAWFESGAKVGELYYEIAKRSRTLAFPAGIGHTVGVGGHLSGGGFGLLFRKYGLAADNVIDARLIDVKERILDRKSMGEDLFWAIRGGGGGSFGIVLAWKLKLVAVPAIVTVFTVSKTLEQNATKLVHRWQYIAHKLPKEIHMSIGISRVKSNENEKMTIQASFRAVFLGSTDELLPLMEEKFPQLGIVKEDCFEMGWAESNLYSTQFPIGVPLETLLNRNRKSILSKLFFKAKSDYVKQPIPETTFEGLWTKFYKEEAQSAIMVFVAYGGKMDEILETETPFPHRAGNLYSISYMVDWEEEENKNPEKFMSWIRRIHNYMTPYVSKSPREVYVNFRDLDIGTNEINDKENSHEQAKIWGIKYFKNNFDRLVNGKTMIDPENFFRHEQSVPPLPCLLKKGANSNSLGSL

>GaCKX55

MPAIPMLFSNKFSVLLLWKCLCLLVVLVGCNPPGEPVKCSTKDSNCTVTNSYGMFPDRAICRAGNVAYPTSEQELVSIVSAATKSKRKMKVVTHFSHSIPKLVCPDGQDGLLVSTKNLNRVLKTNTEAMTMTVESGVTLRQLITEAAKAGLALPYAPYWWGLTIGGLLGTGAHGSSLWGKGSSVHDYVVEMRIVSPAKAEDGYAKVWVLNERDKDLDAAKVSLGVLGVISQVTFKLQPLFKRSITYVRKDDTDLGDEAVTFGKLHEFADIFWYPSQRKAIYRIDDRVPINVSGNGVYNFTPFRSTLSLVLALVRSSEETQESSGDAEGKCLNAKLMTSTLQSSAYGLTNNGAIFTGYPVIGFHDRLQSSGTCLDSLEDSLITACPWDPRIKGEFFHQTTFSIGLSVVKSFIQDVQKLVSMDPKSLCGLELYNGILMRYVKASTAYLGKQEDAIDFDITYYRSKDPMAPRLYQDVLEEIEQMALFKYKALPHWGKNRNLLFDGVMKRYKNGGEFLKVKNKYDPWGLFSSEWTDQVLGLRNGVTILKEGCALEGLCVCSQDVHCAPSKGYLCKFGKIFSDARVCARVNTKT

>GaCKX56

MPIRPVLYSNKFNELVLLKCLFLLVALVGCSPPGEPVKCSTKDSNCTVTNSYGAFPDRTVCRAGNVVYPISEQESVSILSAATEVQRKMKVVTHFSHSIPKLVCPDGQDGLLISTKNLNRVVKINLTAMTMTVESGVTLRQLINEAAKAGLALPYAPYWWGLTIGGLLGTGAHGSSLWEKGSSVHDYVVEMRIVSPAKAEDGYAKVWVLNESDKDLDAAKVSLGVLGVISQVTLKFQFLFKRSITYVMKDDTDLGDKVVTFGKLHEFADMFWYPSQRKVAYRIDDRVPVNVSGSGVYYFTPFRSTLSSVLSVIRSVEETQESLRDTEGKCLNAKLVTSTLQSSAYGLTDNGVIFTGYPVIGFHHRLQSSGTCLDSHKDSLITACPWDPRIKGEFFHQTTFSIDLSVVKSFIQDVQKLVSMDPKSLCGLELYNGIVMRYVKASTAYLGKQEDAIDFDITYYRSKDPMAPRLYQDNRNLVFDGVMKRYKNGGEFLKVKNKYDPWGLFSSEWTDQVLGLRNGVTVLKEGCALEGVCVCSQDVHCAPRKGYLCKPGKIFLDARVCARVNTKN

>GaCKX57

MQRNMLFLRSFMILVLSCIAIKINLCFPNILSSLKTLPIDGHFNFEQLHHAAKDFGNRYSFLPLAVLHPNSVCDIATTVKHIWQMGPGSDLTVAARGHGHSLQGQSQAHGGIVINMKSLQGLKMQFHIGNLPYVDVSGGELWINILREGLKHGLAPKSWTDYLHLTVGGTLSNAGISGQAFRHGPQISNVHQLEVVTGKGEVVTCSEKQNSDLFHGVLGGLGQFGIITRARISLEPAPEMVKWIRVLYTDFATFTRDQEELTSGESTFDYVEGFVIINRTGLLNNWRSSFNPQDPVQASKFKSDGRTLFCLELAKYFNRDETAVVNREIHSSLSQLNHIPSTLFVSEVPYIEFLDRVHISEIKLRSKGLWEVPHPWLNLLVPRSKIQTFAQQVFGNILTDTSNGPILIYPVNKSKWDNRTSVVTPDEDVFYLVAFLSSAVPSSTGNDGLDHILIQNKRILEFCEIARLGVKQYLPHYSTQGEWKAHFGSRWEVFVRRKSSYDPLAILAPGQRIFQKAVPYSQ

>GaCKX58

MVGKITRAYERELKDGDVKMTSPKFGMSILFFFLRISFCSSTDQSFQQCFSSHLPPSKITYDVIFTQNSSQYSSILQSSIRNLRFSNASKPRYLVTPYNENHIQATIICSKEHHMHVRVRSAGHDYEGLSYISDVPFIVIDLFHIRSVMVDIKNEFAWVGAGATLGELYYSISAKSNVHGFPAGSCPTVGVGGHISGGGFGTIFRKYGLAADNVIDAKMIDVNGNVLDRKSMGEDLFWAIRGGGGASFGVIFSWKLKLVRVPPTVTVFKTLKTLEQGATKLVQKWQNIAYKFHHDLFVHAVIQVTNPNSNQNPTVQVSFDCLFLGTTERLLSSIQRSFPELGVTQENCTEMSWIQSVLYFAGHSIAESADVLLNRGTQSTQSFKGKSDYVKEAIPKMGLEGLFKMVAEEETSMLILTPYGGRMKQIKSSATPFPYRSEYLYGIQYMISWDVAEETGKRIGWMRRLYKYMEPYVSTAPRAAYFNYRDLDLGRNSYPNTSYVEASQWGLKYFNHNFNRLVRVKTLADPHNFFWNEQSIPVLRLE

>GaCKX59

MRENERKRHSFTPKMSDLQAPLRPKRKKGLVDFLVQFRWVFVIFFVLPFSALYYFLIYLGDVRSEMKSYKQRQKEHDENVKKVVKRLKQRNPKKDGLVCTARKPWIAVGMRNVDYKRARHYEVDLSAFRNILEIDKERMIARVEPLVNMGQITRVTVPMNLSLAVVAELDDLTVGGLINGYGIEGSSHIYGLFSDTVVAYEIVLADGRVVRATKDNEYSDLFYAIPWSQGTLGFLVAAEIKLIPVKEYMRLTYTPVVGNLQDLAQGYMDSFAPRDGDQDNPEKVPDFVEGMVYSPTEGVFMIGRYASKEEAKKKGNKINNVGWWFKPWFYQHAQTALKKGEFVEYIPTREYYHRHTRCLYWEGKLILPFGDQWWFRFLLGWLMPPKVSLLKATQGESIRNYYHEMHVIQDMLVPLYKVGDALEWVHHEMEIYPIWLCPHRLFKLPVKTMVYPEPGFEQHHRQGDTPYAQMFTDVGVYYAPGPVLRGEVFDGAEAVRKMEQWLIKNHSFQPQYAVSELNEKDFWRMFDADLYEHVRRKYGAVGTFMSVYYKSKKGRKTEKEVQEAEQAHLETAYAEAD

>GaCKX60

MIACLGRIVQDTDADSIPDDDVSTLSESLDLQGTIESGGITGVAGKDFGGLYSVKPLALIKPSGTEDIARVVKAASQTSHLTVAARGNGHSINGQAMADGGFVIDMRSTEENHFKPLTIDGSHYIDVSGGALWEDVLRRCVSMFRLAPRSWTDYLSLTVGGTLSNAGVSGQAFRFGPQTSNVTELEVVTGKGEITVCSATQNSELFFGALGGLGQLGIITRARVKLQPAPDMVRWLRVVYTEFEEFTRDAEFLVTQEEGESFDYVEGFVFSNSDDPINGWPSVPLDPDHEFNPAYIPQTASSVLYCLEVALHYRNSDRPSTVDTAVSRLLERLGFIQRLKFQLNVSYVEFLLRVKQVEEHAKANGNWDSPHPWLNIFISKSSIVDFDRTVFRKMLKDGVGGPMLIYPLLRSKWNSRTSVVLPEGEIFYIVALLRFVPKGPTVEKLVAQNHEIIKWCNKEGLDFKLYLPHYQSKEDWKRHFGNQWTRFVERKTSFDPMAILAPGQKIFKRTHTIKP

>GaCKX61

MVMSFQFPAYFTAIFIITRVMSIMKISKPLDVHHKDIRAVDLATKLSVDPSAVESASRDFGGIVKAEPEAVLHPSAPQDIAALIKFSYSNSVPFGIAAKGHGHSVRGQAMAENGVVVDMRSMANKRRNGTGIRVSIDRLYADVGGEQLWIDVLNATLEYGLAPVSWTDYLYLTVGGTLSNAGISGQTFRYGPQISNVLEMDVITGKADFLTCSPRMNSELFYAVLGGLGQFGIITRARIPLQPAPKRVKWVRLLYDDFSSFTKDQELLISKNGRKDKSALDYLEGSLLMDQGSPDNWRSSFFPNKDHPKIISLITKHGIIYCLEIVKHYDDRTKHTVDKEMEQVLQGLNYMPGFIFGKDVGYEEFLNRVRSGELKLKSQGLWDVPHPWLNLFIPKSQISDFNNGVFRGIVLERNITTGPVLVYPMNSQKWDDRMSAVIPDEEIFYTVGFLHSSGFDTWKAFEDQNKDIMRFCNKTGIMLKQYLPHYSTKEEWVHHFGSKWKVFQHRKYQFDPRMLLSPGQRIFNNN

>GaCKX62

MAFSSTMILPLLLVLLSAFSATSKSVQENFMQCLDANSEHPIPISAFCFQTNSSFTSVLNSTAQNLRYLMPLVPKPEFIFIPVYESHVKSAVICAKKLAIHLRFRSGGHDYEGLSYASEIETPFILLDLIQLRSINVDIDDNSAWVQAGATVGEVYYRVSEKSKTHGFPAGLCSSLGIGGHITGGAYGSMMRKYGLGADNVLDARIVDVNGEILDRAAMGEDLFWAIRGGGGASFGVILSWKIKLVAVPETVTVFTVPKTLEQGATKILYRWQQVADKLDDDLFIRVVIQVTKTSQKGKRTVTTAYNALYLGDAERLLQVMDQSFPELGLARKDCIETSWIKSVLYIAGFPSETPPEVLLEGKSLFKNYFKAKSDFVQQPIPETALGRLWEMLLEEESPLMIWNPYGGMMANISDSAIPFPHRRGNLFKIQYVTSWYEGSKGATRKHMDWIKGLYDYMSAYVPTSPRAAYVNYRDLDLGMNHKNASFTEASVWGAMYFKGNFRRLVKIKSKVDPGNFFRHEQSIPVVLE

>GbCKX01A

MATMLLLTFLISSLIMVTVGLAIDFMEILRLGIDGQLSVDPSDVETASLDFGLLTRGQPLAVLHPVSAQDIARVVKAAYGSNQGMTVSARGHGHSINGQAQTTNGVVIQMSGWKGGNKPPRPHVWAEERYVDVWGGDLWIDVLKSTLEYGLAPKSWTDYLYLSVGGTLSNAGISGQAFNHGPQISNVYELDVVTGKGEVLTCSEDENTELFHAVLGGLGQFGIITRARISLEPAPNMVRWIRVLYSNFSAFTSDQEHLISLHAEPSNQKFDYVEGFVIVAEGLINNWRSSLFSPQNPVKISTLFPTGGVLYCLEIAKNYHESTAQTIDQEVEFLLKKLNFISTSVFTTDLLYVDFLDRVHMAEMKLRSKGLWEVPHPWLNLFIPSSKIAEFDKGVFKGILGNKTSGPILIYPMNKNKWDHRSSVVTPDEGLFYLVALLRSALDSGEETQSLEYLNNQNRQILRYCDEAGIKVKRYLPHYTTQQEWMDHFGNKWDRFYEMKMEFDPRHILASGQRIFTPTFPSLSNMPS

>GbCKX02A

MATASIYRPRKRPTSSLIQEKFIQCFTGSSQFYIPLSTAFFTPNNASFTSVLQSTSQNLRYLVPSMPKPELIITPLHESQAQASVICAKRLDIHLRVRSGGHDYEGLSYVSQIESPFVIVDLSKLRLIKVDIQDNSAWVEAGATIGEVYYRIAEKSNIHGFPGGLCTSLGIGGHITGGAYGSMMRKFGLGADNVIDARIVDVNGRVLDRAAMGEDLFWAIRGGGGASFGIILEWKIKLVPIPATVTVFTVTKSLEQGATKLLYKWQTVADKLDEDLFIRVIIQTANAGKNNAKTVTTSYNALFLGDAERLLRVMQQSFPELGLTRKECTETSWIKSVLYIAGYSSNTPAEILLQGRSTFKNYFKAKSDFVKEAIPETALEGLWKRLLEEDSPLMIWNPYGGMMGRISESQIPFPHRQGIKFKIQYLTLWQVEDNNASKHFDWIRRLYNYMAPYVSMFPRGAYVNYRDLDLGMNKNINTSFIEASLWGVRYFKDNFMRLVKVKTRVDPNNFFRHEQSIPPLPVQARY

>GbCKX03A

MKDSNSATLPLVTIVLFLSLSWRATSDSSSQVDKFLQCLANSSALMLESIYTPSNSSFESALQAYIRNHRFLTPETPKPVAIVAPTHVSHVQATVVCAKDNGVQIKVRSGGHDYEGLSYRSNVTFVILDMFTLRSIDVDVDNEVAFVQAGATIGELYYKIANESKDHAYPAGVCLSLGTGGHFNSGGYGNMMRKYGLSVDNILDAQVVGADGNILDQASMGEDLFWAIRGGGGASFAVIPSPLNTGGGDGENPDEDMVLRIENDRSTKNRALDTNCWVFEQFIRSKYEAQSRT

>GbCKX04A

MRNVDYKRARHFQVDLAFRNILEIDKVQMIARVEPIVTKGQSITVPMNLSLAVVADLDYLTIFGLINGNGIAGNSHIYGLFSYTVVAYEKVLAVALLELQTMMNILISSMLPLELAKGYMDSFTPKDCDEDQDNVAKVPEFVEAIIYSPTKVVFMTGRYASKEETKKKGNNIKSRFLLGWLMPPKVSLLKVGEAIRNYYHEMHVIQDMLVPLYKVYPIWLCPRQLFKVPIKTMVYPEPGFKHHCRQDDTPYARMFTYMGPVLRGEVFDGAEAVSKMEQWLIKNHSFQQHYAVSELNEKDFWRMFDVDLYEYARKKYGAVGTFMSGYYKSKKGRETEGSARS

>GbCKX05A

MKVLQLSVLSFLIVILSLNGATLAHPYGDFLHCLSLRISNSSTISKVIYTQNNPSYSSVLNASIHNTRFATPTTPKPYAIITPLKTSHVQSTIYCSKNHGFQLRIRSGGHDVEGVSYVSQVPFVVLDLVNFRDVKVDTKNEVAWVQSGATTGELYYGIASKTQTLGFPAGICHTIGIGGHLSGGGFGILGRKYGLAADHVIDVKLVDANGRVLNRKSMGEELFWAIRGGGGNTFGVVLAWKVKLVPVPPVVTVFTVNKNLEQNATKIFHRWQYIAHKLPNELFTAVWVMKVNSSQVGKKTVQAGFRGMFLGGVDELIPLIQHEFPELGLAKENCTQMSWVQSILYFGGLPIQPVNILLNRSALPVSSLKAKTDFVREPMSETGIEGFMNMFLEEEADFAITMIEAFGGRMDEIRENELPYPHRAGILFESTYIVQWTNEAEAGRYISWIRRLYSYMASYASKSPREAYYNYKDLDLGTNNIVGYTSYEQASVWGLKYFKNNFKRLVQIKTKVDPMNFFRNEQSTPPL

>GbCKX06A

MKFLQFSVLPFLMVILSVSGANLTHHPHRDFLRCLSLRIENSCTITYTHNNPSYPSVLNASIQNTRFSTPTTPKPYAIITPRKTSDVQSTIFCSKNHGFQLRIRSGGHDVEGVSYVSQVPFVVLDLVNFRDVKVDTKNEVAWVQSGATTGELYYGIAAKTQTLGFPAGICHTIGIGGHLSGGGFGILGRKYGLAADHIIDAKMVDANGRVLHRKSMGEDLFWAIRGGGGNTFGVVLAWKIKLVPVPPVVTVFTVNKNLEQNATKIFHRWQQIAHKLPNDLFTTVWVMKVNSSQVGKKTVEASFKGLFLGRIDELIPLIQYAFPELGLARENCTQMSWVQSVLYFGALPIEPVEILLNRSALPRLSLKAKTDYIREPMSEAGFEGFMNMFLEEGTDLAITMMEAFGGKMDEIRENEIPFPHRSGILFESVYIVQWINEEDAGLCINWMRRLYNYMSSYASKSLREAYYNYKDLDLGINNVNGYTSYEQASVWGLKYFKNNFKRLVRVKTMIDPTNFFSNEQSIPPLLSP

>GbCKX07A

MIAYLGRIVHDNDAESKLDDDVSSISKSLDLQGSIENGDVSSLASKDFGGLYSVKPLFLIKPSGAEDISRVVKLASRTSNLTVAARGNGHSINGQAMAEGGFVIDMRSTEKNHFEFLPINGSHYIDVSGGALWEDVLTRCVSRYGYAPRSWTDYLSLTVGGTLSNAGVSGQAFRYGPQTSNVTELEVVTGKGEITICSETLNSELFFGVLGGLGQFGVITRARIKLQQAPDMVRWIRVVYSEFEEFTRDAEFLVTQKEGESFDYVEGFVFCNNDDPFNGWPSVPLDPGHEFNPTHISQTAGSILYCLEVAFHYRNSDHPTVDTAVNGLLGRLRFVEGLKSQVDVSYTKFLLRVNRAEEQVKANGPGDGPHPWLNLFVSKSDVVNFNRTVFKTMLKDGVGGPMLIYPLLRSKWDDRTSVVLPEGEIFYIVALLRFVPNGPSVEKLVAQNREIVNWCIKVGLDFKLYLPHYQSKGDWERHFGNRWSRFVERKASFDPMAILAPGQNIFRRDPSNIIISREF

>GbCKX08A

MAIVAALHESHVQAAVICAKESGLQVRTRSGGHDYEGLSFSSSVPFVIIDLSNLRSIKIDMKTETAWVQAGATTGELYYRIAEKSKVHAFPAGVCTTLGIGGHFTGGGYGNMLRKFGLSIDNVVDAQLIDAKGRILNRKSMGEDVFWAIRGGGGTSFGIILSWKIKLVRVPPKVTVFQVAKTLEQGATVLVHKWLQVSHKLDKDIFIRIMPVTVAGTGNGNSTVRVSFIGHYLGRINRLLPLVNASFPELCLQRKDCTEMSWIESTLYWAGFPIGTSTDVLLNRVPNKVFFKTKSDYLKNVMPKAGLETLWKVMMEIGNMRMQMSPYGGRMAEISESETAFAHRAGTLYIVQYTAHWSEGSSEATKKYVELMRKLYAEMAPYVSSKPREAFLNYRDLDIGSNNTDFEAAKVYGAKYFKGNFQRLAEVKAKIDPHNFFKNEQSIPPFPSF

>GbCKX09A

MATKLLLTFAICRLIVTVGLTLDPTELLLLGVDAQLSVDPTDVKAASLDFGLLIGAQPPLAVMHPASSQDVAQLVKAAYGSNFGFTVSARGHGHSINGQAQTANGVVVQMSGSKGGSGMASGRKPPHPRVWPQERFVDVWGGELWIDVLRSTLQHGLAPKSWTDYLYLSVGGTLSNAGISGQAFNHGPQISNVHELDVVTGKGELLTCSEEQNSEMFHAVLGGLGQFGIITRARISLEPAPQRVRWIRVLYSNFSIFTSDQEYLISLHEQPTSQKFDYVEGFVIVDEGLINNWRSSFFSPHNPVKISSLDPNGGVLYCLEIAKNYHESTASTVDQEVESLLKKLNFIPASVFTTDLPYVDFLDRVHKAELKLRSKGLWEVPHPWLNLFVPKSKIADFDKGVFKGILGNKTSGPILIYPMNKNKWDHRSSAVTPDEDVFYLVALLRSALDNGEETHSLEYLTNQNRQILRFCDEAGITVKQYLPHYTTHQEWVDHFGNKWDRFYRLKMEFDPRHILASGQQIFTPTNMASWR

>GbCKX10A

MHFLRNLIILFLICIAIKINLCVPTIPSSLKTLPIDGHFDFKQVHHAAKDFGNRYSFLPSAVLHPKSASDIATTVKHIWEMGPGSHLTVAARGHGHSLQGQAQAHRGVVINMESLQGPKMKVHTGNFPHVDVSGSELWINILYETLKHGLAPKSWTDYLHLTVGGTLSNAGISGQAFRHGPQISNVRQLEVVTGKGEVVNCSEKQNSDLFYSVLGGLGQFGIITRARISLEPAPKMVKWIRVLYTDFATFAKDQEMLISGESTFDYTEGFVIINRTGLLNNWRSSFNSNDSAQASHFKSDGRTLFCLELAKYFNPEEMAIVNQEIMTSLSQLNHIPSTLFQSQVSYIEFLDRVHISEIKLRSKGLWEVPHPWLNLLIPRSNIHIFAQLVFGNILTNTSNGPILIYPVNKSKWDNRTSVVLPEEDVFYLVAFLSSAAPSSTGSDGLEHILSQNKRILELCEIDGLGVKQYLPHYSTNGEWRSHFGPQWEAFVHRKSTYDPLAILAPGQRIFQKGFG

>GbCKX11A

MATKLLLTFAICRLIVTVGLTLEPTELLRLGLTVDPVDVESASVDFGLMTKVEPLAVLRPSSAEDVAQLVKAAYESSHGFTVSARGHGHSINGQAQTGTGVVVQMSGVRSSGKPSVWGGDMYVDVWGGELWIDVLKSCLEYGLAPKSWTDYLYLSVGGTLSNAGISGQAFHHGPQISNVHELDVVTGKGELMTCSKEQNPELFHAVLGGLGQFGIITRARISLEPAPQRVRWIRVLYSNFSAFTKDQEYLISLHGNQKFDYVEGFVIVDEGLINNWRSSFFSPRNPVKISSLGSNNNGGVLYCLEITKNYHESTAETIDQEIEALLKKLNFIPTSVFTTDLPYVDFLDRVHKAELKLRSKGMWEVPHPWLNLFVPKSKIADFDRGVFKGILGNKTSGPILIYPMNKNKWDDKSSVVTPDEDVFYLVAFLRSALDNGEETQSLEYLTNQNRLILKFCDEDGIKVKQYLPHYKTQSEWKEHYGSKWDRFQRMKMKFDPRHILASGQNIFTPTFLSSSKMASW

>GbCKX12A

MAVALPSFFTAIMIMSRLMAFIGISKNNDMSSKLQALDIAPKVSHDPSAIESASQDFGHIVKAAPQAVLLPSSPRDIASLVNFSYSNSVPFSIAARGNSHSLNGQAMAKNGVVIDMTSMKSGNGTGIRIASDGSYVDVGGQQLWIDVLNATLGLGLTPVSWTDYLYLTVGGTLSNAGISGQTFRYGPQISNVYEIDVITGTADFVTCSPNNNSDLFYAALGGLGQFGIITRARIPLEPAPKRVKWVRMLYTDFLDFTRDQELLISKNGRNDNKALNYLEGSLLLDQGSLDNWRSSFFPPQDQPKIISLITKFRIVYCLEIVKHYDSQTKTTVDKDLQQLLKGLSYLPGFMFEKDAKYEEFLNRVHSEELKLKAKGLWDVPHPWLNLFIPKSKISDFNDGVFKSIVLKRNITTGPVLVYPMNRKKWDDRMSAVIPDEEIFYTVGLFQSSGFDDWRTFEDQNKEILQFCEKAGIKVKQYLPHYTTKVGWVNHFGSKWSTFQKRKLQFDPKLLLSPGQRIFNNNQ

>GbCKX13A

MIACLGRIVHDSDAESKLDDDVSTIFNSLNLQGSIENGDVSGIASKDFGGLYSVKPLYLIRPSGAEDISRVVKAAAGTPHLTVAARGNGHSINGQAMADGGYVIDMRSTGENHFKLLTVNGSPCIDVSGGALWEDVLRRCVSRFGLAPRSWTDYLSLTVGGTLSNAGVSGQAFRYGPQTSNVTELEVVTGKGDITVCSETQNPELFFGALGGLGQFGIMTRARVKLQLAPDMVRWIRVVYAEFEEFTRDAEFLVSQKEDESFDYVEGFVFCNNDDPVNGWPSVQLNPDQEFNPAHLPQTAGPVLYCLELAFHYRNSDLPSTVDMAVSRLVGGLGFVDGLISQVDVSYMGFLLRVKRAEQDAKANGVWDNPHPWLNLFVSKSDIVEFDQTVFKKMVKNGIGGPMLIYPLLRSKWDSRTSVALPEGEIFYIVALLRFVPKGPSVEKKVAENREIVKWCIKEGLDFKLYLPHYRAKEDWKRHFGNQWTRFEKRKANFDPMAILAPGQRIFKRTNQ

>GbCKX14A

MAFSSTMILPLLLVLLSAFSATSKSVQENFMQCLDANSKHPIPISAFCFQTNSSFTSVLNSTAQNLRYLMPLVPKPEFIFIPVYESHVKSAVICAKKLAIHLRFRSGGHDYEGLSYASEIETPFILLDLIQLRSINVDIDDNSAWVQAGATVGEVYYRISEKSKTHGFPAGLCSSLGIGGHITGGAYGSMMRKYGLGADNVLDARIVDVNGEILDRAAMGEDLFWAIRGGGGASFGVILSWKIKLVAVPETVTVFTVPKTLEQAATKILYRWQQVADKLDDDLFIRVVIQVTKTSQKGKRTVTTAYNALYLGDAERLLQVMDQSFPELGLARKDCIETSWIKSVLYIAGFPSETPPEVLLEGKSLFKNYFKAKSDFVQQPIPETALERLWEMLLEEESPLMIWNPYGGMMANISDSAIPFPHRRGNLFKIQYVTSWYEGSKGATRKHMDWIKGLYDYMSAYVPTSPRAAYVNYRDLDLGMNHKNASFTEASVWGAMYFKGNFRRLVKIKSKVDPGNFFRHEQSIPVVLE

>GbCKX15A

MAISWPLVVSVLLSISSLVTSASNSDSVHEAFVQCLLDNSHPSHPISEAIFTPQSPSYATVLQSYIRNLRFNETYTPKPFLILTALHQSHIQAAIICAKKGNIQMKIRSGGHDYDGLSYVATVPFFVLDMFNLRSIDIDTETETVWVQSGAILGELYYRISELSKTHGFPAGVCPTVGVGGHFTGGGYGNMMRKYGLTVDNIVDAYVIDVNGRIHDRKSMGEDLFWAIRGGGAASFGVVLAYKIKLVHVPEIVTVFRVEKTLEDNATDIVDQWQHVASKLPKELFVRLVIDVVNSSTRTGGSTVRVSFISLFLGDSKTLVSIMNENLPLLGLSQSDCIETSWIRSVLFWTNITIDSPTDVLLNRTPSLSYLKRKSDYVKQPIPKTALEGIWEKMIELQPAQMIFNPYGGRMAEIESTATPFPHRAGNLWKIQYLANWNQGGAETAQRYIGLTRKLHRYMTPFVSKNPREAFLNYRDIDLGVNHNDRGSYLEGRVYGIKYFKGNFNRLVHIKTKVDPTNFFRNEQSIPTLPH

>GbCKX16A

MPNPMRPYLLLSLVFFFNLYHSMAVSDPTHQALLQCLTQSIPTDTASSIIVSKSNPSYTSVLRAYIRNARFNTSSTPKPLIIITPLDESHVSAAVICSRKLGFQLKIRSGGHDYEGLSYVFDKPFFVLDMFNLRSITVNMADETAWVGAGATLGELYYNIWKNSKVHGFPAGVCPTVGVGGHLSGAGYGTLIRKYGLSVDHVVDAKLVDVKGKILDRKAMGEDLFWAIRGGGAASFGVVLSYKIKLVPVPETVTVFRIERLLTENATDITFKWQTIAPTTDENLFMRMLLQPVTRNKKKTARITVIALYLGDSDSLVSLLQKDFPELSIGKSNCNETTWIDSVLWWANFDLGTPPTALLDRDLNDAGFLKRKSDYVQTPIPKSGLESLWQKMIELGKVGMVFNAYGGRMDQIKPDETPFPHRAGNLYKIQYSVNWDEPGNEADKNFTTQAKLLHDFMTPFVSKNPRSAYFNYRDIDVGSTKKWSYEEGKVYGESYFNGNYERLVDVKTAVDPNNFFRNEQSIPPRSSKI

>GbCKX17A

MAVSFPIPSYFTAIFIISRLMSIMGISKHLNNKLLPPLDNITDKLSLDPSAIESASQDFGHIVKSIPKAVLQPSSIADIASLINFSYNSSIPFTIAAKGHGHSVRGQAMANDGVVVDMTSMKKHRNGTGIWVSNDGVYADVGGEQLWIDVLNATLKHGVAPVSWTDYLYLTVGGTLSNGGISGQSFRYGPQISNVYEMDVITGKAEIVTCSPNKNSELFHAALGGLGQFGIITRARIPLEPAPKRVKWIRMLYNDFTAFTRDQELLISINGRHDSHALGYLEGSLLMDHGSPDNWRSSFFPPKHHPKITSSIINHRIIYCLEVVKHYDYQTQNTVDKELEQLLKGLSYMPGFMFEKDVLYAEFLNRVQRGELKARSQGLWDVPHPWLNLFIPKSQIQRFNDGVFKGIVLERNITTGPVLVYPMNRKKWDDRMSAVIPDEEIFYTVGFLHSSGFDDREAFDDQNKEILKFCEEAGIGVKQYLPHFTSKDEWVHHFGSKWETFQQRKFQFDPKMILSPGQRIFNNN

>GbCKX18A

MYYFRWLLGFLHLLVWGSIILVHAIPPPDPVQCNRTICTLSNSYGAWGDRKDCSVKSVVYPTTEEELRSAVAHANKNKLKVKVVSQFSHTIPKLACPSSLGHHDSLLISTAKYDSSIEIDSVNLAVTADAGVALRDVIDKVEEAGLSLAAAPYWEGVSVAGMISTGAHGSSWWGKGGAVHDHVIGLSMIVPGNESEGYAKVKQIAAQDQLLNAAKVSLGILGVISKVKLSLERGFKRSITYNFTNDSSIENNFMEHGKKYEFGDITWYPSKHTAVYRYDSRVPMETPGDGINDFLGFQSNEILISKSVRASEKLFESTKSVNGECTLADTTLWYKKQIGNGLKNNGQIFTGYPVVGRQGKMQTSGSCLYSPKTRIDASCAWDPRIKGLFFYE

>GbCKX19A

MANLSLLLLHFLISSLSVSGSAATDQRNIMSCLNYYNISNYTISSNVHNHDYSILLNFSIQNLRFAEPTIPKPIAIILPENKEQLINTVVCCTIGPWEIRVRCGGHSYEGTSSVASDGAPFVIIDMMNLNRVSVDLGNETAWVEGGATLGETYHAIAESSDIHGFAAGSCPTVGTGGHIGGGGFGFLSRKYGLAADNVIDALLLNAEGELLDRQAMGEDVFWAIRGGGGGIWGIVYAWKIKLLKVPKTVTSFIVSRPGTIAHVANLVNKWQHVAPNLEGDMYLSCAVGAGLPQAKSIGISATFNGFFLGRKREAVLILGQVFAELDVSEEACKEMSWIESVLFFSGLGDGAIVSDLKNRYLHDKHYFKAKSDYVRNPISLTGIRTAIDILEKQPKGYIIMDPYGGIMNNISNDSIAFPHRYGNLYTIQYLVEWHQEEKNRSNEYREWIRDFYDAMASHVSWGPRAAYVNYMDFDLGVMELINTSVLSEDTVEMARVWGEKYFLNNYDRLVKAKTLIDPNNVFKNQQGIPPSTTTGLKARTF

>GbCKX20A

MVNLSLLLFLISSPSVSGSASPDQRNIMSCLNSYNISNYTISSNVHNHDYSILLNFSIQNLRFAEPTIPKPIAIILPENKEQLINTVVCCTKGPWEIRVRWVEGGATLGETYHAIADSSGIHGFSAGSCPTVGTGGHIGGGSFGFLSRKYGLAADNVIDALLLNAKGELLDRQAMGEDVFWAIRGGGGGIWGIVYAWKIKLLRVPKTVTSFIVSRPSTKAHVANLVNKWQHVAPNLEGDMYLSCAVGAGLPQAKSIGISATFNGFFLGRKRGAVLILGRVFAELGVAEEACKEMSWIESVLFFSGLGDGAIVSDLKNRYLHDKHYFKAKSDYVRNPISLTGIRTAIDILEKQPKGYIIMDPYGGIMNNISNDSIAFPHRYGNLNTIQYLVEWHQEEKNRSNEYREWIRDFYDAMASHVSWGPRAAYVNYMDFGLGVMELINTSVLSEDTVEMARVWGEKCFLNNYDRLVKAKTLIDPNNVFKNQQGIPPSTTTGLKARTF

>GbCKX21A

MRIAYLDRTVHETDGEPKPNGDVSAISKSIDLQGSVETGDKTTIASKDFGGLYSIKPLALIKPAGSDDISRAIKAASRIPRMTVAARGNGHSINGQAMTNGGLVIDMRSTEENHFRLLNINGSFFIDVSGGALWENVLTRCVSRFGLAPRSWTDYLSLTVGGTLSNAGVSGQAFRYGPQISNVTELEIVTGKGDITVCSETRNPELFFGSLGGLGQFGIITRARVKLQPAPDMVRWIRVVYTEFDEFTRDAEFLVSRDDGESFDYVEGFVFCNNDDPVNGWPAVPLDPVHGFNPGIIPQTGASVLYCLEVAFHYQKGDHPSTVDKAVAGLLKRLRFVEGLKSQVDLSYVEFLLRVKRAEEQAKANGIWDAPHPWLNLFISKSNIVDFDQTVFKKMVKDGIGGPMLIYPLMRSKWDNRTSVALPDSEIFYLVALLRFVSRGPSVEESVAQNREIVEWCIKEGLDFKLYLPHYQSKEGWKRHFGDQWTRFVERKASFDPMAILAPGQNIFKRTHLS

>GbCKX22A

MGSPVCGFLKQNNIIFLRFFAILVLSCIPDRTNLCSNPSFDTPTIPPHSSSSSIPSSLKTLTLDGYFSFENLKHAAKDFGNICHYLPIAVLHPKSVSDISSTIKHILYMSSVTKLTVAARGRGHSLQGQAQAYQGVVINMESLDRPSMYIENGEVPYVDVSGGELWINILHETLKYGLSPKSWTDYLHLTVGGTLSNAGISGQAFRHGPQINNVYQLEVVTGTGEVVTCSDKENADLFYGVLGGLGQFGIITRARISLGPAEKMVKWIRVLYSEFSTFSNDQEHLISSNNSFDYIEGFVMINRTGLLNNWRSSFNPKDPIQASQFSSDGKILYCLEMVKYFNPEKIDVLNQDIEKLLSELNYIPSTLFLSEVSYVEFLDRVHLSEIKLRSKGLREVPHPWLNLLIPKSRIFDFAEGVFGNIVKDNNNGPILIYPVNKAKWNNRTSMVTPEEDIIYLVAFLSSALPGTDGLEHIMTQNQHILDFCAKAQLGAKQYLPHYHTQDEWRAHFGTQWETFVQRKSAYDPLAILAPGQRIFQKAISIT

>GbCKX23A

MGFSFWLACLRSCSNKSAFSKAFRDSFYHYKSQLNTCQKNLPSTIAEKTNSHAFSWSSCLLPLAFAVSAGSLTFQSHNNHPSLCEPSNLDSRKVTIGGKASTEFVVKGTHKEVPQELIDELKAICQDNMTLDYDERFYHGKPQNSFHKAVNIPDVVVFPRSEEEVSQIVKSCNKHKVPIVPYGGATSIEGHTLSPNGGVCIDMTLMKRVKALHIRDMDVVVEPGIGWMELNEYLEPYGLFFPLDPGPGATIGGMCATRCSGSLAVRYGTMRDNVISLKVVLANGIIVKTASRARKSAAGYDLTRLMIGSEGTLGVVTEVTLRLQKIPEHSVVAMCNFPTIKDAADVAIDTMLSGIQVSRVELLDEVQVRAVNIANGKNLPEVPTLMFEFIGTEAYSHEQTQIVQRIVSEHNGSDFVFTEDPEAKKELWKIRKEALWACFAMEPNFEAMISDVCVPLSNLAELISRSKQELDASSLVCTVIAHAGDGNFHTVILFDPNEEEHRREAERLNQFMVYTALSMEGTCTGEHGVGTGKMKYLEKELGIEALQTMKRIKTALDPNNIMNPGKLIPPHVCF

>GbCKX24A

MANSDHLKQNKTILQCLTDHSIASPSISSVTFFPTDPSFTSTLQSYIRNLRFTSTTTPKPLFIVVPSRVSHIQASIICCKTHGLEMRIRSGGHDYDGLSYVSKAPFMILDLFNLRSVSVDNGTAWVESGATLGELFYAISQKSKTHGFPAGVCPTVGVGGHCSGGGYGNMMRKFGLSVDNVIDAKLVDVNGNVLDRESMGEDLFWAIKGGGGASFGIIISWKIKLVSVPEIVTVFKIEKSLEQGVTGIVHKWQYIADKIDPNLFVRVVLLPVNKKHLQSIKAKFIGLFLGNGQELISLMNETFPELGLSFDQCIEMSWIESILFWSNYPKGTSLDVLLDRQPQQEKYLKKKSDYVQEPISKENLEGIWNKMIELKRPALTLNPYGGKMSEISEFETPFPHRAGNIYKIQYSVTWKDDGVEASDRSLDQIRKLYDYMTPYVSKSPRSSYLNYRDVDIGINENGNASYSEGVIWGRKYFKGNFQRLVQVKSKVDPGNFFRYEQSIPCLGSWKSITAE

>GbCKX25A

MLEDKHKMLNPKCVHFLTLLISLLSLPSPTISQSSSLTNFLHCLHYGSDPMVSQSIYIASNPAFQTILQARIKNRRFLNPETLKPVAIVVPTHIDHVQGTVICAKDNGLQIRIRSGGHDYEGLSYRSNVTFIILDMSNFRSIDIDIKTETAWVQSGATLGELYYHIANKTNTHGFPSGVCPTVGIGGHFSGGGYGNLMRKYGLSVDNILDIIAVDALGNVHDRASMGEDLFWAIRGGGAASFAVVVSYKIKLVRVPNKVTVFTKGFTLEQGATDLVHKWQQIAPNINEELFIRVKLQPSFINGNQTVTATFIGFFLGRREKLLPIISKTFPELNLTQQDCHEMRWVETTLFWAGFPIGTPIETLLNRTIWTPLFFKNKSDYVKNVIPKESLNKIWKMTMAMMDRNDINKTRFDLECSPYGGKMNVISESNTPFPHRKGNLFLIQYSFSWIDEGNNVSFNNIEKLRKLYDGMAPYVSKDPRECFLNYRDLDIGSSRSNETSFDDAKIYGRKYFKDNYTRLTKVKASVDPNDFFKYEQSIPPIN

>GbCKX26A

MKISSSIIPFISIFILLSISSVTPSDSDDFDDFLRCLPKQSDSSIPIANAILTPNSSSFQYIYQVRASNLRPLLSATSRPVAIITALHPSHAQASVICAKRHDFQLRIRSGGHDFEGLSYTSDVPFVILDMFNLNSIDIDMSTETAWVQAGATTGELYYRIAEKSNVHGFPSGVCTTLGIGGHFSGGGYGFLMRKYGVSIDNVIDAQLIDANGRILNRKSMGEDVFWAIRGGGTTSFGIILSWRIKLVRVPPRVTVFTVQRTLEQGATELAYRWQQVAPKLPKDLFIRLQPEPINNGGNNKTVRVSFIGHFLGQADGLLRLMNVSFPELGLTRNDCLEMSWVESTLYWAGFSNGTSIDVLLNRVAVNKVFAKDKSDYYKAVIPKQGLETLWQVLMDIENIFVQMNPYGGRMEEISDSETAFAHRAGNLFKVLYGIQWSESEGGVNATARYVELSRRLYNAMAPYASSNPREAFINYRDLDIGSNESEKTDFEDAKEYGAKYFRNNFIRLAGVKAKIDPENFFKNEQSIPPLPSH

>GbCKX27A

MKISSSIFPLVSIFSLLLISSATSSDFDDFIRCLPQQSNSSVPITDAILTPNNSTFQSIYQLRANNLRTFLSATSRPVAIITALHPSHAQAAVICAKRHDFQLRIRSGGHDFEGLSYTSDVPFVILDMFNLNSIDIDVSTETAWVQAGATTGELYYRIAEKSNVHGFPSGVCTTLGIGGHFSGGGYGFLMRKYGVSIDNVIDAQLIDANGRILNRKSMGKDVFWAIRGGGTTSFGIILSWRIKLVRVPPRVTVFTVQRTLEQGATELAYRWQQVAPKLPKDLFIRLQPEPINNGGNNKTVRVSFIGHFLGQADGLLRLMNVSFPELGLTRNDCLEMSWVESTLYWANFPNGTSIDVLLDRVQENRVFSKSKSDYYKALIPKQGLETLWQGLMDIEDIFVQMNPYGGRMEEISDSETAFAHRAGNLFMVLYGVQWSESEGGINATERYVEMSRRLYEAMARYASSNPREAFFNYRDLDIGSNESDETDFEDAQEYGAKYFRNNFIRLAKAKATIDPENFFKNEQSIPPLPH

>GbCKX28A

MKASSYTCFIVSIFVLLSISSAASYNPVDFDAFLQCLPKHSDHSVSIVGAILTPNNASFRSIYQLRANNLRILSSATSRPVAIITALHPSHAQAAVICAKRHGFQLRIRSGGHDYEGLSYISDVPFVILDMFNLNSIAIDMKTETAWVQAGATTGELYYSIAQKSEVHGFPSGVCTTLGIGGHFSGGGYGFLIRKYGLSIDNVIDAQLIDAKGQILDRKSMGEDVFWAIRGGGTTSFGIILSWRIKLVRVPPRVTVFNVQRTLEQGATELAYRWQQVAPKLPKDLFIRLQPVPINNGGNNKTVRVSFIGHFLGQADGLLRLMNVRFSELGLTRNDCLEMSWVKSSLYWAGFPNGTSIDVLLDRVQVNRVFYKSKSDYYKAVIPKQGLETLWQVLMDIEDIFVQFNPYGGRMEEISESETAFAHRSGNLFIVLYRIQWSQSDGTGRYVEMSRRLYNAMAPYASSNPREAFFNYRDLDVGSNESGETDFEVAKEYGAKYFGNNFMRLAGVKAMVDPENFFKNEQSIPPLPTPPSH

>GbCKX29A

MKISSSIFSLISIFILLLISSATSSDFDGFLQCLLQQSNSSLPIIDAILTPNNSTFQSIYQLRANNLRTFLSAISRPVAIITALHPSHAQAAVICAKRHDFQLRIRSGGHDFEGLSYTSKVPFVILDMFNLNSIDIDMSTETAWVQAGATTGELYYRIAEKSNVHGFPSGVWTTLGIGGHFSGGGYGFLMRKYELSIDNVIDAQLIDANGRILNRKSMGEDVFWAIRGGGITSFGIILSWRIKLVRVPPRVTVFTVQRTLEQGATELAYRWQQVAPKLPKDLFIRLQPVPINNGGNNNTVRVSFIGHFLGQADGLLRLMNVSFPELGLTRNHCLEMSWVESTVYCANFPNGTSIDMLLDRVQENKVFSKSKSDYYKALIPKQGLETLWQGLMDIEDILVQMNPYGGRMEEILDSETAFAHRAGNLFMVLYRVQWSESHGGINTTERYVEMSRRLYEAMAPYTSSNPREAFLNYRDLDIGSNESDETDFEDAQEYGAKYFRNNFIRLAKAKATIDPENFFKNEQSIPPLPH

>GbCKX30A

MKASSYTCFIVSIFVLFSISSAASYDPVDFDAFLQCLPKHSDHSVSIAGAILTPNNASFQSIYQLRANNLRVLLSATSRPVAIITALHPSHAQAAVICAKRHGFQLRIRSGGHDYEGLSYISDVPFVILDMFNLKSIDIDMKTETAWVQAGATTGELYYSIAKTSEVHGFPSGVCTTLGIGGHFSGGGYGFLIRKYGLSIDNVIDAQLIDAKGRILDRKSMGEDVFWAIRGGGTTSFGIILSWRIKLVRVPPRVTVFNVQRTLEQGATELAYRWQQVAPKLPKDLFIRLQPVPINNGGNNKTVSVSFIGHFLGQADELLRLMNVRFPELGLTRNDCSEMSWVESALSWAGFPNGTSIDVLLNRVQVDRVFYKSKSDYYKAVIPKQGLETLWQVLMDIEDIFVQFNPYGGRMEEISESETAFAHRGGNLFKAQYGIQWSESDGGINATGRYVELSRRLYNAMAPYASSNPREAFFNYRDLDVGSNESGETDFEVAKEYGAKYFRNNFMRLTGVKAKIDPENFFKNEQSIPPLPTPPSH

>GbCKX31A

MTPPLSTPSLLPLLLVAFNICFSLAASNSIYESFVQCLKTRSNSSDNISDIVYSHSNATYETVLEQYIRNARFNTSSTPKPVIIITPLTESHVSAAVICSNNIGFQLRIRSGGHDFEGVSYVSDQPFFILDMFNLRSISINMADQSVWVQSGATLGELYYRIWEKSRVYGFPAGVCPTVGVGGHISGAGYGNMVRKYGLSVDYVVDAKIVDVNGNILDRKAMGEDLFWAIRGGGGASFGVILAFNIKLVEVPEMVTVFKLERTLEQNATDVVYKWQSVAPTTDDNLFMRMLVQPVTLNKQKTIKISIMALYLGDVNSVVPLLAEDFPELGLVTEDCFEMSWIESALWWASFGKGTSPTVLLDRESYHVKFMKRKSDYVKTPISKDGLQWLRKKMIELEEPGLVFNPYGGKMNEIKETETPFPHRAGNLFKIQYSINWKDMGIEADKRSRNLVNRLHSYMTSFVSKNRRSAYLNYRDLDIGITKNWSYQEGKVYGESYFNGNFERLVDVKTAVDPPNFFRNEQSIPPRTIKAWNEKNEGSIPPSTSKAWNKSKPYVMIILFMAIGII

>GbCKX32A

MAFTSISSLFLLLLVLHFSSSTTASTCHAKSFKLNPIQEKFIQCFKANSEIPIPVSTEFFTPNNASFSTVLQSTAQNLRYLEPPVPKPEFIIMPLNESHVQAAVICSKELGIHMRVRSGGHDYEGMSYVSETESPFILVDLSKLRSVKVDIENNSAWIEAGATIGEVYYRIYEKSKIHGFPAGLCTSLGVGGHITGGAYGSMMRKYGLGVDNVIDARIVDVNGRVLDRAAMGEDLFWAIRGGAGGSFGIILQWKIQLVPVPSTVTVFTITKSLQQNGTKIFHRWIEVADNLDDDLFIRVIIQTAMINGEKTVTTSYNSLFLGEADRLVEIMQQSFPELGLTRKDCIETSWIKSVLYIAGYPSNTPPEVLLQGKSTFKNYFKAKSDFVKSNIPETALEGLWKRFMEEDTPLMIWNPYGGMMARISESETPFPHRKGNKIMIQYVSAWQDGDKNESKHIDWIRRLYNYMAPYVSMFPRTAYVNYRDLDLGTNKNASTSFIEASGWGVKYFKDNFNKLVKVKTKVDPENFFRHEQSIPPLPVEARF

>GbCKX33A

MEKWRATSNLRRSLKSILNRQLSSVSEFRYLNEKRSCQSSFNLIRDCKSLGQVNAIQHRCFSSASTLVQRNPSFSTLNSDDISYFKGLLGEKSVIQDEDRLETVNTDWMHKYKGSSKLLLLPRSTEEVAQILRYCNSRCLAVVPQGGNTGLVGGSVPVFDEVIVNVSSMKNIISFDKVSGILVCEAGCILENLISFLDNQGFIMPLDLGAKGSCQIGGNVSTNAGGLRLVRYGSLHENVLGLEAVLANGDVLDMLGTLRKDNTGYDLKHLFIGSEGSLGIVTKVSILTPPKLSSVNIAFLACNDYSSCQKLLMEAKRKLGEILSAFEFLDTEAMNLVLHQFDGVRNPLPASMHNFYILIETTGSDESYNREKLEAFLLSSMEDGLISDGVLAQDINQASSFWRIREGVPEALMKAGAVYKYDLSLPVEKMYDLVDDMRIRLGDLAKVVGYGHLGDGNLHLNVSAPEYDDKILEQIEPYVYEWTSKHRGSISAEHGLGLMKANKIYYSKSAETVQTMASIKKLLDPNGILNPYKVLPHSLNS

>GbCKX34A

MSFFFPEMKASQCFSMSLFLLILLISCPWLISANPHLNNFLGCLDSFYSNDISKVIYTQNNASYSSVLNATIQNLRFSTPATPKPLVIVTPLQTSHIQATIRCSRTNGLNLRIRSGGHDFEGLSYVSQVPFVVLDLTNFRSVKIDVKNKVAWVQSGAILGEFYSEIARRSRTLAFPAGICHTVGVGGYLSGGGYGLLLRKYGLAVDNVIDAVFIDVNGRILKRKSMGEDLFWAIRGGGGGSFGVVLSWKVKLVSVPSTVTVFTIRKTLEENATNLVHQWQSVAHKLPGDIFSAVTMRKVNRSGKPTILVAFSSFFLGDTNALIPLMKARFPELGLKKEHCTEMSWIESILYFGQIQNKSIDVLLDRSYKSPLNAPSFKTKLDYVKNPIPKAGFEKIWSKLYEEDAETAAMAFIAYGGKMAEIPESATPFPHRDGNLYHIAYTVGWDGEENTKSQRYMNWIRKFYSFMTPFVSKSPREAYVSYRDDDIGTNNKKGETSYAKASVWGRKYFKNNFDKLIYIKTKVDPHNFFKHEQSIPVSV

>GbCKX35A

MGKLKAVAVVTMISTVLLSILWRATLHLEDNENFVRCLLDHSHPSHPISSAIYTPKSSSFSSVLESYIRNLRFNESSTPKPFLILTALHEFHIQAAVTCGKSHGVQMKIRSGGHDYEGLSYVSTLPFFLLDMFNLRSIDVDIETETAWVQTGATLGEVFYRIAEKSKTHGFPAGVCPTVGVGGHISGAGYGNMMRKYGVSADNVLDALIIDANGRLLDRQSMGEDLFWAIRGGGGASFAVVLAYKIKLVRVPETVTVFQVDRTLEEDATDIVDQWQHVAYNLPQELFIRLMLDVVVKSSGEKTLRASFVSLFLGDSESLLSIMKERFPKLGLSKSDCIETSWVKSVLFWSNIPLETDIQVLLDRTPQTLDYLKRKSDYVREPIPKAGLESLWKKMMELEKPRMYFNPYGGKMAEIAAEEIPFPHRAGNLWKIQYLANWNEAGIEAANRYIDLTRKLHEFMTPFVSKNPRQAFLNYRDADLGSSSHGKASYSAARLNGMKWFMGNFERLVQIKTEVDPTNFFSYEQSIPLLPQQVHLDDDI

>GbCKX36A

MQKHKMKSNIPIPLLFLSILFSFSWAALGLTHQQQHHSFLQCLNRHSGDANSISTVIYTQTNSSFSSVLEFSLRNARFSTPNTLKPLVIVTPSHVSHIQATINCSRTHGLQIRIRSGGHDYEGLSYVSQVPFVIIDLINLRSIDVDAENKTAWIQAGATIGELYYRIAEKSSTLAFPAGICPTVGVGGHFSGGGYGMLMRKYGLAADQIIDAQLVDVNGKFLDKNSMGEDLFWAIRGGGAASFGVVVAWKVKLVPVPSTLTVFTVNRTLKENGTMLVHKWQSVAPKIHEDLYIRLFLRAVNSSQQEGKRTIQASFVSLYLGRADELVDLMQESFPELGIVKEDCIEMSWIQSIMYFPSDIPEDAPLEILLNRTGSAGIFKGKSDYVTQTIPETALEGLWQRFYEDETESLEILFSPYGGNMDDIPETETPYSHRAGILFNIHYVVGWSEEDASESQRYINFMRRLYRYMEPYVSKSPRRAYMNYRDLDLGTNNNGPYTSYKQASKWGLPYFDKNFNRLIHVKTLVDPTNFFRHQQSIPSLSRG

>GbCKX37A

MLVSFPGQNNMLVLRSFMILFFSCITIKINLCFPSIPSSLKTLSIDGHFDFEQVEHAAKDFGNRYSYIPLAVLYPKSVSDIATTVNHVWQIGGGSELKIAARGHGHSLQGQAQAHRGVVINMESLQGHKMQVHTGNFPYVDVSGGELWINILRESLKHGLAPKSWTDYLHLTVGGTLSNAGISGQAFRHGPQISNVYQLEVVTGKGEVVNCSEKQNSDLFHSVLGGLGQFGIITRARISLEPAPEMVKWIRMVYTDFATFTRDQEKLISGKSTFDYVEGFVIINRTGLLNNWRSSFNPQDPLQASQFKSDGRTLFCLELAKYFNHEDMVLVNQEIKTSLSQLNHIPSTLFISEVPYMEFLDRVHISEIKLRSRGLWEVPHPWLNLFIPKSKIHSFAQEVFGNILTDTSSGPILIYPVNKSKWDNRTSVVIPEEDVFYLVAFLSSAVPSSTGNDGLEQILIRNKRILDFCEIAGLGVKQYLPHFSTQGEWKSHFGPHWEAFTRRKSTYDPSAILAPGQRIFQKLIAYS

>GbCKX38A

MKFLQFSMLLLPILLDALLSFSMGTSYHEDFLQCLSLRSNDSTSISNVVYTRNNSSYSSVLESTIRNLRFNSTDTPKPLVIVTPSRTSHFQATIYCARKHRLQIRTRSGGHDYEGHSYVAKVPFVLVDLVNFRSVDVDAENRVAWVQAGAIVGEVYYRIAKKNRTLAFPGGVYYTIGLSNFISNRGFGLLFRKYDTGGDNVIDAQLIDVNRRILTGNLWGKICFGQFEVVVVEASGSSWHGS

>GbCKX39A

MKFLQFSMLLLPILLDALLSFSMGASTHQDFLQCLSLRSNDSTSISNVIYTRNNSSYSFVLESTIRNLRFNSTDTPKPLVIITPSRTSHFQATIYCARKHGLQIRTRSGGHDYEGLSYVARVPFVVVDLVNFRSVDVDVENRVAWVQAGAILGEVYYRIAEKSRTLGFAGGIYFTVGVGGHISGGGFGLLFRKYGLACDNVIDAQFIDVNGRILDRKSMGKDLFWAIRGGGGGSFGIVLAWKVALVPVPPTVTAFSISRTLEQNATQLILRWQDIAHQLPDEMNPDVTMFSFNSTQDGRKTILVSFSSLFLGTIDELLPIMQQRFPELGLSGQDCIEMSWIEAVLYYNQLQNQPLETLLNRTFRTPSGGQYYKIKSDYVKEPISETGLNGLFSRLSDEEASSAVIIFMAYGGIMGRIPEDATPYAHRAGNLFKIYYNVNWQEQDNVNSQKYIDWSRRVYKDMTPFVSKSPREAYANYRDLDLGSNNVGITSYTQASIWGRKYFKNNFDRLVQVKTKIDPENFFKHEQSIPPLF

>GbCKX40A

MKSLQFSMLLLSLVLAALLSFSTGALPQEDFLRCLSLRSNDSSTISSVIYTRNNPSYSTVLESTIRNLRFNSTNTPKPLVIVTPSRTSHFQATIYCSRKHGLQIRTRSGGHDYEGLSYVAKVPFVVVDLVNFRTVDVDVENRVAWVQAGAILGEIYYRIAEKSRTLAFAGGIFHSIGVGGYISGGGFGLLFRKYGTAGDNVIDAQFIDVNGRILDRKSMGEDLFWAIRGGGGGSFGIVLAWKLKLVPVPAIVTVFSVNRTLEQNATQLILRWQEIAHQLPDEMNPDVTMLSVNSTQDGRKTILASFSSLFLGTIDELLPIMQQRFPELGLSRQDCSEMSWIESVLYFNQLQNQPLEILLNRTFRTSVGGQYYKIKSDYVKEPISETALNGLFSRLSDEEASSAFIIFMAYGGIMDRIPEDATPFPHRAGNLYKIYYDVNWQEQDNVNSQKYIDWSRRVYNYMTPFVSKSPREAYANYRDLDIGSNNVGITSYRQASVWGRKYFKNNFDRLVQVKTKIDPQNFFKHEQSIPPLH

>GbCKX41A

MFRSLSLKRSFKTHLKTLIHCPQQPPPPPPPSPVLFSSIRALSTASSPPSSSSDSELRKYLGYTALLAFCGVATYYSFPFPENAKHKKAQLFRYSPLPEDLHTVSNWSGTHEVQTRHFHQPENLKQLEELVKESNEKRVKLRPVGSGLSPNGIGLARGGMVNLALMDKVLEVDKEKKRVRVQAGIRVQQLVDEIKDYGITLQNFASIREQQIGGILQVGAHGTGAKLPPIDEQIISMKLVTPAKGTIELSKEKDPELFYLARCGLGGLGVVAEVTIQCVERQELVEHTTVSNLKDLKKNHKKMLSENKHVKYLYIPYTDTVVVVTCNPVSKWRGPPKFKPKHTTDEAMQDIRELYKESLKKYRARDIKTKSSDSDEPNINEFSFTELRDKLLSLDPLNKDHVMKVNHAEAEFWRKSEGYRVGWSDDILGFDCGGQQWVSETCFPAGTLSKPSMKDLEYIEELKKLIETNELPAPAPIEQRWTARSQSPMSPASSSAEDDIFSWVGIIMYLPTMDARQRKEITEEFFHYRHLTQSQLWDKYSAYEHWAKIEVPKDKEELEALQARLKARFPVDAYNKARRELDPNRILSNNILEKLFPLSDTV

>GbCKX42A

MPIIPVLYSNKFNELVLLKCLFLLVALVGCSPPGEPVKCSTKDSNCTVTNSYGAFPDRTVCRAGNVVYPISEQESVSILSAATEVQRKIKVVTHFSHSIPKLVCPDGQDGLLISTKNLNRVVKINLTAMTMTVESGVTLRQLINEAAKAGLALPYAPYWWGLTIGGLLGTGAHGSSLWEKGSSVHDYVVEMRIVSPAKAEDGYAKVWVLNESDKDLNAAKFSLGVLGVISQVTLKLQSLFKRSITYVMKDDTDLGDKVVTFGKLHEFADIFWYPSQRKVAYRIDDRVPVNVSGSGVYYFTPFRSTLSSVLSVIRSVEETQESLRDTEGKCLNAKLVTSTLQSSAYGLTDNGVIFTGYPVIGFHHRLQSSGTCLDSHKDSLITACPWDPRIKGEFFHQTTFSIDLSVVKSFIQDVQKLVSMDPKSLCGLELYNGIVMRYVKASTAYLGKQEDAIAFDITYYRSKDPMAPRLYQDVLEEIEQMALFKYNALPHWGKNRNLVFDGVMKRYKNGGEFLKVKNKYDPWGLFSSEWTDQVLGLRNGVTVLKEGCALEGVCVCSQDVHCAPRKGYLCKPGKIFLDARVCARVNTKN

>GbCKX43A

MPAIPMLFSNKFNVLLLWKCLCLLVVLVGCNPPGEPVKCSTKDYNCTVTNSYGMFPDRAICRAGNVAYPTSEQELVSIVSAATKSKRKMKVVTHFSHSIPKLVCPDGQDGLLVSTKNLNRVLKTNTEAMTMTVESGVTLRQLITEAAKAGFALPYAPYWWGLTIGGLLGTGAHGSSLWGKGSSVHDYVVEMRIVSPAKAEDGYAKVWVLNERDKDLDAAKVSLGVLGVISQVTFKLQPLFKRSITYVRKDDTDLGDEAVTFGKLHEFADIFWYPSQRKAIYRIDDRVPINVSGNGVYNFTPFRSTLSLVLALVRSSEETQESSGDAEGKCLNAKLMTSTLQSSAYGLTNNGAIFTGYPVIGFHDRLQSSGTCLDSLEDSLITACPWDPRIKGEFFHQTTFSIGLSVVKSFIQDVQKLVSMDPKSLCGLELYNGILMRYVKASTAYLGKQEDAIDFDITYYRSKDPMAPRLYQDVLEEIEQMALFKYNALPHWGKNRNLLFDGVMKRYKNGGEFLKVKNKYDPWGLFSSEWTDQVLGLRNGVTILKEGCALEGLCVCSQDVHCAPSKGYLCKFGKIFSDARVCARVNTKT

>GbCKX44A

MTLPTNLSIINNVIACNTFQCSHNIDPEIKLNMFPFLLIIILSLSWRISASAQPAEEFLRCLSIRFHNSSSIAKLVYTHHNSSYFSVLKSSAQNFRFTTPSTSTPLAIVTPFHASHIQATVYCCRKHGLQVRTRSGGHDFEGLSYTTAYKVPFVVIDLVNLRSVQVNVEKATAWVESGATVGELYYEIARKSRTLAFPAGIGHTVGIGGQLSGGGLGSLFRKYGLASDNVIDARLIDAYGRILDKKLMGEDLFWAIRGGGGGSFGIVLAWKLKLVPVPANVTACTVSKTLEQNATKLVHQWQSIARKFPKEIQSSIAIARVNSSEDGKMTIQASYGSVFLGSIDELIPLMEEKFPELGLVKEDCLEMTWAESVLYSALSIIGLPLETLLNRTQKSALSQTFFKAKSDFVKQPISESGFEGLWPKFYEDEAKSAVMVLVAYGGKMDEIPETEYPYPHRAGNLYSILYVVNWEEEENKNSEKFMNWMRRVYSYMTPYVSKSPREAYVNYRDLDIGTNKVNDEKGSYTEGKVWGLKYFKNNFDRLVYVKTKVDPQNFFSHEQSIPPLS

>GbCKX45A

MQRNMLFLRSFMILVLSCIAIKINLCFPNILSSLKTLPIDGHFNFEQLHHAAKDFGNRYSFLPLAVLHPNSVSDIATTVKHIWQMGPGSDLTVAARGHGHSLQGQSQAHGGIVINMKSLQGLKMQFHIGNLPYVDVSGGELWINILREGLKHGLAPKSWTDYLHLTVGGTLSNAGISGQAFRHGPQISNVHQLEVVTGKGEVVTCSEKQNSDLFHGVLGGLGQFGIITRARISLEPAPEMVKWIRVLYTDFATFTRDQEELTSGESTFDYVEGFVIINRTGLLNNWRLSFNPQDPVQASKFKSDGRTLFCLELAKYFNRDETAVVNREIHSSLSQLNHIPSTLFVSEVPYIEFLDRVHISEIKLRSKGLWEVPHPWLNLLVPRSKIQTFAQQVFGNILTDTSNGPILIYPVNKSKWDNRTSVVTPDEDVFYLVAFLSSAVPSSTGNDGLDHILIQNKRILEFCEIARLGVKQYLPHYSTQGEWKAHFGSRWEVFVRRKSSYDPLAILAPGQRIFQKAVPYSQ

>GbCKX46A

MSDLQAPLRPKRKKGLVDFLVQFRWVFVIFFVLPFSALYYFLIYLGDVRSEMKSYKQRQKEHDENVKKVVKRLKQRNPKKDGLVCTARKPWIAVGMRNVDYKRARHYEVDLSAFRNILEIDKERMIARVEPLVNMGQITRVTVPMNLSLAVVAELDDLTVGGLINGYGIEGSSHIYGLFSDTVVAYEIVLADGRVVRATKDNEYSDLFYAIPWSQGTLGFLVAAEIKLIPVKEYMRLTYTPVVGNLQDLAQGYMDSFAPRDGDQDNPEKVPDFVEGMVYSPTEGVFMTGRYASKEEAKKKGNKINNVGWWFKPWFYQHAQTALKKGEFVEYIPTREYYHRHTRCLYWEGKLILPFGDQWWFRFLLGWLMPPKVSLLKATQGESIRNYYHEMHVIQDMLVPLYKVGDALEWVHHEMEIYPIWLCPHRLFKLPVKTMVYPEPGFEQQHRQGDTPYAQMFTDVGVYYAPGPVLRGEVFDGAEAVRKMEQWLIKNHSFQPQYAVSELNEKDFWRMFDADLYEHVRRKYGAVGTFMSVYYKSKKGRKTEKEVQEAEQAHLETAYAEAD

>GbCKX47A

MIVCLGRIVQDTDADSIPDDNVSTLSESLDLQGTIESGGITGVAGKDFGGLYSVKPLALIKPSGTEDIARVVKATSQTSHLTVAARGNGHSINGQAMADGGFVIDMRSTEENHFKPLTIDGSHYIDVSGGALWEDVLRRCVSMFRLAPRSWTDYLSLTVGGTLSNAGVSGQAFRFGPQTSNVTELEVVTGKGEIMVCSATQNSELFFGALGGLGQLGIITRARVKLQPAPDMVRWLRVVYTEFEEFTRDAEFLVTQEEGESFDYVEGFVFSNSDDPINGWPSVPLDPDHEFNPAYIPQTASSVLYCLEVALHYRNSDRPSTVDTAVSRLLERLGFIQRLKFQLNVSYVEFLLRVKQVEEHAKANGNWDSPHPWLNIFISKSSIVDFDRTVFRKMLKDGVGGPMLIYPLLRSKWDSRTSVVLPEGEIFYIVALLRFVPKGPTVEKLVAQNHEIIKWCNKEGLDFKLYLPHYQSKEDWKRHFGNQWTRFVERKTSFDPMAILAPGQNFFKRTHTIKP

>GbCKX48A

MVMSFQFPAYFTAIFIITRVMSIMKISKPLDVHHKDIRAVDLATKLSVDPSAIESASRDFGGIVKAEPEAVLHPSAPQDIAALIKFSYSNSVPFGIAAKGHGHSVRGQAMAENGVVVDMRSMANKRRNGTGIRVSIDRLYADVGGEQLWIDVLNATLEYGLAPISWTDYLYLTVGGTLSNAGISGQTFRYGPQISNVLEMDVITGKADFLTCSPRMNSELFYAVLGGLGQFGIITRARIPLQPAPKRVKWVRLLYDDFSSFTKDQELLISKNGRKDKSALDYLEGSLLMDQGSPDNWRSSFFPYKDHPKIISLITKHGIIYCLEIVKHYDDRTKHTVDKEMEQVLQGMNYMPGFIFGKDVGYEEFLNRVRSGELKLKSQGLWDVPHPWLNLFIPKSQISDFNNGVFRGIVLERNITTGPVLVYPMNRQKWDDRMSAVIPDEEIFYTVGFLHSSGFDTWKAFEDQNKDIMRFCNKTGIMLKQYLPHYSTKEEWVHHFGSKWKVFQHRKYQFDPRMLLSPGQRIFNNN

>GbCKX49D

MATMLLLTFLISSLIMVTVGLAIDSMEILRLGIDGQLSVDPSDVETASLDFGLLTRGQPLAVLHPVSAQDISQVVKAVYGSNQGMTVSARGHGHSINGQAQTTNGVVIQMSGWKGGNKPPRPRVWAEERYVDVWGGELWIDVLKSTLEYGLAPKSWTDYLYLSVGGTLSNAGISGQAFNHGPQISNVYELDVVTGKGEVLTCSEDENTELFHAVLGGLGQFGIITRARISLEPAPKMVRWIRVLYSNFSAFTSDQEHLISLHAEPSNQKFDYVEGFVIVDEGLINNWRSSLLSPQNPVKISTLFPSGGVLYCLEIAKNYHESTVQTIDQEVEFLLKKLNFISTSVFTTDLLYVDFLDRVHKAELKLRSKGLWEVPHPWLNLFIPSSKIAEFDKGVFKGILGNKTSGPILIYPMNKNKWDHRSSVVTPDEGVFYLVALLRSALDNGEETQSLEYLNNQNRQILRYCDEAGIKVKRYLPHYTTQQEWMDHFGNKWDRFYEMKMEFDPRHILASGQRIFTPTFPSSSNMPS

>GbCKX50D

MKSLHDCLPLLFVVVFSLSWVNASANSNDYFLDCLSSYHPDEFTSFSKVIYTETNSSYSAVLESSTRNPRFSTPNTPKPLVIVTPSNISHVQATIHCSKKHGLQIRTRSGGHDFEGLSYVSQVPFVVIDLVHFRSIDVDVKKEEAWIQSGAITGEVYYRINERTTNLTFPGALCHTVGIGGFISGGGYGFLFRKYGLAADNVIDAQFVDANGRVLHRRLMGEDLFWAIRGGGGGSFGIVLSWKVKLVHVPSTVTVFSVGRTLEQNATQLLHRWQYVAPNLPNDVYSLVSISSMNSTENGERTVLATFTSVFQGVADELIPLMQERFPELGLLKEDYIEMTWIESILFWNQLSNETSEILLDRSNRNSLVPLSYKSKSDYVRKPMPEIALQGLWSRLLEVNETSTAVVNIISYGGKMDEIPETETPFPHRKGTLYKINYNIVWQEEENSNPQRYISWMRTLYSYMGPFVSKSPRAAYVNYRDLDIGRNNDDGKTRYKQASVWGRKYFKNNFDRLVYVKTKIDPENFFKHEQSIPPRFH

>GbCKX51D

MKFLHRSLSLLFIVVCSLSWVTASANSHDDFLECLYSYHPNESSSITQVIYTETNSSYLALLNSSIRNQRFSTPNTPKPLVIVTPLNISHVQATIYCSKKHGLEIRTRSGGHDFEGLSYVSHVPFVVIDLVNLRSVDVDVENEEAWVQSGATVGEVYYRINERSTNLSFPAPVFRTVGIGGSISGGGDGSLFRKYGLSADNVIDAQLVDAHGRVLDRRSMGEDLFWAIRGGGGGSFGIVISWKIKLVHVPSTVTFCSVGRTLEQNATQLLHRWQYVAPNLPKDVYSVVTISTTNASENGTKTVLATFISLFQGGANEFSLLMQERFPELGLVKEDFIEMTWVESLLLMNGLSNETSEILLDRSNRYTILPPFVKSKSDYVREPMPEIALQGLWLHLLEVDEGGLAVQNFFAYGGIMEEISETETPFPHRKGTLYKISYNIAWQEEENNNSQRYISWMRKLYSYMGPFVSKSPREAYVNYRDLDIGRNNYHGKTSYKQASIWGRKYFKNNFDRLVYVKTKTDPKNFFKHEQSIPPCLHCTS

>GbCKX52D

MLCLYIRDLLVFLCTSSVLAIELLFLLRELRAGDLHVMKDKIMLVLLEVSIKVWISVAGILYKDMLRVKALHIRDMDVVVEPGIGWMELNEYLEPYGLFFPLDPGPGATIGGMCATRCSGSLAMM

>GbCKX53D

MKDSNSATFQLVTIVLFLSLSWRATSDSSSQVDKFLQCLANNSALMLESIYTPSNSSFENALQAYIRNRRFLTPETPKSVAIVAPNHVSHVQATVICAKDNGVQIRVRSVGHDYEGLSYRSNVTFVILDMFNLRSIDVDVDNEVAFVQAGATIGKLYYKIANESKGHAYPAGVCPSLGTGGHFSGGGYGNMMRKYGLSVDNILDAQVVGADGNILDRASMGEDLFWAIRGGGGASFAVIVSWKIKLVQVPEKVTVFTVPFTLEQGATDVAFKWQQVAPKLPEDLYIRQIASVINGSCPLEKKILVSFMGLFLGEADKLVPLVNQSFPELNLTKEDCKEMSWLESTVYWAGFPVGTPVQVLLNRTQGAPNIFKVKSDVKTVIPKEGLETIWQMLIGMGNTSAANISMQWNPYGGKMSEIPESSTAFPHRSGNLFLIQYVVLVRRGAQHPREAFLNYRDIDIGSSPSDQTNFTHAQVYGSKYFKDNFQRLTMVKAEMDPSNFFKNEQSIPPNFNFK

>GbCKX54D

MAFWRISICSLLLLLLSSSTMATASIYRPRKRPTSSLIQEKFIQCFTGSSQFYIPLSTAFFTPNNASFTSVLQSTAQNLRYLVPSMPKPEFIITPQHESQVQASVICAKRLDIHLRFRSGGHDYEGLSYVSQIESPFVIVDLSKLRSIKVDIQDNSAWVEAGATIGEVYYRIAEKSNIHGFPAGLCTSLGIGGHITGGAYGSMMRKFGLGADNVIDARIVDVNGRVLDRAAMGEDLFWAIRGGGGASFGIILEWKIKLVPVPATVTVFTVTKSLEQGATKLLYKWQTVADKLDEDLFIRVIIQKANAGKNNAKTVTTSYNALFLGNAERLLRVMQQSFPELGLTGKDCTETSWIKSVLYIAGYSSNTPAEILLQGRSTFKNYFKAKSDFVKEAIPETALEGLWKRLLEEDSPLMIWNPYGGMMARISGSQIPFPHRQGTKFKIQYLTLWQDEDNNASKHFDWIRRLYNYMAPYVSMFPRGAYVNYRDLDLGMNKNINTSFIEASLWGVRYFKDNFMRLVKVKSRVDPNNFFRHEQSIPPLPVQARY

>GbCKX55D

MSWNYYKNVLVLLEQVLIVPYGGATSIEGHTLSPNGGVCIDMTLMKRVKALHIWDMDVVVEPGIGWMELNEYLEPYGLFFPVDPGPGATIGGMCATRCSGSLAMM

>GbCKX56D

MLWNYYKNVLVLLEQVPIVPYGGATSIEGHILSPNGGVCIDMTLMKRVKALHIRDMDVVVEPGIGWMELNEYLEPYGLLFPLDPGPGATIGGMCATCCSGSLAMM

>GbCKX57D

MHFLRNLIILFLICIAIKINLCVPTIPSSLKTLPIDGHFDFKQVHHAAKDFGNRYSFLPSAVLHPKSASDIATTVKHIWEMGPGSHLTVAARGHGHSLQGQAQAHRGVVINMESLQGPKMKVHTGNFPYVDVSGSELWINILHETLKHGLAPKSWTDYLHLTVGGTLSNAGISGQAFRHGPQISNVRQLEVVTGKGEVVNCSEKQNSDLFYSVLGGLGQFGIITRARISLEPAPKMVKWIRVLYTDFATFAKDQEMLISGESTFDYIEGFVIINRTGLLNNWRSSFNSNDSAQASHFKSDGRTLFCLELAKYFNPEEMAIVNQEIMTSLSQLNHIPSTLFQSQVPYIEFLDRVHISEIKLRSKGLWEVPHPWLNLLIPRSNIHIFAQQVFGNILTNTSNGPILIYPVNKSKWDNRTSVVLPEEDVFYLVAFLSSAAPSSTGSDGLEHILSQNKRILELCEIDGLGVKQYLPHYSTNGEWRSHFGPQWEAFVHRKSTYDPLAILAPGQRIFQKAAPLSL

>GbCKX58D

MKFLQLSVLPFLIVTLSLNGATLAHSYGDFLHCLSLRISNSSTISKVIYTQNNPSYSSVLNASIHNARFSTPTTPKPYAIITPRKTSHVQSTIYCSKNHGFQLRIRSGGHDVEGVSYVAQVPFVILDLVNFRDVKVDTKNEVAWVQSGATTGELYYGIASKTQTLGFPAGICHTIGIGGHLSGGGFGILGRKYGLAADHIIDAKLVDANGRVLRRKSMGEDLFWAIRGGGGNTFGVVLAWKIKLVPVPPVVTVFTVNKNLEQNATKTFHRWQYIAHKLPRDLFTTVWVMKVNSSQVGRKTVQASFKGMFLGRIDVLIPLIQYAFPELGLARENCTEMSWVQSVLYFGALPIEPVEILLNRSALPRLSLKAKTDYIRQPMSETGIEGFMNMFLEEGTDFAITMIEAFGGKMDEIRENELPFPHRSGILFESVYIVQWTNEEDAGLCINWMRRLYSYMSSYASKSLRGAYYNYKDLDLGTNNINGYTSYEQASVWGLKYFRNNFKRLVRIKTMIDPMNFFSNEQSIPPLLSP

>GbCKX59D

MIAYLGRIVHDNDAESKLDDDVSSISKSLDLQGSIENGDVSSLASKDFGGLYSVKPLFLIKPSGAEDISRVVKLASRTSNLTVAARGNGHSINGQAMAEGGLVIDMCSTEKNHFEFLPINGSHYIDVSGGALWEDVLTRCVSRYGYAPRSWTDYLSLTVGGTLSNAGVSGQAFRYGPQTSNVTELEVVTGKGEITVCSETLNSELFFGVLGGLGQFGIITRARIKLQQAPDMVRWIRVVYSEFEEFTRDAEFLVTQKEGESFDYVEGFVFCNNDDPFNGWPSVPLDPGHEFNPTHISQTAGSVLYCLEVAFHYRNSDHPTVDTAVNGLLGRLRFVEGLKSQVDVSYTKFLLRVNRAEEQVKANGTWDGPHPWLNLFVSKSDVVNFDRTVFKTMLKDGVGGPMLIYPLLRSKWDDRTSVVLPEGEIFYIVALLRFVPNGPSVEKSVAQNREIVNWCIKVGLDFKLYLPHYQSKEDWERHFGNRWSRFVERKASFDLMAILAPGQNIFRRDPPNIIISREF

>GbCKX60D

MRGLPILVSIILFSISLVTSKSYPNSVVDNFLQCLPKHYRNPSKPIAKAIYTPTHPSFQSVYELHTYNLRVLTQANATHKPMAIVAALHESHVQAAVICAKESGLLVRIRSGGHDYEGLSFSSSVPFVIIDLSNLRSIKIDMKTETAWVQAGATTGELYYRIAEKSNVHAFPAGVCTTLGIGGHFTGGGYGNMLRKFGLSIDNVVDAQLINAKGRILNRKSMGEDVFWAIRGGGGTSFGIILSWKIKLVRVPPKVTVFQVAKTLEQGATVLVHKWLQVSHKLDKDIFIRIMPVTVAGTGNGNSTVRVSFIGHYLGRTNRLLPSVNASFPELGLQRKDCTEMSWIESTLYWAGFPNGTSTDVLLNRVPNKVFFKTKSDYLKNVMPKAGLETLWKVMMEIGNMWMQMSPYGGRMAEISESETAFAHRAGTLYLVQYTAHWSEGSSEATKKYVELMRKLYAEMAPYVSTKPREAFLNYRDLDIGSNNTDFEAAKVYGAKYFKGNFQRLAEPSNPVWGAIYRPSNASFPTILQAYAKNLRFSTPKPLAIVTTMQESHVQATVICAKSQGLQIRIGSGGHDYEGLSYVSEIPFVVLDMFNLRSKTVSYGFPAGAYPTLGVGEHFSGGGYGNLMRKYGLAVDNIFDARLVDVNGTILNRESMGEDLFWDIRGGGGGSFGVILSWKIKLGLQCTKNLKPGLNRYCLSMATSCPEVARRSIHKAQRLQRMSWMESTVFYPGFPRGTSIEVLLRRPQNGEDFYVVESLWRKDGWDLGI

>GbCKX61D

MATKLLLTFAICRLIVTVGLTLDPTELLLLGVDAQLSVDPTDVKAASLDFGLLTGAQPPLAVMHPASSQDVAQLVKAAYGSNFGFTVSARGHGHSINGQAQTANGVVVQMSGSIGGSGVASGRKPPYPRLWPQERFVDVWGGELWIDVLRSTLQHGLAPKSWTDYLYLSVGGTLSNAGISGQAFNHGPQISNVHELDVVTGKGELLTCSEEQNSEMFHAVLGGLGQFGIITRARISLEPAPQRVRWIRVLYSNFSTFTRDQEYLISLHEQPASQKFDYVEGFVIVDEGLINNWRSSFFSPHNPVKISSLEPNGGVLYCLEIAKNYHESTASTIDQEVESLLKKLNFIPASVFTTDMPYVDFLDRVHKAELKLRSKGLWEVPHPWLNLFVPKSKIADFDKGVFKGILGNKTSGPILIYPMNKNKWDHRSSAVTPDEDVFYLVALLRSALDNGEETHSLEYLTNQNRQILRFCDEAGITVKQYLPHYTTHQEWVDHFGNKWDRFYRLKMEFDPRHILASGQQIFTPTNMASWQ

>GbCKX62D

MATKLLLTFAICRLIVTVGLTLEPTELLRLGLTVDPFDVESASVDFGLMTKVEPLAVLRPSSAKDVAQLVKAAYESSHGFTVSARGHGHSINGQAQTGTGVVVQMSGVGSGGKPRVWGGDMYVDVWGGELWIDVLKSCLAYGLAPKSWTDYLYLSVGGTLSNAGISGQAFHHGPQISNVHELDVVTGKGELMTCSKEQNPELFHAVLGGLGQFGIITRARISLEPAPQRVRWIRVLYSNFSAFTKDQEYLISLHGNQKFDYVEGFVIVDEGLINNWRSSFFSPRNPVKISSLGSNNNGGVLYCFEITKNYHESTAETIDQEIEALLKKLNFIPTSVFTTDLPYVDFLDRVHKAELKLRSKGMWEVPHPWLNLFVPKSKIADFDRGVFKGILGNKTSGPILIYPMNKNKWDDKSSVVTPDEDVFYLVAFLRSALDNGEETQSLEYLTNQNRQILKFCDEDGMKVKQYLPHYKTQSEWKEHYGSKWDRFQRMKMKFDPRHILASGQNIFTPTFLSSSNMMVMDIPIPIKQKARESIDF

>GbCKX63D

MAVALPSFLTAIMIMSRLMAFIGITRNNDMSSKLQSLVIAPKLSYDPSAIESASQDFGHIEKAVPQAVLLPSSPWDIASLVNFSYSNSVPFIIAARGNSHSVNGQAMAKNGVVIDMTSMKNGNGTGIRIASDGSYADVGGQQFWIDVLNATLGLGLTPVSWTDYLYLTVGGTLSNAGISGQTFQYGPQISNVYEIDVITGTADFVTCSPNNNSDLFYAALGGLGQFGIITRARIPLEPAPKRVKWVRMLYTDFSDFTRDQELLISKNGRNDNKALNYLEGSLLLDQGSLDNWRSSFFQPQDQPKIISLITKFRIVYCLEIVKHYDGQTKTTVDKDLQQLLKGLSYLPGFMFEKDAKYEEFLNRVHSEELKLKAKGLWDVPHPWLNLFIPKSKISDFNDGVFKSIVLQRNITTGPVLVYPMNRKKWDDRMSAVIPDEEIFYTVGLLQSSGFDDWRTFEDQNKEILQFCEKAGIKVKQYLPHYTTKEGWVNHFGSKWSTFQKRKLQFDPKLLLSPGQRIFNNNE

>GbCKX64D

MIACLGRIVHDSDAESKLDDDVSTIFNSLNLQGSIENGDVSGIASKDFGGLYSVKPLYLIRPSGAEDISRVVKAAAGTPHLTVAARGNGHSINGQAMADGGYVIDMRSTGENHFNLLTVNGSPCIDVSGGALWEDVLRRCVSRFGLAPRSWTDYLSLTVGGTLSNAGVSGQAFRYGPQTSNVTELEVVTGKGDITVCSETQNPELFFGALGGLGQFGIITRARVKLQPAPDMVRWIRVVYAEFEEFTRDAEFLVSQKEDESFDYVEGFVFCNNDDPVNGWPSVQLNPDQEFNPAHLPQTAGPVLYCLELAVHYRNSDQPSTVDMAVSRLVGGLGFVDGLISQVDVSYMGFLLRVKRAEQDAKANGVWDNPHPWLNLFVSKSDIVDFDRTVFKKMVKNGIGGPMLIYPLLRSKWDSRTSVALPEGEIFYIVALLRFAPKGPSVEKKVAENREIVKWCIKEGLDFKLYLPHYRAKEDWKRHFGNQWTRLEKRKANFDPMAILAPGQRIFKRTNQ

>GbCKX65D

MAFSSTMILPLLLVLLSAFSATSKSVQENFMQCLDANSEHPIPISAFCSQTNSSFTSVLNSTAQNLRYLMPLVPKPEFIFIPVYESHAKSAVICAKRLAIHLRFRSGGHDYEGLSYASEIETPFILIDLIQLRSINVDIDDNSAWVQAGATVGEVYYRISEKSKTHGFPAGLCSSLGIGGHITGGAYGSMMRKYGLGADNVLDARIVDVNGEILDRAAMGEDLFWAIRGGGGASFGVILAWKIKLVAVPETVTVFTVPKTLEQGATKILYRWQQVADKLDDDLFVRVVIQVTKTSQKGKRTVTTAYNALYLGDAERLLQVMDQSFPELGLARKDCIETSWIKSVLYIAGFPSETPPEVLLEGKSLFKNYFKAKSDFVQQPILETALEKLWEMLLEEESPLMIWNPYGGMMANISDSAIPFPHRKGNLFKIQYVTSWYEGSKDATRKHMDWIKGLYDYMSAYVPTSPRGAYVNYRDLDLGMNHNNASYTEASVWGAMYFKGNFRRLVKIKSKVDPGNFFRHEQSIPVVLE

>GbCKX66D

MAISWPLVVSLLLSISSLATSASNSDSVHEAFVQCLLDNSHPSHPISEAIFTPQSPSYATVLQSYIRNLRFNETYTPKPFLILTALHQSHIQAAIICAKKGNIQMKIRSGGHDYDGLSYVATVPFFVLDMFNLRSIDIDMETETVWVQSGAILGELYYRISELSKTHGFPAGVCPTVGVGGHFTGGGYGNMMRKYGLTVDNIVDAYFIDVNGRIHDRKSMGEDLFWAIRGGGAASFGVVLAYKIKLVRVPEIITVFRVEKTLEDNATDIVDQWQHVASKLPKELFVRLVIDVVNSSTRTGGSTVRVSFISLFLGDSKTLVSIMNENLPLLGLSQSDCIETSWIRSVLFWTNITIDSPTDVLLNRTPSLSYLKRKSDYVKQPIPKTALEGIWEKMIELQPAQMIFNPYGGRMAEIASTATPFPHRAGNLWKIQYLANWNQGGAETAQRYIELTRKLHRYMTPFVSKNPREAFLNYRDIDLGVNHNDRGSYLEGRVYGIKYFKGNFNRLVHIKTKFDPTNFFRNEQSIPTLPY

>GbCKX67D

MNSSSSKLFILSTSFLLSISSITSNSVLDNFLQCLPIHSNTSNPITNSIYVPNNSSFQYLYELRANNLRTISSSTISKPLAILTARHASHVQAAVICAKIHSIQLRIRSGGHDYEGLSYVSDIPFVILDLFNLRSIKINITSETAWVQVGATTGELYYKIAEKSKVHGFPSGVCTTLGIGGHFTGGGYGNMIRKYGLSIDNVIDACLIDVNGTIHNRKSMGEDVFWAIRGGGGTSFGVILSWKIKLVHVPRKVTVFKVQRTLDQGATDIAYSWQHIAPKLPKYLFIRLQPEPITIGNGNKTIRVSFIGQFLGRSRKLMNLMNEEFPELRLQQNDCIEMSWVESTLFWAGFTNGTSIDVLLNRVVENKVFFKTKSDYYKNVIPKQGLVMLWEMLMDIGNIFVQLNPHGGRMDEISETETAVHQRGGYLFKVQYTVYWSESDGGIGAAKRYVEMSRRLYGAMAQYASSDPREAFLNYRDLDIGCNESNDTDFGVAEVYGTKYFNNNFMRLARVKAMVDPENFFKNEQSIPPLPSSH

>GbCKX68D

MPNPMRPYLILSVVFFFNLYHSMAVPDPTHQALLQCLTQSIPTDTASSIIVSKSNPSYTSVLRAYIRNARFNTSSTPKPLIIITPLDESHVSAAVICSQKLGFQLKIRSGGHDYEGLSYVFDNPFFVLDMFNLRSITVNMADETAWVGAGATLGELYYNIWKNSKVHGFPAGVCPTVGVGGHLSGAGYGTLIRKYGLSVDHVVDAKLVDVNGKILDRKTMGEDLFWAIRGGGAASFGVVLSYKIKLVPVPETVTVFRIERLLTENATNITFKWQTIAPTTDENLFMRMLLQPVTRNKKKTARISVIALYLGDSDSLVSLLQKDFPELSIGKSNCNETTWIDSVLWWANFNLGTPPTALLDRDLNDAGFLKRKSDYVQTPIPKSGLESLWQKMIELGKVGMVFNAYGGRMDQIKPDETPFPHRAGNLYKIQYSVNWDQPGSEADKNFTTQAKLLHDFMTPFVSKNPRSAYFNYRDIDVGSTKKWSYEEGKVYGESYFNGNYERLVDVKTAVDANNFFRNEQSIPPRSSSILHSSYGSATCIITAVNTCWYYIVFIFSLCSDLF

>GbCKX69D

MAVSFPIPSYFTAIFIISRLMSIIGISKHWNNKLLPPLDNITDKLSLDPSAIESASQDFGHIVKSIPKAVLQPSSIADIASLINFSYNSSIPFTIAAKGHGHSVRGQAMASDGVVVNMTSMKKHRNGTGIWVSNDGVYADVGGEQLWIDVLNATLKHGVAPVSWTDYLYLTVGGTLSNGGISGQSFRYGPQISNVYEMDVITGKAEIVTCSPNKNSELFYAALGGLGQFGIITRARIPLEPAPKRVKWIRMLYNDFTAFTRDQELLISINGRHDSHALDYLEGSLLMDHGSPDNWRSSFFPPKHHPKITSSITNHRIIYCLEVVKHYDDQTQNTVDKELEQLLKGLSYMPGFMFEKDVLYAEFLNRVLRGELKARSEGLWDVPHPWLNLFIPKSQIEGFNDGVFKGIVLERNITTGPVLVYPMNRKKWDDRMSAVIPDEEIFYTVGFLHSSGFDDWEAFDDQNKEILKFCEDAGIGVKQYLPHFTSKDEWVHHFGSKWETFQQRKFQFDPKMILSPGQRIFNNN

>GbCKX70D

MYYFRWLLGFLHLLVWGSIILVHAIPAPDPVQCNRTICTVSNSYGAWGDRKDCSVKSVVYPTTEEELRSAVAHANKNKLKVKVVSKFSHTIPKLACPSSLGHDSLFISTAKYDSGIEIDSVNLAVTADAGVALRDVIDKVEEAGLSLVAAPYWEGVSVAGMISTGAHGSSWWGKGGAVHDHVIGLSMIVPGNESEGYAKVKQIGAQDQLLNAAKVSLGILGVISKVKLSLERGFKRSITYNFTSDSSIENNFMEHGKKYEFGDITWYPSKHMAVYRYDSRVPMDTPGDGINDFLGFQSNEILISKSVRASEKLFESTKSVNGECTLADTTLWYKKQIGNGLKNNGQIFTGYPVVGRQGKMQTSGSCLYSPKTRIDASCAWDPRIKGLFFYESTAIFTATKFVDFIKDVKKLRDLKPENFCGIDHYNGFLIRYIKASKAYLGQSEDSIVVDFNYYRADEASTPRLNQDVMEEVEQMAFFKYGARPHWAKNRNLAFLKVQSKYLNFNMFIAVKKQLDPENMLSSEWSDEILFGKEGVRSDGCALEGLCICSEDRHCSPSKGYFCKPGLVYSEARVCRYSPSSSNL

>GbCKX71D

MVNLSLLLLHFLISSLSVSGSAATDQRNIMSCLNYYNISNYTISSNVHNHDYSILLNFSIQNLRFAEPTIPKPIAIILPENKEQLINTVVCCTKGPWEIRVRCGGHSYEGTSSVASDGAPFVIIDMMNLKSVSADLGNETAWVEGGATLGETYHAIAESSGIHGFSAGSCPTVGTGGHIGGGGFGFLSRKYGLAADNVIDALLLNAEGELLDRQAMGEDVFWAIRGGGGGIWGIVYAWKIKLLRVPKTVTSFIVSRPGTKAHVANLVNKWQHVAPNLEGDMYLSCAVGAGLPQAKSIGISATFNGFFLGRKREAVLILRRVFSELGVAEEDCKEMSWIESVLFFSGLGDGALVSDLKNRYLHDKHYFKAKSDYVRNPISLTGIRTAIDILEKQPRGYIIMDPYGGIMNNISNDSIAFPHRYGNLYTIQYLVEWHQEEKNRSNEYREWIRDFYDAMASHVSWGPRAAYVNYMDFDLGVMELINTSVLSEDTVEMARVWGEKYFLNNYDRLVKAKTLIDPNNVFKNQQGIPPSTTIGLEARTF

>GbCKX72D

MRIAYLDRTVHETDGEPKPNGGVSTLSKSIDLQGSVETGDKTTIASKDFGGLYSTKPLALIKPAGSDDVSRVIKAASRIPRMTVAARGNGHSINGQAMTNGGLVIDMRSTEENHFRLLNINGSFFIDVSGGALWENVLTRCVSRFGLAPRSWTDYLSLTVGGTLSNAGVSGQAFRYGPQISNVTELEIITGKGDITVCSETRNPELFFGSLGGLGQFGIITRAMVKLQPAPDMVRWIRVVYTEFDEFTRDAEFLVSRDDGESFDYVEGFVFCNNDDPVNGWPSVPLDPVHGFNQGIIPQTGASVLYCLEVAFLYQKGDHPSTVDKAVAGLLKPLRFVEGLKSEVDLSYVEFLLRVKRAEEQAKANGIWDAPHPWLNLFISKSDIVDFDQTVFKKMVKDGIGGPMLIYPLMRSKWDNRTSVALPDSEIFYLVALLRFVSRGPSVEESVAQNREIVEWCIKEGLDFKLYLPHYQSKEQWKRHFGNQWTRFVERKASFDPMAILAPGQNIFKRTHLS

>GbCKX73D

MGSPVCGFLKQNNIIFLRFFAILVLSCIPDGTNLCSNPSFDTPTIPPHSSSSSIPSSLKTLTLDGYFSFENLKHAAKDFGNICHYLPIAVLHPKSVSDISSTIKHILYMSSVTKLTVAARGRGHSLQGQAQAYQGVVINMESLDRPSMYIENGEVPYVDVSGSELWINILHETLKYGLSPKSWTDYLHLTVGGTLSNAGISGQAFRHGPQIDNVYQLEVVTGTGEVVTCSDKENADLFYGVLGGLGQFGIITRARISLGPAEKMVKWIRVLYSEFSTFSNDQEHLISSNNSFDYIEGFVMINRTGLLNNWRSSFNPKDPIQASQFSSDGKILYCLEMVKYFNPEKIDVLNQDIEKLLSELNYIPSTLFLSEVSYVEFLDRVHLSEIKLRSKGLWEVPHPWLNLLIPKSRILDFAEGVFGNIVKDNNNGPILIYPVNKAKWNNRTSMVTPEEDIIYLVAFLSSALPGTDGLEHIMTQNQHILDFCAKAQLGAKQYLPHYHTQDEWQAHFGTQWETFVQRKSAYDPLAILAPGQRIFQKAISIT

>GbCKX74D

MGFSFWLARLRSCSNKSAFSNAFRDSFYHYKSQLNTCQKNLPSTIAEKTNSHAFSWSSCLLPLAFAVSAGSLTFQSHNNHPSLCEPSNLDSRKVTIGGKASTEFVVKGTHKEVPQELIDELKAICQDNMTLDYDERFYHGKPQNSFHKAVNIPDVVVFPRSQEEVSQIVKSCNKHKVPIVPYGGATSIEGHTLSPNGGVCIDMTLMKRVKALHIRDMDVVVEPGIGWMELNEYLEPYGLFFPLDPGPGATIGGMCATRCSGSLAVRYGTMRDNVISLKVVLANGDIVKTASRARKSAAGYDLTRLMIGSEGTLGVVTEVTLRLQKIPEHSVVAMCNFPTIKDAADVAIDTMMSGIQVSRVELLDEVQVRAINIANGKNLPEVPTLMFEFIGTEAYSHEQTQIVQRIVSEHNGSDFVFAEDPEAKKELWKIRKEALWACFAMEPNFEAMISDVCVPLSNLAELISRSKQELDASSLVCTVIAHAGDGNFHTVILFDPNEEEHRREAERLNQFMVYTALSMEGTCTGEHGVGTGKMKYLEKELGIEALQTMKRIKTALDPNNIMNPGKLIPPHVCF

>GbCKX75D

MGFLHSSSLFPFLLVFIVSFSWPITSSADIYGDFLHCLSSSSISNLVYTQMNSSYSSILESTIHNSRFITPTSPKPWVIVTPLHVSHVQATIRCSKKHGLQLRTRSVGHDFEGVSYISESESPFVIIDLANLRSVQVDVENEVAWVRSGAIMGELYYEIAQKSGTLAFPGALCHGVAFGRYISGGGYRLLFRKYGLAADNVIDAEFIDANGRILKTENQWGKICFGIFVAAVVEALGLSFHGRLN

>GbCKX76D

MLNPKCFNFLTLFISLLSLPSPTIPQSSSLTNFLHCLHYGSDPIVSQSIYIASNPAFQTILQARIKNRRFLNPETLKPVAIVVPTRIDHVQGTVICAKDNGLQIRIRSGGHDYEGLSYRSNVTFIILDMSNFQSIDIDVKTETAWVQSGATLGELYYHIANKTNMHGFPSGICPTVGIGGHFSGGGYGNLMRKYGLSVDNILDIIAVDALGNVHDRASMGEDLFWAIRGGGAASFAVVVSYKIKLVRVPNKVTVFRKGFTLEQGATDLVHKWQQVAPNINEEFFIKVKLEPSFINGNQTVTATFIGFFLGRREKLLPIISKTFPELNLTQQDCHEMRWVETTLFWAGFPIGTPIETLLNRTIWTPLFFKNKSDYVKSVIPKESLNKIWKMTMAMMNRNDINKTRFDLECSPYGGKMNVIPESNTPFPHRKGNLFLIQYAFSWIDEGNNVSFNNIKKLRKLYDGMTPYVSKDPRECFLNYRDLDIGSNRSNETSFDDAKIYGRKYFKDNYTRLTKVKASVDPNNFFKYEQSIPPIK

>GbCKX77D

MKISSSIFPIISIFILLSISSVTPSDSDDSDDFDDFFQCLPKQSDSSIPITDAILTPNNSSFQYIYQLRANNLRTFLSATSRPVAIITARHPSHAQAAVICAKRHDFQLRIRSGGHDYEGLSYTSDVPFVILDMFNLNSIDIDMSTETAWVQAGATTGELYYRIAEKSNVHGFPSGVCTTLGIGGHFSGGGYGFLIRKYGLSIDNVIDAQLIDANGRILNRKSMGEDVFWAIRGGGTTSFGIILSWRIKLVRVPPRVTVFTVQRTLEQGATELAYRWQQVAPKLPKDLFIRLQPEPINNGGNNKTVRVSFIGHFLGQADGLLRLMNVSFPELGLTRNDCLQMSWVESTLYWAGFSNGSSIDVLLDRVAVNKVFAKEKSDYYKAVIPKQGLETLWQVLMDIENIFVQMNPYGGRMEEISDSETAFTHRAGNLFKVLYGIQWSESEGGVNATARYVELSRRLYNAMAPYASSNPREAFINYRDLDIGSNESDETDFEDAKEYGAKYFRNNFIRLADVKAKIDPKNFFKNEQSIPPLPSH

>GbCKX78D

MKISSSIFSLISIFILLLISSATSSDFDDFIRCLPRQSNSSLPITDAILTPNNSTFQSIYQLRANNLRTFLSATSRPVAIITALHPSHAQAAVICAKRHDFQLRIRSGGHDFEGLSYTSDVPFVILDMFNLNSIDIDMSTETTWVQAGATTGELYYRIAEKSNVHGFPSGVCTTLGIGGHFSGGGYGFLIRKYGISIDNVIDAQLIDANGRILNRGGTTSFGIILSWRIKLVRVPPRVTVFTVQRTLEQGATELAYRWQQVAPKLPKDLFIRLQPEPINNGGNNKTVRVSFIGHFLGQADGLLRLMNVSFPELGLT

>GbCKX79D

MKASSYTCFIVSIFVLFSISSAASYDPVDFDAFLQCLPQHSDHTVSIAGAILTPNNASFLSTYQLRANNLRILLSATSRPVAIITALHPSHAQAAVICAKRHGFQLRIRSGGHDYEGLSYISDVPFVILDMFNLKSIDIDMKTETAWVQAGATIGELYYSIAKKSKVHGFPSGVCTTVGIGGHFSGGGYGFLMRKYGLSIDNVIDAQLIDANGRILDRKSMGEDVFWAIRGGGTTSFGIILSWRIKLVRVPPRVTVFNVQRTLEQGATELAYRWQQVAPKLPQDLFIRLQPVPINNGGNNKTVRVSFIGHFLGQADGLLRLMNVRFPELGLTRNDCLEMSWVESALNWAGFPNGTSIDVLLNRVQVDRVFYKTKSDYYKAVIPKQGLETLWQVLMDIEDIFVQFNPYGGRMEEISESETARDLNCAFAHRGGNLFKALYRIQWSESEGGINATGRYVEMSRRLYNAMVPYASSNPREAFFNYRDLDVGSNESGETDFEVAKEYGAKYFRNNLMRLASVKAKIDPENFFKNEQSIPPLPTPPSH

>GbCKX80D

MTPPLSPPSLLPLLLVAFNICFSLAASNSVYESFVQCLKTRSNSSDNISDIVYSHSNATYETVLEQYIRNARFNTSSTPKPVIIITPLTESHVSAAVICSNNIGFQLRIRSGGHDFEGVSYVSDQPFFILDMFNLRSISINMADQSVWVQSGATLGELYYRIWEESKVYGFPAGVCPTVGVGGHISGAGYGNMVRKYGLSVDYVVDAKIVDVNGNILDRKAMGEDLFWAIRGGGGASFGVILAFNIKLVDVPETVTVFKLERTLEQNATDVVYKWQSVAPTTDDNLFMRMLVQPVTLNKQKTIKISIMALYLGDVNSVVPLLAEDFPELGLVTEDCFEMSWIESALWWASFGKGTSPTVLLDRESYHVKFMKRKSDYVKTPISKDGLQWLWKKMIELEEPGLVFNPYGGKMNEIKETETPFPHRAGNLFKIQYSINWKDMGIEADKRSRSLVNRLHGYMTSFVSKNPRSAYLNYRDLDIGITKNWSYQEGKVYGESYFNGNFERLVDVKTVVDPHNFFRNEQSIPPRTIKAWNEKNEGSIPPSTSKAWNKSKPYVMIILFMAIGHII

>GbCKX81D

MEISKPFVVFFSLVFFNLSFSWAAPDPTYQSLLQCLSELIPSPNVSAVIVSNNNPSFASILESRIHNARFNRTSTLKPTIIITPSDESHVSAAVICSQKVGFQLKIRSGGHDYEALSYTSDKPFFLLDMYNLRDVSVDIPDESAWVQTGATLGELYYHIWEKSNVHGFPAGVCPTVGVGGHIGGAGLLNFKNTE

>GbCKX82D

MAISLLFSLLFLNISISSAASNPTYQSLLQCLSQSINPSQNVSTILFSNTNPSYASVLQAYIRNARFNTSSTPKPVIIITPLEESHVSAAVICSQKVGFQLKIRSGGHDYEGLSYVSDKPFFVLDMFNLRSISIDMTDESAWVETGATLGELYYNIWEKSNVHGFPAGLCPTVGVGGHLSGAGYGTLMRKYGLSSDYIVDAKIVNVDGKILDRKAMGEDLFWAIRGGGAASFGVVLAYKVKLVRVPETVTVFRLERLLADNATDIALKWQSIAPTTDENLFTRMLLQPVTRNRQRTMRVTVNGLYLGNADGVVALLSKDFPELGLKNENCTEMRWIDSVLWWANFDAGTPPTALLDRNVNDADFLKRKSDYVQTPISKNGLESLWQKMVELGNVGLACNAYGGRMDEIDDKETPFPHRKGNLYKIQYSVNWNEPGNETEMNRTSQAKALHEFMTQFVSKNPRRAYLNYRDIDIGVAENWSYEEGKVYGESYFAGNYERLVDVKTAVDPNNFFRNEQSIPPRTK

>GbCKX83D

MAFPSISSLFSLLLVLHLSSSTTASTCHAKSFKLNPIQEKFIQCFKANSEIPIPASTEFFTPNNASFSTVLQSTAQNLRYLEPSVPKPEFIIMPLNESHVQAAVICSKELGIHMRVRSGGHDYEGMSYVSAIESPFILVDLSKLRSVKVDIEDNSAWIEAGATIGEVYYRIYEKSKIHGFPAGLCTSLGVGGHITGGAYGSMMRKYGLGVDNVIDARIVDVNGRVLDRAAMGEDLFWAIRGGAGGSFGIILQWKIQLVPVPSTVTVFTITKSLQQNSTKIFHRWIEVADNLDDDLFIRVIIQTAMINGEKTVTTSYNSLFLGEADRLVEIMQQSFPELGLTRKDCIETSWIKSVLYIAGYPSNTPPDVLLQGKSTFKNYFKAKSDFVKSNIPETALEGLWKRFMEEDIPLMIWNPYGGMMARISESETPFPHRKGNKIMIQYVSAWQDGDKNESKHIDWIRRLYNYMAPYVSMFPRTAYVNYRDLDLGTNKNASTSFIEASGWGVKYFKDNFNKLVKVKTKVDPENFFRHEQSIPPLPVEARF

>GbCKX84D

MEKWRATTNLRRSLKSILNRQLSSVSEFRYLNEKRSCQSSFNLIRDCKSLGQVNAIQHRCFSSASTLVQRNPSFSTLNSDDISYFKGLLGEKSVIQDEDRLETVNTDWMHKYKGSSKLLLLPRSTEEVAQILRYCNSRCLAVVPQGGNTGLVGGSVPVFDEVIVNVSSMNNIISFDKVSGILVCEAGCILENLISFLDNQGFIMPLDLGAKGSCQIGGNVSTNAGGLRLVRYGSLHGNVLGLEAVLANGDVLDMLGTLRKDNTGYDLKHLFIGSEGSLGIVTKVSILTPPKLSSVNIAFLACNDYSSCQKLLMEAKRKLGEILSAFEFLDTEAMNLVLHQLDGVRNPLPASMHNFYILIETTGSDESYNREKLEAFLLSSMEGGLISDGVLAQDINQASSFWRIREGVPEALMKAGAVYKYDLSLPVEKMYDLVDDMRIRLGDLATVVGYGHLGDGNLHLNVSAPEYDDKILEQIEPYVYEWTSKHRGSISAEHGLGLMKANKIYYSKSTETVQTMASIKKLLDPNGILNPYKVLPHSLNS

>GbCKX85D

MQKHKMKSNLPIPLLFLSLLFSFSWAALGLTHQQQHHSFLQCLNRHFGNANSISTVIYTQTNSSFSSVLEFSLRNARFSTPNTLKPLVIVTPSHVSHIQATINCSRTHGLQIRIRSGGHDYEGLSYVSQVPFVIIDLINLRSIDVDAENKTAWIQAGATIGELYYRIAEKSSTLAFPAGVCPTVGVGGHFSGGGYGMLMRKYGLAADQIIDAQLVDVNGKLLDKNSMGEDLFWAIRGGGAASFGVVVAWKVKLVPVPSTLTVFTVNRTLEENGTMLVHKWQSIAPKIHEDLYIRLFLRAVNSSQQEGKRTIQASFVSLYLGRADELIDLMQESFPELGLVKEDCIEMSWIQSIMYFPSDIPEDAPLEILLNRTGSAGIFKGKSDYVTQTIPETALEGLWQRFYEDETESLEILFSPYGGNMDDIPETETPYSHRAGNLFNIHYVVGWSEEDASESQRYINFMRRLYRYMEPYVSKSPRRAYMNYRDLDLGTNNIGPYTSYKQASKWGLPYFDKNFNRLIHVKTLVDPTNFFRYQQSIPSLSRG

>GbCKX86D

MGKLKAVVVVTMISTVLLSILWRATLHLEDNESFVRCLLDHSHPSHPISSAIYTPKSSSFSSVLESYIRNLRFNESSTPKPFLILTALHESHIQAAVTCGKSHGVQLKIRSGGHDYEGLSYVSTLPFFLLDMFNLRSIDVDIETETAWVQTGATLGEVFYRIAEKSKTHGFPAGVCPTVGVGGHISGAGYGNMMRKYGVSADNVLDALIIDANGRLLDRQSMGEDLFWAIRGGGGASFAVVLAYKIKLVRVPETVTVFQVDRTLEEDATDIVDQWQHVAYNLPQELFIRLMLDVVVKRSGEKTLRASFVSLFLGDSESLLSIMKERFPKLGLSKSDCIETSWVKSVLFWSNIPLETDIQVLLDRTPQTLDYLKRKSDYVREPIPKAGLESLWKKMMELEKPRMYFNPYGGKMAEIAAEEIPFPHRAGNLWKIQYLANWNEAGIEAANRYIDLTRRLHEFMTPFVSKNPRQAFLNYRDADLGSSSHGKASYSEARLNGMKWFMGNFDRLVQIKTEVDPTNFFSYEQSIPLLPHQVHLDDDM

>GbCKX87D

MSFFFPEMKASQYFSMSLFLLILLISCPWLISANPHLNNFLGCLDSFYSNHISQVIYTQNNASYSSVLNATIQNLRFSTPTTPKPLVIVTPLQTSHIQATIRCSRTNGLNLRIRSGGHDFEGLSYVSQVPFVVLDLTNFRSVKIDVKNKVAWVQSGAILGEFYSEIAKRSRTLAFPAGICHTVGVGGYLSGGGYGLLLRKYGLAVDNVIDAVFIDVNGRILKRKSMGEDLFWAIRGGGGGSFGVVLSWKVKLVSVPSTVTVFTIRKTLEENATNLVHQWQSVGHKLPGDIFSAVTMRKVNRNGKTTILVAFSSFFLGETNALITLMKAGFPELGLKKEHCTEMSWIESILYFGQIQNKSIDVLLDRSYKSPLNAPWFKTKLDYVKNPIPKAGFEKIWSKLYEEDAETAAMAFIAYGGKMAEIPESATPFPHRDGNLYHIAYTVGWDGEENTKSQRYMNWIRKFYSFMTPFVSKSPRGAYVNYRDDDIGTNNKKGETSYAKASVWGRKYFKNNFDKLIYIKTKVDPHNFFKHEQSIPVSV

>GbCKX88D

MLVSFPGQNNMLVLRSFMILFFSCITIKINLCFPSIPSSLKTLSIDGHFDFEQVEHAAKDFGNRYSYLPLAVLYPKSVSDVATTVNHVWQIGGGSELKVAARGHGHSLQGQAQAHRGVVINMESLQGLKMQVHTGNFPYVDVSGGELWINILRQSLKHGLAPKSWTDYLHLTVGGTLSNAGISGQAFRHGPQISNVYQLEVVTGKGEVVNCSEKQNSDLFYSVLGGLGQFGIITRARISLEPAPEMVKWIRMVYTDFATFTRDQEKLISGQSTFDYVEGFVIINRTGLLNNWRSSFNPQDPLQASQFKSDGRTLFCLELAKYFNHEDMVLVNQEIKTSLSQLNHIPSTLFISEVPYMEFLDRVHISEIKLRSRGLWEVPHPWLNLFIPKSKIHSFAQEVFGNILTDTSSGPILIYPVNKSKWDNRTSVVIPEEDVFYLVAFLSSAVPSSTGNDGLEQILIRNKRILDYCEIAGLGVKQYLPHFSTQGEWKSHFGPHWEAFIRRKSTYDPSAILAPGQRIFQKPIAYS

>GbCKX89D

MFRSLSLKRSFKTHLKTLIHCPNHPQPPPPPSPVLFSSIRALSTASPPPSSSSDSELRKYLGYTALLAFCGVATYYSFPFSENAKHKKAQLFRYAPLPEDLHTVSNWSGTHEVQTRHFHQPENLKQLEELVKESNEKRVKLRPVGSGLSPNGIGLARGGMVNLALMDKVLEVDKEKKRVRVQAGIRVQQLVDEIKDYGLTLQNFASIREQQIGGILQVGAHGTGAKLPPIDEQIISMKLVTPAKGTIELSKEKDPELFYLARCGLGGLGVVAEVTIQCVERQELVEHTTVSNLKDLKKNHKKMLSENKHVKYLYIPYTDTVVVVTCNPVSKWRGPPKFKPKHTTDEAMQDIRELYKESLKKYRARDITTKSSDSNEPNINDFSFTELRDKLHSLDPLNKDHVMKVNHAEAEFWRKSEGYRVGWSDDILGFDCGGQQWVSETCFPAGTLSKPSMKDLEYIEELKKLIETNELPAPAPIEQRWTARSQSPMSPASSSAEDDIFSWVGIIMYLPTMDARQRKEITEEFFHYRHLTQSQLWDKYSAYEHWAKIEVPKDKEELEALQARLKTRFPVDAYNKARRELDPNRILSNNILEKLFPLSDNV

>GbCKX90D

MPIIPVLYSNKLNALVLLKCLFLLVVLVGCSPPGEPVKCSTKDSNCTVTNSYGAFPDRTVCRAGNVVYPTSEQELVFIVSAATEAQRKMKVVTHFSHSIPKLVCPDGQDGLLISTKNLNRVVKINLAAMTMTVESGVTLRQLINEVAKAGLALCTVLVGTDHWRAFRYRSSWELVVGERELRS

>GbCKX91D

MPAIPMLFSNKFNVLLLWKCLCLLVVLVGCNPPGEPVKCSTKDSNCTVTNSYGMFPDRAICRAGNVAYPTSEQELVSIVSAATKSKRKMKVVTHFSHSIPKLVCPDGQDGLLVSTKNLNRVLKTDTDAMTMTVESGVTLRQLINEAAKAGLALPYAPYWWGLTIGGLLGTGAHGSSLWGKGSSVHDYVVEMRIVSPAKAEDGYAKVWELNERDKDLDAAKVSLGVLGVISQVTFKLQPLFKRSITYVRKDDTDLGDEAVTFGKLHEFADIFWYPSQRKAIYRIDDRVPINVSGNGVYNFTPFRSTLSLVLALVRSSEETQESSGDAEGKCLNAKLVTSTLQSSAYGLTNNGAIFTGYPVIGFHNRLQSSGTCLDSLEDSLITACPWDPRIKGEFFHQTTFSISLSVVKSFIQDVQKLVSMDPKSLCGLELYNGILMRYVKASTAYLGKQEDAVDFDITYYRSKDPMAPRLYQDVLEEIEQMALLKYNALPHWGKNRNLVFDGEMKRYKNGGEFLKVKNKYDPWGLFSSEWTDQVLGLRNGVIVLKEGCALEGLCICSQDVHCAPSKGYLCKPGKTFPDARVCARVNTKT

>GbCKX92D

MKRNMLFLRSFMILVLSCIAIKINLCFPNILSSLKTLPIDGHFNFEQLHHAAKDFGNRYSFLPLAVLHPNSVSDIATTVKHIWQMGPGSDLTVAARGHGHSLQGQAQAHGGIVINMKSLQGLKMQFHIGNLPYVDVSGGELWINILREGLKHGLAPKSWTDYLHLTVGGTLSNAGISGQAFRHGPQISNVHQLEVVTGKGEVVTCSGKQNSDLFHGVLGGLGQFGIITRARISLEPAPEMVKWIRVLYTDFATFIRDQEELISGESTFDYVEGFVIINRTGLLNNWRSSFNPQDPVQASKFKSDGRTLFCLELAKYFNRDETAVVNREIHSSLSQLNHIPSTLFVSEVPYIEFLDRVHISEIKLRSKGLWEVPHPWLNLLVPRSKIQTFAQQVFGNILTDTSNGPILIYPVNKSKWDNRTSVVTPDEDVFYLVAFLSSAVPSSTGTDGLDHILIQNKRILEFCEIARLGVKQYLPHYSTQGEWKAHFGSRWEVFVRRKSSYDPLAILAPGQRIFQKAVPYSQ

>GbCKX93D

MVGKITRAYEREVKEGDVKMASPKFGMSILFFFLCISFCSSTDQSFQQCFSSHLPPSNITYDVIFTQNSSQYSSILQSSIRNLRFSNASKPRYLVTPYNEDHIQATIICSKEHHMHVRVRSGGHDYEGLSYISDVPFIVIDLFHIRSVMVDIKNEYAWVGAGATLGELYYSISAKSNVHGFPAGSCPTVGVGGHISGGGFGTIFRKYGLAADNVIDAKMIDVNGNVLDRKSMGEDLFWAIRGGGGASFGVIFSWKLKLVRVPLTVTVFKTVKSLEQGATKLVQKWQNIAYKFHHDLFVHAVIQVTNPNSNQNPTVQVSFDCLFLGTTERLLSSIQRSFPELGVTQENCTEMSWIQSVLYFAGYSIAESADVLLNRTTQSTQSFKGKSDYVKEAIPKTGLEGLFKMVVEEETSVLILTPYGGRMKQIKSSATPFPYRSEYLYGIQYMISWDVAEETGKRIGWMRRLYKYMEPYVSTAPRAAYFNYRDLDLGRNSYPNTSYVESSEWGLKYFNHNFNRLVRVKTLADPHNFFWNEQSIPVLRLE

>GbCKX94D

MSDLQAPLRPKRKKGLVDFLVHFRWIFVIFFVLPFSTLYYFLIYLGDVRSEMKSYKQRQKEHDENVLKVVKRLKQRNPKKDGLVCTARKPWIAVGMRNVDYKRARHYEVDLSAFRNILEIDKQRMIARVEPLVNMGQITRVTVPMNLSLAVVAELDDLTVGGLINGYGIEGSSHIYGLFSDTVVAYEIVLADGRVVRATKDNEYSDLFYAIPWSQGTLGFLVAAEIKLIPVKEYMRLTYTPVVGNLQDLAQGYMDSFAPRDGDQDNPEKVPDFVEGMVYSPTEGVFMTGRYASKEEAKKKGNKINNVGWWFKPWFYQHAQTALKKGEFVEYIPTREYYHRHTRCLYWEGKLILPFGDQWWFRFLLGWLMPPKVSLLKATQGESIRNYYHEMHVIQDMLVPLYKVGDALEWVHHEMEIYPIWLCPHRLFKLPVKTMVYPEPGFEQHRRQGDTPYAQMFTDVGVYYAPGPVLRGEVFDGAEAVRKLEQWLIKNHSFQPQYAVSELNEKDFWRMFDADLYEHVRRKYGAVGTFMSVYYKSKKGRKTEKEVQEAEQAHLETAYAEAD

>GbCKX95D

MIACLGRIVQDTDADSIPDDDVSTLSESLDLQGTIESGGITGVAGKDFGGLYSVKPLALIKPSGTEDIARVVNAASRTSHLTVAARGNGHSINGQAMADGGFVIDMRSTEENHFKPLTIDGSHYIDVSGGALWEDVLKRCVSMFRLAPRSWTDYLSLTVGGTLSNAGVSGQAFRFGPQTSNVTELEVVTGKGEITVCSETQNSELFFGALGGLGQLGIITRARVKLQRAPDMVRWIRVVYTEFEEFTRDAEFLVTQEEGESFDYVEGFVFSNSDDPINGWPSVPLDPDHEFNPAYIPQTAGSVLYCLEVALHYRNSDRPSTVDTAVSRLLERLGSIQRLKFQLNVSYVEFLLRVKQGEEHAKANGNWDSPHPWLNIFISKSSIVDFDRTVFRKMLKDGIGGPMLIYPLLRSKWDSRTSVVLPEGEIFYIVALLRFVPKGPTVEKLVAQNHEIIQWCNKEGLDFKLYLPHYQSKEDWKRHFGNQWTRFVERKTSFDPMAILAPGQKIFKRTHIIKP

>GbCKX96D

MVMSFQFPAYFTAIFIITRVMSIMKISKPLDVHHKDIRAVDLATKLSVDPSAIESASRDFGGIVKAEPEAVLHPSAPQDIAALIKFSYSNSVPFGIAAKGHGHSVRGQAMAENGVVVDMRSMANNRRNGTGIRISIDRLYADVGGEQLWIDVLNATLEYGLAPVSWTDYLYLTVGGTLSNAGISGQTFRYGPQISNVLEMDVITGKADFLTCSPRMNSELFYAVLGGLGQFGIITRARIPLQPAPKGVKWVRLLYDDFSSFTKDQELLISKNGRKDKSALDYLEGSLLMDQGSPDNWRSSFFPHKDHPRIISLITKHGIIYCLEIVKHYDDRTKHTVDKEMKQVLQGLNYMPGFMFGKDVGYEEFLNRVRSGELKLKSQGLWDVPHPWLNLFIPKSQISDFNNGVFRGIVLERNITTGPVLVYPMNRQKWDDRMSAVIPDEEIFYTVGFLHSSGFDTWEAFEDQNKDIMRFCNKTGILVKQYLPHYSTKEEWVHHFGSKWKVFQHRKYQFDPRMLLSPGQRIFNNN

>GrCKX01

MAVSFPIPSYFTAIFIISRLMSIIGISKHWNNKLLPPLDNITDKLSLDPSAIESASQDFGHIVKSIPKAVLQPSSIVDIASLINFSYNSSIPFTIAAKGHGHSVRGQAMASDGVVVNMTSMKKYRNGTGIWVSNDGVYADVGGEQLWIDVLNATLKHGVAPVSWTDYLYLTVGGTLSNGGISGQSFRYGPQISNVYEMDVITGKAEIVTCSPNKNSELFYAALGGLGQFGIITRARIPLEPAPKRVKWIRMLYNDFTAFTRDQELLISINGRHDSHALDYLEGSLLMDHGSPDNWRSSFFPPKHHPKITSSITNHRIIYCLEVVKHYDDQTQNTVDKELEQLLKGLSYMPGFMFEKDVLYAEFLNRVLRGELKARSEGLWDVPHPWLNLFIPKSQIEGFNDGVFKGIVLERNITTGPVLVYPMNRKKWDDRMSAVIPDEEIFYTVGFLHSSGFDDWEAFDDQNKEILKFCEDAGIGVKQYLPHFTSKDEWVHHFGSKWETFQQRKFQFDPKMILSPGQRIFNNN

>GrCKX02

MYYFRWLLGFLHLLIWGSIILVHAIPAPDPVQCNRTICTLSSSYGAWGDRKDCSVKSVVYPTTEEELRSAVAHANKNKLKVKVVSKFSHTIPKLACPSSLGHDSLLISTAKYDSGIEIDSVNLAVTADAGVALRDVIDKVEEAGLSLVAAPYWEGVSVAGMISTGAHGSSWWGKGGAVHDHVIGLSMIVPGNESEGYAKVKQIGAQDQLLNAAKVSLGILGVISKVKLSLERGFKRSITYNFTSDSSIENNYMEHGKKYEFGDITWYPSKHTAVYRYDSRVPMDTPGDGINDFLGFQSNEILISKSVRASEKLFESTKSVNGECTLADTTLWYKKQIGNGLKNNGQIFTGYPVVGRQGKMQTSGSCLYSPKTRIDASCAWDPRIKGLFFYESTAMFTATKFGDFIKDVKKLRDLKPENFCGIDHYNGFLIRYIKASKAYLGQSEDSIVVDFNYYRADEASTPRLNQDVMEEVEQMAFFKYGARPHWAKNRNLAFLKVQSKYLNFNMFIAVKKQLDPENMLSSEWSDEILFGKEGVGSDGCALEGLCICSEDRHCSPSKGYFCKPGLVYSEARVCRYSPSSSNL

>GrCKX03

MVNLSLLLLHFLISSLSVSGSAATDQRNIMSCLNYYNISNYTISSNVHNHDYSILLNFSIQNLRFAEPTIPKPIAIILPENKEQLINTVVCCTKGPWEIRVRCGGHSYEGTSSVASDGAPFVIIDMMNLNRVSVDLGNETAWVEGGATLGETYHAIAESSFIHGFAAGSCPTVGTGGHIGGGGFGFLSRKYGLAADNVIDALLLNAEGELLDRQAMGEDVFWAIRGGGGGIWGIVYAWKIKLLRVPKTVTSFIVSRPGTKAHVANLVNKWQHVAPNLEGDMYLSCAVGAGLPQAKSIGISATFNGFFLGRKREAVLILRRVFAELGVAEEDCKEMSWIESVLFFSGLGDGALVSDLKNRYLHDKHYFKAKSDYVRNPISLTGIRTAIDILEKQPRGYIIMDPYGGIMNNISNDSIAFPHRYGNLYTIQYLVEWHQEEKNRSNEYREWIRDFYDAMASHVSWGPRAAYVNYMDFDLGVMELINTSVLSEDTVEMARVWGEKYFLNNYDRLVKAKTLIDPNNVFKNQQGIPPSTTIGLKARTF

>GrCKX04

MEILRLGIDGQLSVDPSDVETASLDFGLLTRGQPLAVLHPVSAQDISQVVKAVYGSNQGMTVSARGHGHSINGQAQTTNGVVIQMSGRKGGNKPPRPRVWSEERYVDVWGGELWIDVLKSTLEYGLAPKSWTDCLYLSVGGTLSNAGISGQAFNHGPQISNVYELDVVTGKGEVLTCSEDENTELFHAVLGGLGQFGIITRARISLEPAPKMVRWIRVLYSNFSAFTSDQEHLISLHAEPSNQKFDYVEGFVIVDEGLINNWRSSLLSPQNPVKISTLFPSGGVLYCLEIAKNYHESTAQTIDQEVEFLLKKLNFISTSGFTTDLLYVDFLDRVHKAELKLRSKGLWEVPHPWLNLFIPSSKIAEFDKGLFKGILGNKTSGPILIYPMNKNKWDHRSSVVTPDEGVFYLVALLRSALDNEEETQSLEYLNNQNRQILRYCDEAGIKVKRYLPHYTTQQEWMDHFGNKWDRFYEMKMEFDPRHILASGQRIFTPTFPSSSNMPS

>GrCKX05

MKFLQFSVLPFLIVILSLNGATLAHPYGDFLHCLSLRISNSSAISKVFYTQNNPSYSSVLNASIQNTRFSTPTTPKPYAIITPRKTSHVQSTIYCSKNHGFQLRIRSGGHDVEGVSYVSQVPFVILDLVNFRAVKVDTKNEVVWVQSGATTGELYYGIASKTQTLGFPAGICHTIGIGGHLSGGGFGILGRKYGLAADHIIDAKLIDANGRILNRKSMGEDLFWAIRGGGGNTFGVVLAWKIKLVPVPPVVTVFTVNKNLEQNATKIFHRWQYIAHKLPNDLFTAVWIMKVNSSQVGKKTVQAGFRGMFLGGVDELIPLIQHEFPELGLAKENCTQMSWVQSILYFGGLPIQPVEILLNRNALPRSSLKAKTDFVKEPMPETGIEGFMNMFLEEEADFAITMIEAFGGKMDEIQENELPTHIEQASYAGTYINWIRRLYSYMASYVSKSPREAYYNYKDLDLGTNNVNGYTSYEQASVWGLKYFKNNFKRLVQIKTMIDPMNFFRNEQSVPHLWSP

>GrCKX06

MRIAYLDRTVHETDGEPKPNGGVSTLSKSIDLQGSVETGDKTTIASKDFGGLYSTKPLALIKPAGSDDVSRVIKAASRIPRMTVAARGNGHSINGQAMTNGGLVIDMRSTEENHFRLLNINGSFFIDVSGGALWENVLTRCVSRFGLAPRSWTDYLSLTVGGTLSNAGVSGQAFRYGPQISNVAELEIITGKGDIMVCSETRNPELFFGSLGGLGQFGIITRAMVKLQPAPDMVRWIRVVYTEFDEFTRDAEFLVSRDDGESFDYVEGFVFCNNDDPVNGWPSVPLDPVHGFNQGIIPQTGASVLYCLEVAFHYQKGDHPSTVDKAVAGLLKPLRFVEGLKSQVDLSYVEFLLRVKRAEEQAKANGIWDAPHPWLNLFISKSDIVDFDQTVFKKMVKDGIGGPMLIYPLMRSKWDNRTSVALPDSEIFYLVALLRFVSRGPSVEESVAQNREIVEWCIREGLDFKLYLPHYQSKEQWKRHFGNQWTRFVERKASFDPMAILAPGQNIFKRTHLS

>GrCKX07

MKSLYCSLPFLFVVVCSLSWVNASANSHENFLDCLSSYHPQESSSISKVVYTETNSSYSEILESSIQNYRSFTTNTPKPLVIVTPLNISHVQATIHCSKKHGLQIRIRSGGHDFEGLSYVSQVPFIVIDLVHFRSIDVDVENEEAWIQSGAITGEVYYRINERSTNLTFPGAVGHTVGIGGFISGGGYGLLFRKYGLAADNVIDAMFVDANGRVLDRKLMGEDLFWAIRGGGGGSFGIVLSWKVKLVHVPSTVTVAAVRRTLEQNATQLLHRWQYVAPNLPNDVYSVVSISTTNSTENGERTVVATFVSVFQGGANELIPLMQERFPELGLVREDYIEMTWIESILLLTGLTNQTKEVLLDRSYKNFFLSPSFKGKSDYMRKPMPEIVIQGLLSQLLEDEARISTLNIIAYGGKMDEIPETETPFPHRKGTLYKISYYVGWQEEDNSNPQRYISWIRKVYKYMGPFVSKYPREAYLNYRDLDIGRNNNEGKASYKQASVWGRKYFKENFDRLTYVKAKIDPENFFRHEQSIPPRFH

>GrCKX08

MKSLHDCLPLLFVVVFSLSWVNASANSNDYFLDCLSSYHPDEFTSFSKVIYTETNSSYSAVLESSIRNPRFSTPNIPKPLVIITPSNISHVQATIHCSKKHGLQIRTRSGGHDFEGLSYVSQVPFVVIDLVHFRSIDVDVKKEEAWIQSGAITGEVYYRINEKTTNLTFPGALCHTVGIGGFISGGGYGFLFRKYGLAADNVIDAQFVGANGRVLDRRLMGEDLFWAIRGGGGGSFGIVLSWKVKLVHVPSTVTVFSVGRTLEQNATQLLHRWQYVAPNLPNDVYSVVSISSMNSTENGERTVLATFTSVFQGVADELIPLMQERFPELGLLREDYIEMTWIESILFWNQLSNETSEILLDRSNRNSLVPLSYKSKSDYVRKPMPEIALQGLWSRLLKVNETSTAIVNIISYGGKMDEIPETETPFPHRKGTLYKINYNIVWQEEENSNPQRYISWMRTLYSYMGPFVSKSPRAAYVNYRDLDIGRNNDDGKTSYKQASVWGRKYFKNNFDRLVYVKTKIDPENFFKHEQSIPPRFH

>GrCKX09

MKSLHRSLPLLFVVVCSLSWVNVSANSHDDFLDCLSSYHPDESSSISKVTYTETNSSYSAVLESSIRNHRFSTPNTPKPLVIVTPLNISHVQATIRCSKKHGLQIRTRSGGHDYEGLSYVSHVPFVVIDLVHFKSVEVDVENEEVWVQSGAIVGEVYYRINERSTNLTFPGAHGYTVGIGGFISGGGDGLLSRKYGLAADNVIDAQFVDASGRVLDRRSMGEDLFWAIRGGGGGSFGIVLSWKIKLVHVPSIVTVFSVGRTLEQNATQLLHRWQYVAPNLPNDVYSLVAISAWKASENETRTVLVTFTSVFQGGANELIPLMQERFPELGLVKEDFIEMTWIESVLFINGLSNETSEALLERNRSLLPPSFKSKSDYVNEPVPEIALQGLWPQLLEVDEAISAVQTFIAYGGMMDEISETETPFPHRKGTLYKIHYNIGWQEEENIRSQRYISWMRKLYSYMGPFVSKSPRAAYVNYRDLDIGRNNDDGKASYKQASVWGRKYFKNNFDRLVSVKTKVDPENFFKHEQSIPPHFH

>GrCKX10

MKSLHHSLSLLFIVVCSLSWVSASANSHDDFLECLYSYHPKESSSITQVIYTETNSSYSAVLDSSIRNHRFSTPNTPKPLVIVTPLNVSHVQATIHCSKNHGLQIRTRSGGHDFEGLSYVSHVPFVVIDLVNLRSVDVDVENEEAWVQTGATVGEVYYRINERSTNLTFPAAVVRTVGIGGSISGGGDGLLFRKYGLSADNVIDAQLVDANGRVLDRRSMGEDLFWAIRGGGGGSFGIVISWKIKLVHVPSTVTVFSVGRTLEQNATQLLHRWQYVAPNLPNDVYSLVAISTTNASENRTKTVLATFISLFQGGANEFIPLMQERFPELGLVKEDFIEMTWIESLLLMNGVSNETSEILLDRSNRYSLLPPSFKSKSDYVREPMPEIALQGLWPQLLEVDEGGIAVQNIIAYGGIMEEISETETPFPHRKGTLYKINYNIGWLEEENNNSQRYISWMRKLYSYMGPFVSKSPREAYVNYRDLDIGRNNDDGKASYKQASIWGRKYFKNNFDRLVYVKTKTDPKNFFKHEQSIPPRFH

>GrCKX11

MKSLHHSLSLLFIVVCSLSWVSASANSHDDFLECLYSYHPKESSSITQVIFTESNSSYSAVLDSSIRNHRFSTPNTPKPLVIVTPLNISHVQATIHCSKKQGLQIRTRSGGHDFEGLSYVSHVPFVVIDLVNLRSVDVDVENEEAWVQSGATVGEVYYRINERSTNLTFPAAVARTVGIGGSISGGGDGILFRKYGLSADNVIDAQLVDANGRILDRRSMGEDLFWAIRGGGGGSFGIVISWKIKLVHVPSTVTVFSVGRTLEQNATQLLHRWQYVAPNLPNDVYSVVTLSTTNGSENRTKTVLATFISLFQGGANDFIPLMQERFPELGLVKEDFIEMTWIESLLLLNGASNETSEILLDRSNRYSFLPPSFKSKSDYVREPMPEIALQGLWPQLLEVDEGGIAVQNIFAYGGIMEEISETETPFPHRKGTLYKIYYNIGWLEEENNNSQRYISWMRKLYSYMGPFVSKSPREAYVNYRDLDIGRNNDDGKASYKQASIWGRKYFKNNFDRLVYVKTKTDPKNFFKHEQSIPPRFH

>GrCKX12

MKSLHHSLSLLFIVVCSLSWVSASANSHDDFLECLYSYHPKESSSITQVIFTESNSSYSAVLDSSIRNHRFSTPNTPKPLVIVTPLNISHVQATIHCSKKQGLQIRTRSGGHDFEGLSYVSHVPFVVIDLVNLRSVDVDVENEEAWVQSGATVGEVYYRINERSTNLTFPAAVARTVGIGGSISGGGDGILFRKYGLSADNVIDAQLVDANGRILDRRSMGEDLFWAIRGGGGGSFGIVISWKIKLVHVPSTVTVFSVGRTLEQNATQLLHRWQYVAPNLPNDVYSVVTLSTTNGSENRTKTVLATFISLFQGGANDFIPLMQERFPELGLVKEDFIEMTWIESLLLLNGASNETSEILLDRSNRYSFLPPSFKSKSDYVREPMPEIALQGLWPQLLEVDEGGIAVQNIFAYGGIMEEISETETPFPHRKGTLYKIYYNIGWLEEENNNSQRYISWMRKLYSYMGPFVSKSPREAYVNYRDLDIGRNNDDGKASYKQASIWGRKYFKNNFDRLVYVKTKTDPKNFFKHEQSIPPGFH

>GrCKX13

MKSLHHSLSLLFIVVCSLSWVSASANSHDDFLECLYSYHPKESSSITQVIYTETNSSYSAVLDSSIRNHRFSTPNTPKPLVIVTPLNISHVQATIHCSKKQGLQIRTRSGGHDFEGLSYVSHVPFVVIDLVNLRSVDVDVENEEAWVQSGATVGEVYYRINERSTNLTFPAAVGRTVGIGGSISGGGDGILFRKYGLSADNVINAQLVDANGRVLDRRSMGEDLFWAIRGGGGGSFGIVISWKIKLVHVPSTVTVFSVGRTLEQNATQLLHRWQYVAPNLPNEYTQSLRYQRRTPVKIGQRRFLPPLFHCGANDFIPLMQERFPELGLVKEDFIEMTWIESLLLLNGASNETSEILLDRSNRYSILPPSFKSKSDYHYLQGLWPQQLEVDEGGIALQNIFAYGGIMEEISETETPFPHRKGTLYKIYYNIGWLEEENNNSQRYISWMRKLYSYMGPFVSKSPREVYVNYRDLDIGRNNDDGKASYKQASIWGRKYFKNNFDRLVYVKTKTDPKNFFKHEQSIPPRFH

>GrCKX14

MKSLHRSLSLLFIVACSLSWVSASANSHDDFLECLYSYHPKESSSITQVIYTETNSSYSALLDSSIRNQRFSTPNTPKPLVIVTPLNISHVQATIYCSKKHGLEIRTRSGGHDFEGLSYVSHVPFVVIDLVNLRSVDVDVENEEAWVQSGATVGEVYYRINERSTNLSFPAAVFRTVGIGGSISGGGDGPLFRKYGLSADNVIDAQLVDANGRVLDRRSMGEDLFWAIRGGGGGSFGIVISWKIKLVHVPSTVTFCSVGRTLEQNATQLLHRWQYVAPNLPNDVYSVVTISTTNASENGTKTVLATFISLFQGGANEFIPLMQERFPELGLVKEDFIEMTWVESLLLMNGLSNETSEILLDRSNRYSILPTFVKSKSDYVREPMPEIALQGLWLHLLQVDEGGIAVQNFFAYGGIMEEISETETPFPHRKGTLYKISYIIAWQEEENNNSQRYISWMRKLYSYMGPFVSKSPRAVYVNYRDLDIGRNNYHGKTSYKQASIWERKYFKNIFHRLVYVKTKTDPKNFFKHEQSIPPRIHHTS

>GrCKX15

MKSLHRSLSLLFIVACSLLWVSASANSHDDFFECLYSYHPKESSSITQVIYTETNSSYSALLDSSIRNQRFSTPNTPKPLVIITPLNISHVQATIHCSKKHGLEIRTRSGGHDFEGLSYVSHVPFVVIDLVNLRSVDVDVENEEAWVQLGATVGEVYYIINERSTNLSFPAPVFRTVGIGGSISGGGDGSLFRKYGLSADNVIDAQLVDAHGRVLDRRSMGEDLFWAIRGGGGGSFGIVISWKIKLVHVPSTVTFCSVGRTLEQNATQLLHRWQYVAPNLPKDVYSVVTISTTNASENGTKTVLATFISLFQGGANEFSLLMQERFPELGLVKEDFIEMTWVESLLLMNGLSNETSEILLDRSNRYTILPPFVKSKSDYVREPMPEIALQGLWLHLLEVDEGGIAVQNFFAYGGIMEEISETETPFPHRKGTLYKISYNIAWQEEENNNSQRYISWMRKLYSYMGPFVSKSPREAYVNYRDIDIGRNNYHGKTSYKQASIWGRKYFKNNFDRLVYVKTKTDPKNFFKHEQSIPPCLHCTS

>GrCKX16

MKSLHRSLSLLFIVVCSLSWVSASANSHDDFLECLYSYHPNESSSITQVIYTETNSSYLALLNSSIRNLRFSTPNTPKPLVIVTPLNISHVQATIYCSKKHGLEIRTRSGGHDFEGLSFVSPVPFVVIDLVNLRSVDVDVENEEAWVQSGASVGEVYYRINERSTNLSFPAAVFRTVGIGGSISGGGDGSLFRKYGLSADNVIDAQLVDANGRVLDRRSMGEDLFWAIRGGGGGSFGIVISWKIKLVHVPSNVTFFSVGRTLEQNATQLLHRWQYVAPNLPNDVYPVVTISTTNASENGTKTVLATFISLFQGGADEFFPLMQERFPELGLVKEDFIEMTWIESLLLMNGLSNETSEILLDRSNRYTILPPFAKSKSDYVREPMPEIALQGLWLHLLEVDEGGTAVLNFFAHGGIMEEISETETPYPHRKGTLYKISYVIAWQEEENNNSQRYISWMRKLYSYMGPFVSKSPREAYVNYRDLDIGSNNYYGKTSYKQASNWGRKYFKNNFDRLVYVKTKTDPKNFFKHEQSIPPLLHYTS

>GrCKX17

MEATSDSSSQVDKFLQCLANSSALMLESIYTPSNSSFESALQAYIRNRRFLTPETPKPVAIVAPNHVSHVQATVICAKDNGVQIRVRSVGHDYEGLSYRSNVTFVILDMLNLPSIDVDVDNEVAFVQASATIGELYYKIANESKGHAYPAGVCPSLGTGGHFSGGGYGNMMRKYGLSVDNILDAQVVGADGNILDRASMGEDLFWAIRGGGGASFAVIVSWKIKLVQVPEKITVFTVPFTLEQGATDVAFKWQQVAPKLPEDLYIRQIASVINGSCPLEKKILVSFMGLFLGEADKLVPLVNQSFPELNLTKEDCKEMSWLESTVYWAGFPVGTPVQVLLNRTQGAPNIFKVKSDYVKTVIPNEGLETIWQMLIGMGNTSAANISMQWNPYGGKMSEIPESSTAFPHRSGNLFLIHNRNIDRLRDMYDRMGPFVSKDPREAFLNYRDIDIGSSPSDQTNFTHAQVYGS

>GrCKX18

MAFWRISICSLLLLLLSSSTMATASIYRPRKRPTSSLIQDKFIQCFIGSSHFYIPLSTAFFTPNNASFTSVLQSTAQNLRYLVPSMPKPEFIITPQHESQVQASVICAKRLDIHLRFRSGGHDYEGLSYVSQIESPFVIVDLSKLRSIKVDIQDNSAWVEAGATIGEVYYRIAEKSNIHGFPAGLCTSLGIGGHITGGAYGSMMRKFGLGADNVIDARIVDVNGRVLDRAAMGEDLFWAIRGGGGASFGIILEWKIKLVPVPATVTVFTVTKSLEQGATKLLYKWQTVADKLDEDLFIRVIIQTDNAGKNNAKTVTTSYNALFLGNAERLLRVMQQSFPELGLTEKDCTETSWIKSVLYIAGYSSNTPAEILLQGRSTFKNYFKAKSDFVKEAIPETALEGLWKRLLEEDSPLMIWNPYGGMMARISESQIPFPHRQGTKFKIQYLTLWQDEDNNASKHFDWIRRLYNYMAPYVSMFPRGAYVNYRDLDLGMNKNINTSFIEASLWGVRYFKDNFMRLVKVKSRVDPNNFFRHEQSIPPLPVQARY

>GrCKX19

MTHSPPLSPSPSPSPFPFLFPFPFSWATNQGPFSIPPSSSLNIHSFGEYCAQFEGLILVRNPNQGEIEKSAFSNAFRDSFYHYKSQLNTCLKNLPSTIAGKTNSHAFSWSNCLLPLAFAISAGSLTFQSHNNHPSLYEPSNLDSHKTGNFLHRSTSSIPYGGATSIEGHTLSPNGGVCIDMTLMKRVKALHIWDMDVVAEPGIGWMELNEYLEPYGLFFPLDPGPGATIGGMCATRCSGSLAMM

>GrCKX20

MGSPVCAFLKQNNIIFLRFFAILVLSCIPDGTNLCSNPSFDTPTIPPHSSSSSIPSSLKTLTLDGYFSFENLKHAAKDFGNICHYLPIAVLHPKSVSDISSTIKHILYMSSVTKLTVAARGRGHSLQGQAQAYQGVVINMESLDRPSMYIENGEVPYVDVSGSELWINILHETLKYGLSPKSWTDYLHLTVGGTLSNAGISGQAFRHGPQIDNVYQLEVVTGRGEVVTCSDKENADLFYGVLGGLGQFGIITRARISLGPAEKMVKWIRVLYSEFSTFSNDQEHLISSNNSFDYIEGFVMINRTGLLNNWRSSFNPKDPIQASQFSSDGKILYCLEMVKYFNPEKIDVLNQDIEKLLSELNYIPSTLFLSEVSYVEFLDRVHLSEIKLRSKGLWEVPHPWLNLLIPKSRILDFAEGVFGNIVKDNNNGPILIYPVNKAKWNNRTSMVTPEEDIIYLVAFLSSALPGTDGLEHIMTQNQHILDFCAKAQLGAKQYLPHYHTQDEWQAHFGTQWETFVQRKSAYDPLAILAPGQRIFQKAISIT

>GrCKX21

MGFSFWLARLRSCSNKSAFSNAFRDSFYHYKSQLNTCQKNLPSTIAEKTNSHAFSWSSCLLPLAFAVSAGSLTFQSHNNHPSLCEPSNLDSRKVTIGGKASTEFVVKGTHKEVPQELIDELKAICQDNMTLDYDERFYHGKPQNSFHKAVNIPDVVVFPRSEEEVSQIVKSCNKHKVPIVPYGGATSIEGHTLSPNGGVCIDMTLMKRVKALHIRDMDVVVEPGIGWMELNEYLEPYGLFSPLIQVRPGATIGGMCATRCSGSLAVRYGTMRDNVISLKVVLANGDIVKTASRARKSAAGYDLTRLMIGSEGTLGVVTEVTLRLQKIPEHSVVAMCNFPTIKAAADVAIDTMLSGIQVSRVELLDEVQVRAINIANGKNLPEVPTLMFEFIGTKAYSHEQTQIVQRIVSEHNGSDFVFAEDPEAKKELWKIRKEALWACFAMEPNFEAMISDVCVPLSNLAELISRSKQELDASSLVCTVIAHAGDGNFHTVILFDPNEEEHRREAERLNQFMVYTALSMEGTCTGEHGVGTGKMKYLEKELGIEALQTMKRIKTALDPNNIMNPGKLIPPHVCF

>GrCKX22

MKFPQFSMLPFLMVVVMLSLSGTTLSHPHWDFLHCLSLRLANYSTISKVIYVQNNPSYSSVLNASIHNTRFSTPATPKPFAIITPLKTSHVRSTLYCSKKHGFQLRIRSGGHDVEGLSYVSEVPFVILDLLNFRAIKVDTKNEVAWVQSGATLGELYYAIAAKTNTLAFPAGMCHTVGAGGHFSGGGYGTLFRKYGLAADNIIDARMIDVNGRILDRKSMGEDVFWAIRGGGGNTFGIVLAWKIKLVPVPAVVTVFTVNKNLEQNATKIFHRWQYIAHKLPDDLFMNVWITKVNSSQVGMKTVQATFRGMLLGGVDELITLIQDKFPELGLAEENCNEMSWIESVLYFGGLPLQSVDILLDKNALPTLSLKAKSDYVKEPIPESGIEGLMSMFLEEEADNAVAMIPAFGGKMDDIPENELPYPHRAGNLFEATYIVQWTKEENAESGKYISWSRRLYSYMTPYVSKSPREAYVNYRDLDLGINNNDYTSYEQASIWGFKYFKNNFKRLVQVKTKVDPMNFFRNEQSIPPLLSPWKKKGN

>GrCKX23

MKFLQYSVLPFLLVVVILSLSGATLVHSHGDFLHCLSLHLPNSSTISKVIYMRNNPSYSSVLNASIQNTRFSTPATPKPLAIITPLKTSHIQSTIYCSKKHGLQLRIRSGGHDFEGLSYVSEVPFVILDLLNFRAVEVDTKNKVAWVQSGATLGELYYGIAAKTKTLGFPAGVCPTVGIGGHFSGGGYGILVRKYGLAADHIIDAQLIDANGRILDRKSMGEDLFWAIRGGGGNTFGIVLAWKIKLVPVPVVVTVFTVNKNLEQNVTKILHRWQYIAHKLPDDLYVDVGITKVSSSQAGKKTVQAAFIALFLGGVDELIPLVQERFPELGLAKENCSEMSWAESVLYLGRAPRTSLDMLLDKNVIPRTIFKAKSDYVKEPIPESGIEGFLSMFLEKEADFASMLMVPFGGKMEEIPENELPYPHRAGNLFQASYIVGWRKEENAECGKYISWIRRFYSYMATYVSKSPREAYFNYRDLDIGSNNISGYTSYQQASIWGFKYFKNNFKRLVQVKTMVDPMNFFRNEQSIPPLSSP

>GrCKX24

MKSLQLSMLLLSLVLAALLSFSMGALPHEDFLQCLSLRSNDSSTISSVIYTRNNPSYSTVLESTIRNLRFNSTNTPKPLVIVTPSRTSHFQATIYCSRKHGLQIRTRSGGHDYEGLSYVAKVPFVVVDLVNFRSVDVDVENRVAWVQAGAILGEVYYRIAEKSRTLSFAGGVFHSIGVGGFISGGGFGLLFRKYGTGGDNVIDAQFIDVNGRILDRKSMGEDLFWAIRGGGGGSFGIVLAWKLKLVPVPATVTVFSVSRTLEQNATQLILRWQEIAHQLPDEMNPDVSMFSVNSTQDGRKTIIASFSSLFLGTIDELLPIMQQRFPELGLSRQDCSEMSWIESILYFNQLQNQPLEILLNRTFRNPVGGQYYKIKSDYVKEPISETALNGLFSRLSDEEASSAIIIFMAYGGIMDRIPEDATPFPHRAGNLYKIYYNVNWQEQDNVNSQKYIDWSRRVYNYMTPFVSKSPREAYANYRDLDIGSNNVGITSYTQASVWGRKYFKNNFDRLVQVKTKIDPENFFKHEQSIPPLH

>GrCKX25

MFRSLSLKRSFKTHLKTLIHCPQHPPPPPPSSPVLFSSIRALSTASSPPSSSSDSELRKYLGYTALLAFCGVATYYSFPFSENAKHKKAQLFRYAPLPEDLHTVSNWSGTHEVQTRHFHQPENLKQLEELVKESNEKRVKLRPVGSGLSPNGIGLARGGMVNLALMDKVLEVDKEKKRVKVQAGIRVQQLVDEIKDYGLTLQNFASIREQQIGGILQVGAHGTGAKLPPIDEQIISMKLVTPAKGTIELSKEKDPELFYLARCGLGGLGVVAEVTIQCVERQELVEHTTVSNLKDLKKNHKKMLSENKHVKYLYIPYTDTVVVVTCNPVSKWRGPPKFKPKHTTDEAMQDIRELYKESLKKYRARDITTKSSDSNEPNINDFSFTELRDKLLSLDPLNKDHVMKVNHAEAEFWRKSEGYRVGWSDDILGFDCGGQQWVSETCFPAGTLSKPSMKDLEYIEELKKLIETNELPAPAPIEQRWTARSQSPMSPASSSAEDDIFSWVGIIMYLPTMDARQRKEITEEFFHYRHLTQSQLWDKYSAYEHWAKIEVPKDKEELEALQARLKTRFPVDAYNKARRELDPNRILSNNILEKLFPLSDNV

>GrCKX26

MPIIPVLYSNKLNALVLLKCLFLLVVLVGCSPPGEPVKCSTKDSNCTVTNSYGAFPDRTVCRAGNVVYPTSEQELVFIVSAATEAQRKMKVVTHFSHSIPKLVCPDGQDGLLISTKNLNRVVKINLAAMTMTVESGVTLRQLINEAAKAGLALPYAPYWWGLTIGGLLGTEAHGSSLWEKGSSVHDYVVEMRIVSPANAEDGYANLWVLNESDKDLDAAKVSLGVLGVISQPH

>GrCKX27

MPAIPMLFSNKFNVLLLWKCLCLLVVLVGCNPPGEPVKCSTKDSNCTVTNSYGMFPDRAICRAGNVAYPTSEQELVSIVSAATKSKRKMKVVTHFSHSIPKLVCPDGQDGLLVSTKNLNRVLKTDTEAMTMTVESGVTLRQLIKEAAKAGLALPYAPYWWGLTIGGLLGTGAHGSSLWGKGSSVHDYVVEMRIVSPAKAEDGYAKVWVLNERDKDLDAAKVSLGVLGVISQVTFKLQPLFKRSITYVRKDDTDLGDEAVTFGKLHEFADIFWYPSQRKAIYRIDDRVPINVSGNGVYNFTPFRSTLSLVLALVRSSEETQESSGDAEGKCLNAKLVTSTLQSSVYGLTNNGAIFTGYPVIGFHNRLQSSGTCLDSLEDSLITACPWDPRIKGEFFHQTTFSIGLSVVKSFIQDVQKLVSMDPKSLCGLELYNGILMRYVKASTAYLGKQEDAIDFDITYYRSKDPMAPRLYQDVLEEIEQMALLKYNALPHWGKNRNLVFDGVMKRYKNGGEFLKVKNKYDPWGLFSSEWTDQVLGLRNGVIVLKEGCALEGLCICSQDVHCAPSKGYLCKPGKTFPDARVCARVNTKT

>GrCKX28

MFPSLFVILLSVSWKSSSTLAYPSEGFLHCLSLHFANSTSISKLVYTQRNSSFSSVLKSSAQNFRFTTPSTPTPLFIITPLQPSHIQAAIYCSRIHGLHVRTRSGGHDFEGLSYTTANYIVPFVVIDLVNLRSVQVDVEKATAWVESGATIGELYYGIAQKSRTLAFPAGLFYTVGVGGQFSGGGYGPLFRKYGLAADNVIDARLIDAKGRILDRKSMGEDLFWAIRGGGGGSFGIVLAWKLKLVAVPAIVTVFTVSKTLEQNATKLVHRWQSIAHKFPKELLMSIFISSVKSSEADKKMTIKANFSSMFLGSIDELVPLMEERFPEFGLVRDDCLEMSWVEAILSTQGGVQLETLLERHQKTGVSQTFFKAKFDCVKQPIPEMGLEGLWPMFYEEEAKMANIFLVAYGGKMDEIPETEIPFPHRAGIIYSIIYVVDWDENGNKNSKRFLNWIRRVYDYMTPYVSNSPREAYVNYKDLQIGSNNMFSCKGSYAQAKIWGRKYFKNNFDRLVYVKTKVDPENFFRHEQSIPPLSCF

>GrCKX29

MTPYVSKSPREAYSNYRDLDIEANKINNDKESYAEARVWGLKYFKNNFDRISASAQPAEEFLRCLSLRFHNSSSIAKLVYTYHNSSYSSVLKSSAQNFRFTTPSTPTPLAIVTPFHASHIQATVYCCRKHGLQVRTRSGGHDFEGLSYTTAYKVPFVVIDLVNLRSVQVNVEKATAWVESGATVGELYYEIARKTRTLAFPAGIGHTVGIGGQLSGGGYGVLFRKYGLASDNVIDARLIDAYGRILDKKSMGEDLFWAIRGGGGGSFGIVLAWKVKLVPVPANVTACTVSKTLEQNATKLVHQWQSIARKFPKEIQSSIAIARVNSSEDGKMTIQASYGSVFLGSIDELIPLMEEKFPELGLVKEDCLEMSWAESILYSALSIIGLPLETLLNRTQKSALSQTFFKAKSDFVKQPISESGFEGLWPKFYEDEAKSAVMVLVAYGGKMDEIPETEYPYPHRAGNLYSILYVVNWEEEENKNSEKFMNWMRRVYSYMTPYVSKSPREAYVNYRDLDIGTNKFNHDKGSYTEAKVWGLKYFKNNFDKLVHVKTKVDPQNFFKHEQSIPPLF

>GrCKX30

MTLPTNLSIINNVIACNTFQCSHNIDPEIKLNMFPFLLLILLSLTWRISASAQPAEEFLRCLSLRFHNSSSIAKLVYTFTTPTPTPLAIVTPFHASHIQATVYCCRKHGLQVRTRSGGHDFEGLSYTTAYKVPFVVIDLVNLRSVQVNVEKATAWVESGATVGELYYEIARKTRTLAFPAGIGHTVGIGGQLSGGGLGPLFRKYGLASDNVIDARLIDAYARILDKKSMGKDLFWAIRGGGGGSFGIVLAWKLKLVLVPANVTACTVSKTLEQNATKLVHQWQSIARKFPKEIQSSIAIARVNSSEDGKMTIQASYGSVFLGSIDELIPLMEEKFPELGLVKEDCLEMSWAESILYGALSIIGLPLETLLNRTQKSALSQTFFKAKSDFVKQPISESGFEGLWPKFYEDEAKSAVMVLVAYGGKMDEIPETEYPYPHRAGYLYSILYVVNWEEEENKNSEKFLNWMRRVYSYMTPYVSKSPREAYVNYRDLDIGTNKFNHDKGSYTEAKVWGLKYFKSNYDKLVYVKTKVDPQNFFKHEQSIPPLF

>GrCKX31

MIAYLGRIVHNNDAESKLDDDVSSISKSLDLQGSIENGDVSSLASKDFGGLYSVKPLFLIKPSGAEDISRVVKLASRTSNLTVAARGNGHSINGQAMAEGGLVIDMCSTEKNHFEFLPINGSHYIDVSGGALWEDVLTRCVSRYGYAPRSWTDYLSLTVGGTLSNAGVSGQAFRYGPQTSNVTELEVVTGKGEITVCSETLNSELFFGVLGGLGQFGIITRARIKLQQAPDMVRWIRVVYSEFEEFTRDAEFLVTQKEGESFDYVEGFVFCNNDDPFNGWPSVPLDPGHEFNPTHISQTAGSVLYCLEVAFHYRNSDHPTVDTAVNGLLGRLRFVEGLKSQVDVSYTKFLLRVNRAEEQVKANGTWDGPHPWLNLFVSKSDVVNFDRTVFKTMLKDGVGGPMLIYPLLRSKWDDRTSVVLPEGEIFYIVALLRFVPNGPSVEKSVAQNREIVNWCIKVGLDFKLYLPHYQSKEDWERHFGNRWSRFVERKASFDPMAILAPGQNIFRRDLPNIIIGREF

>GrCKX32

MRGLPILVSIILFSISLVTSKSYPNSVVDNFLQCLPKHYRNPSKPIAKAIYTPTHPSFQSVYELHTYNLRVLTQANATHKPMAIVAALHESHVQAAVICAKESGLQVRIRSGGHDYEGLSFSSSVPFVIIDLSNLRSIKIDMKTETAWVQAGATTGELYYRIAEKSKVHAFPAGVCTTLGIGGHFTGGGYGNMLRKFGLSIDNVVDAQLINAKGRILNRKSMGEDVFWAIRGGGGTSFGIILSWKIKLVRVPPKVTVFQVAKTLEQGATVLVHKWLQVSHKLDKDIFIRIMPVTVAGTGNGNSTVRVSFIGHYLGRTNRLLPLVNASFPELGLQRKDCTEMSWIESTLYWAGFPNGTSTDVLLNRVPNKVFFKTKSDYLKNVMPKAGLETLWKVMMEIGNMWMQMSPYGGRMAEISESETAFAHRAGTLYLVQYTAHWSEGSSEATKKYVELMRKLYAEMAPYVSTKPREVFLNYRDLDIGSNNTDFEAAKVYGAKYFKGNFQRLAEVKAKIDPHNFFKNEQSIPPFPSF

>GrCKX33

MATKLLLTFAICRLIVTVGLTLDPTELLLLGVDAQLSVDPTDVKAASLDFGLLTGAQPPLAVMHPASSQDVAQLVKAAYGSNFGFTVSARGHGHSINGQAQTANGVVVQMSGSIGGSGVASGRKPPHPRVWPQERFVDVWGGELWIDVLRSTLQHGLAPKSWTDYLYLSVGGTLSNAGISGQAFNHGPQISNVHELDVVTGKGELLTCSEEQNSEMFHAVLGGLGQFGIITRARISLEPAPQRVRWIRVLYSNFSTFTRDQEYLISLHEQPASQKFDYVEGFVIVDEGLINNWRSSFFSPHNPVKISSLEPNGGVLYCLEIAKNYHESTASTIDQEVESLLKKLNFIPASVFTTDMPYVDFLDRVHKAELKLRSKGLWEVPHPWLNLFVPKSKIADFDKGVFKGILGNKTSGPILIYPMNKNKWDHRSSAVTPDEDVFYLVALLRSALDNGEETHSLEYLTNQNRQILRFCDEAGITVKQYLPHYKTHQEWVDHFGNKWDRFYRLKMEFDPRHILASGQQIFTPTNMASWQ

>GrCKX34

MATKLLLTFAICRLIVTVGLTLEPTELLRLGLTVDPFDVESASADFGLMTKVEPLAVLRPSSAKDVAQFVKAAYESSHGFTVSARGHGHSINGQAQTGTGVVVQMSGVGSGGKPRVWGDDMYVDVWGGELWIDVLKSCLAYGLAPKSWTDYLYLSVGGTLSNAGISGQAFHHGPQISNVHELDVVTGKGELMTCSKEQNPELFHAVLGGLGQFGIITRARISLEPAPQRVRWIRVLYSNFSAFTKDQEYLISLHGNQKFDYVEGFVIVDEGLINNWRSSFFSPRNPVKISSLGSNNNGGVLYCLEITKNYHESTAETIDQEIEALLKKLNFIPTSVFTTDLPYVDFLDRVHKAELKLRSKGMWEVPHPWLNLFVPKSKIADFDRGVFKGILGNKTSGPILIYPMNKNKWDDKSSVVTPDEDVFYLVAFLRSALDNGEETQSLEYLTNQNRQILKFCDEDGMKVKQYLPHYKTQSEWKEHYGSKWDRFQRMKMKFDPRHILASGQNIFTPTFLSSSNMMVMDIPIPIKQKARESIDF

>GrCKX35

MAVALPSFLTAIMIMSRLMAFIGITRNNDMSSKLQALDIAPKLSYDPSAIESASQDFGHIVKAVPQAVFLPSSPWDIASLVNFSYRNSVPFIIAARGNSHSVNGQAMAKNGVVIDMTSMKNGNGTGIRIASDGSYADVGGQQFWIDVLNATLGLGLTPVSWTDYLYLTVGGTLSNAGISGQTFRYGPQISNVYEIDVITGTADFVTCSPNNNSDLFYAALGGLGQFGIITRARIPLEPAPKRVKWVRMLYTDFSDFTRDQELLISKNGRNDNKALNYLEGSLLLDQGSLDNWRSSFFQPQDQPKIISLITKFRIVYCLEIVKHYDGQTKTTVDKDLQQLLKGLSYLPGFMFEKDAKYEEFLNRVHSEELKLKAKGLWDVPHPWLNLFIPKSKISDFNDGVFKSIVLQRNITTGPVLVYPMNRKKWDDRMSAVIPDEEIFYTVGLLQSSGFDDWRTFEAQNKEILQFCEKAGIKVKQYLPHYTTKEGWVNHFGSKWSTFQKRKLQFDPKLLLSPGQRIFNNNE

>GrCKX36

MIACLGRIVHDSDAESKLDDDVSTIFNSLNLQGSIENGDVSGIASKDFGGLYSFKPLYLIRPSGAEDISRIVKAAAGTPHLTVAARGNGHSINGQAMADGGYVIDMRSTGENHFKLLTVNGSPCIDVSGGALWEDVLRRCVSRFGLAPRSWTDYLSLTVGGTLSNAGVSGQAFRYGPQTSNVTELEVVTGKGDITVCSETQNPELFFGALGGLGQFGIITRARVKLQPAPDMVRWIRVVYAEFEEFTRDAELLVSQKEDESFDYVEGFVFCNNDDPVNGWPSVQLNPDQEFNPAHLPQTAGPVLYCLELGFHYRNSDQPSTVDMAVSRLVRGLGFVDGLISQVDVSYMGFLLRVKRAEQDAKANGVWDNPHPWLNLFVSKSDIVDFDRTVFKKMVKNGIGGPMLIYPLLRSKWDSRTSVALPEGEIFYIVALLRFVPKGPSVEKKVAENREIVKWCIKEGLDFKLYLPHYRAKEDWKRHFGNQWTRLEKRKANFDPMAILAPGQRIFKRTNQ

>GrCKX37

MAFSSTMILPLLLVLLSAFSATSKSVQENFMQCLDANSEHPIPISAFCSQTNSSFTSVLNSTAQNLRYLMPLVPKPEFIFIPVYESHAKSAVICAKRLAIHLRFRSGGHDYEGLSYASEIETPFILIDLNQLRSINVDVDDNSAWVQAGATVGEVYYRISEKSKTHGFPAGLCSSLGIGGHITGGAYGSMMRKYGLGADNVLDARIVDVNGEILDRAAMGEDLFWAIRGGGGASFGVILAWKIKLVAVPETVTVFTVPKTLEQGATKILYRWQQVADKLDDDLFIRVVIQVTKTSLKGKRTVTTAYNALYLGDAERLLQVMDQSFPELGLARKDCIETSWIKSVLYIAGFPSETPPEVLLEGKSLFKNYFKAKSDFVQQPIPETALEKLWEMLLEEESPLMIWNPYGGMMANISDSAIPFPHRKGNLFKIQYVTSWYEGSKDATRKHMDWIKGLYDYMSAYVPTSPRGAYVNYRDLDLGMNHNNASYTEASVWGAMYFKGNFRRLVKIKSKVDPGNFFRHEQSIPVVLDRA

>GrCKX38

MAISWPLVVSLLLSISSLATSASNSDSVHEAFVQCLLDNSHPSHPISEAIFTPQSRSYATVLQSYVRNLRFNETYTPKPFLILTALYQSHIQAAIICAKKGNIQMKIRSGGHDYDGLSYVATVPFFVLDMFNLRSIDIDMETETVWVQSGAILGELSYRISELSKTHGFPAGVCPTVGVGGHFTGGGYGNMMRKYGLTVDNIVDAYFIDVNGRIHDRKSMGEDLFWAIRGGGAASFGVVLAYKIKLVRVPEIVTVFRVEKTLEDNATDIVDQWQHVASKLPKELFVRLVIDVVNSSTRTGGSTVRVSFISLFLGDSKTLVSIMNENLPLLGLSQSDCIETSWIRSVLFWTNITIDSPTDVLLNRTPSLSYLKRKSDYVKQPIPKTALEGIWEKMIELQPAQMIFNPYGGRMAEIASTATPFPHRAGNLWKIQYLANWNQGGAETAQRYIELTRKLHRYMTPFVSKNPREAFLNYRDIDLGVNHNDRGSYLEGRVYGIKYFKGNFNRLVHIKTKFDPTNFFRNEQSIPTLPH

>GrCKX39

MNSSSSKLFILSTSFLLSISSITSNSVLDNFLQCLPIHSNTSNPITNSIYVPNNSSFQYLYELRANNLRIISSSTISKPLAILTARHASHVQAAVICAKIHSIQLRIRSGGHDYEGLSYRSDIPFVILDLFNLRSIKINITSETAWVQAGATTGELYYKIAEKSKVHGFPSGVCTTLGIGGHFTGGGYGNMIPKYGLSIDNVIDACLIDVNGTIHNRKSMGEDVFWAIRGGGGTSFGVILSWKIKLVHVPRKVTVFKVQRTLDQGATDIAYSWQHIAPKLPKYLFIRLQPEPITIGNGNKTIRVSFIGQFLGRSRKLMNLMNEEFPELRLQQNDCIEMSWVESTLFWAGFTNGTSIDVLLNRVVENKVFFKTKSDYYKNVIPKQGLVMLWEMLMDIGNIFVQLNPHGGRMDEISETETAVHQRGGYLFKVQYTVYWSESDGGIGAAKRYVEMSRRLYGAMAQYASSDPREAFLNYRDLDIGCNESNDTDFGVAEVYGTKYFNNNFMRLARVKAMVDPENFFKNEQSIPPLPSSH

>GrCKX40

MPNPMRPYLLLSVVFFFNLYHSMAVPDPTHQALLQCLTQSIPTDTASSIIVSKSNPSYNSVLRAYIRNARFNTSSTPKPLIIITPLDESHVSAAVICSQKLGFQVKIRSGGHDYEGLSYVFDKPFFVLDMFNLRSITVNMADETAWVGAGATLGELYYNIWKNSKVHGFPAGVCPTVGVGGHLSGAGYGTLIRKYGLSVDHVVDAKLVDVNGKILDRKTMGEDLFWAIRGGGAASFGVVLSYKIKLVPVPETVTVFRIERLLTENATDITFKWQTIAPTTDENLFMRMLLQPVTRNKKKTARISVIALYLGDSDSLVSLLQKDFPELSIGKSNCNETTWIDSVLWWANFDLGTPPTALLDRDLNDAGFLKRKSDYVQTPIPKSGLESLWQKMIELGKVGMVFNAYGGRMDQIKPDETPFPHRAGNLYKIQYSVNWDQPGSEADKNFTTQAKLLHDFMTPFVSKNPRSAYFNYRDIDVGSTKKWSYEEGRVYGESYFNGNYERLVDVKTAVDANNFFRNEQSIPPRSSSILHSSYGSATCIIIAVHTCWYYIVFIFSLCSDLF

>GrCKX41

MGFQSLVIIFLLCISMANSDHLKQNKTILQCLTDHSIASPSISSVTFFPTDPSFTSTLQSYTRNLRFTSTTTPKPLFIVVPSHVSHIQASIICCKTHGLEMRIRSGGHDYDGLSYVSKAPFMILDLFNLRSVIVDNGTAWVESGATLGELFYAISQKSKIHGFPAGVCPTVGVGGHFSGGGYGNMMRKFGLSVDNVIDAKLVDVNGNVLDRESMGEDLFWAIKGGGGASFGIIISWKIKLVSVPEIVTVFKIEKTLEQGVTGIVHKWQYIADKIDPNLFIRVVLLPVNKKHLQSIKAKFIGLFLGNGQELSSLMNEAFPELGLSFDQCIEMSWIESILFWSNYPKGTSLDVLLDRQPQQEKYLKKKSDYVQEPISKENLEGIWNKMIELKRPALTLNPYGGKMSEISEFETPFPHRAGNIYKIQYSVTWKDDGVEASGRSLDQIRKLYDYMTPYVSKSPRSSYLNYRDVDIGINENGNASYSEGVIWGRKYFKGNFERLVQVKSKVDPGNFFRYEQSIPCLGSWKSITAE

>GrCKX42

MLNPKCFNFLTLFISLLSLPSPTISQSSSLTNFLHCLHYGSDPIVSQSIYIASNPAFQTILQARIKNRRFLNPETLKPVAIVVPTRIDHVQGTVICAKDNGLQVRIRSGGHDYEGLSYRSNVTFIILDMSNFRSIDIDVKTETAWVQSGATLGELYYHIANKTNMHGFPSGVCPTVGIGGHFSGGGYGNLMRKYGLSVDNILDIIAVDALGNVHDRASMGEDLFWAIRGGGAASFAVVVSYKIKLVRVPNKVTVFRKGFTLEQGPTDLVHKWQQVAPNINEEFFIKVKLEPSFINGNQTVTATFIGFFLGRREKLLPIISKTFPELNLTQQDCHEMRWVETTLFWAGFPIGTPIETLLNRTIWTPLFFKNKSDYVKNVIPKESLNKIWKMTMAMMNRNDINKTRFDLECSPYGGKMNVIPESNTPFPHRKGNLFLIQYAFSWTDEGNNVSFNNIKKLRKLYDGMAPYVSKDPRECFLNYRDLDIGSNRSNETSFDDAKIYGRKYFKDNYTRLTKVKASVDPNNFFKYEQSIPPIK

>GrCKX43

MKISSSIFPIISIFILLSISSVTPSDSDDFDDFFQCLPKQSDSSIPITDAILTPNNSSFQYIYQLRANNLRTFLSATSRPVAIITALHPSHAQAAVICAKRHDFQLRIRSGGHDYEGLSYTSDVPFVILDMFNLNSIDIDMSTETAWVQAGATTGELYYRIAEKSNVHGFPSGVCTTLGIGGHFSGGGYGFLIRKYGLSIDNVIDAQLIDANGRILNRKSMGEDVFWAIRGGGTTSFGIILSWRIKLVRVPPRVTVFTVQRTLEQGATELAYRWQQVAPKLPKDLFIRLQPEPINNGGNNKTVRVSFIGHFLGQADGLLRLMNVSFPELGLTRNDCLEMSWVESTLYWAGFSNGSSIDVLLDRVAVNKVFAKEKSDYYKAVIPKQGLETLWQVLMDIEDIFVQMNPYGGRMEEISDSETAFAHRAGNLFKVLYGIQWSESEGGVNATARYVELSRRLYNAMAPYASSNPREAFINYRDLDIGSNESDETDFEDAKEYGAKYFRNNFIRLADVKAKIDPKNFFKNEQSIPPLPSH

>GrCKX44

MKISSSIFSLISIFILLLISSATSSDFDDFIRCLPQQSNSSLPITDAILTPNNSTFQSIYQLRANNLRTFLSATSRPVAIITALHPSHAQAAVICAKRHDFQLRIRSGGHDFEGLSYTSDVPFVILDMFNLNSIDIDMSTETAWVQAGATTGELYYRIAEKSNVHGFPSGVCTTLGIGGHFSGGGYGFLMRKYGVSIDNVIDAQLIDANGRILNRKSMGEDVFWAIRGGGTTSFGIILSWRIKLVRVPPRVTVFTVQRTLEQGATELAYRWQQVAPKLPKDLFIRLQPEPINNGGNNKTVRVSFIGHFLGQADGLLRLMNVSFPELGLTRNDCLEMSWVESTLYWANFPNGTSIDVLLDRVQENRVFSKSKSDYYKALIPKQGLETLWQGLMDIEDIFVQMNPYGGRMEEISDSETAFAHRAGNLFMVLYGIQWSESDGGINATERYVEMSRRLYDAMAPYASSNPREAFLNYRDLDIGSNESDETDFEDAQEYGAKYFRNNFIRLANAKATIDPENFFKNEQSIPPLPH

>GrCKX45

MIASSYICFIVSIFVLLSISSAASYDPVDFDAFLQCLPKHSDHSVSIAGAILTPNNASFQSTYQLRANNLRILLSATSRPVAIITALHPSHAQAAVICAKRHGFQLRIRSGGHDYEGLSYISDVPFVILDMFNLKSIDIDMKTETAWVQAGATTGELYYSIAQKSDVHGFPSGVCTTLGIGGHFSGGGYGFLMRKYGLSIDNVIDAQLIDTNGRILDRKSMGEDVFWAIRGGGTTSFGIILSWRIKLVRVPPRVTIFTVQRTLEQGATELAYRWQQVAPKLPKDLFIRLQLVPINNGGNNKTVTVSFIGHFLGQADGLLRLMNVRFPELGLTRNDCSEMSWVESALNWAGFPNGTSIDVLLNRVQVDRVFYKTKSDYYKAVIPKQGLETLWQVLMDIEDIFVQFNPYGGRMEEISESETAFAHRRGNLFKAQYGIQWSESDGGINATGRYVEMSRRLYNAMAPYASSNPREAFFNYRDLDIGSNESGETDFEVAKEYGAKYFMNNLMRLASVKAKIDPENFFKNEQSIPPLPTPPSH

>GrCKX46

MTPPLSPPSLLPLLLVAFNICFSLAASNSVYESFVQCLKTRSNSSDNISDIVYSHSNATYETVLEQYIRNARFNTSSTPKPVIIITPLTESHVSAAVICSNNIGFQLRIRSGGHDFEGLSYVSDQPFFILDMFNLRSISINMADQSVWVQSGATLGELYYRIWEESKVYGFPAGVCPTVGVGGHISGAGYGNMVRKYGLSVDYVVDAKIVDVNGNILDRKAMGEDLFWAIRGGGGASFGVILAFNIKLVDVPETVTVFKLERTLEQNATDVVYKWQSVAPTTDDNLFMRMLVQPVTLNKQKTIKISIMALYLGDVNSVVPLLVEDFPELGLVTEDCFEMSWIESALWWASFGKGTSPTVLLDRESYHVKFMKRKSDYVKTPISKDGLQWLWKKMIELEEPGLVFNPYGGKMNEIKETETPFPHRAGNLFKIQYSINWKDMGIEADKRSRSLVNRLHSYMTSFVSKNPRSAYLNYRDLDIGITKNWSYQEGKVYGESYFNGNFERLVDVKTVVDPHNFFRNEQSIPPRTIKAWNEKNEGSIPPSTSKAWNKSKPYVMIILFMAIGHII

>GrCKX47

MEISKPLLVFFSLVFFNLSFSWAAPDPTYQSLLQCLSEIIPSPNVSAVIVSNNNPSFASILESRIHNARFNRTSTLKPTIIITPSDESHVSAAVICSQKVGFQLKIRSGGHDYEALSYTSDKPFFLLDMYNLRDVSVDIPDESAWVQTGATLGELYYHIWEKSNAHGFPAGVCPTVGVGGHIGGAGYGTMIRKYGLTTDYVIDAKIVDVNGKILDRKAMGEDLFWAIRGAGGTNFGVVTAYKIKLVKVPEKVTVFRVERFLDNNGTEVAFKWQTVGATTDPNLFTRMLLQPNMKDKQRTVKVTVMGLYLGDINGLLTLLNKDFPELRLNKENCTEMPWIDSVLWWANFDLGTPPNVLLDRNNTDTKFVKRKSDYVQTPIPRDGLESLWQKMVQNEKVGLTCNSYGGKMDEIDPKETAFPHRKGNLYKIQYSINWDDPSIEADIKYTTQAKAVHEFMTQFVSKNPRRAYLNYRDIDIGSAKTWSYEEGKVYGESYFAENFDRLVDVKTAVDPNNFFRNEQSIPPRSTKTA

>GrCKX48

MSISMAISLLFSLLFLNISISSAASNPTYQSLLQCLSQSINPSQNVSTILFSNTNPSYASVLQAYIRNARFNTSSTPKPVIIITPLEESHVSAAVICSQKVGFQLKIRSGGHDYEGLSYVSDEPFFVLDMFNLRSISIDMTDESAWVETGATLGELYYNIWEKSNVHGFPAGLCPTVGVGGHLSGAGYGTLMRKYGLSSDYIVDAKIVNVDGKILDRKAMGEDLFWAIRGGGAASFGVVLAYKVKLVRVPETVTVFRLERLLADNATDIALKWQSIAPTTDENLFTRMLLQPVTRNRQRTMRVTVNGLYLGNADGVVALLSKDFPELGLKNENCTEMRWIDSVLWWANFDAGTPPTALLDRNVNDADFLKRKSDYVQTPISKNGLESLWQKMVELGNVGLACNAYGGRMDEIDDKETPFPHRKGNLYKIQYSVNWNEPGNETEMNRTSQAKALHEFMTQFVSKNPRRAYLNYRDIDIGVAENWSYEEGKVYGESYFAGNYERLVDVKTAVDPNNFFRNEQSIPPRTK

>GrCKX49

MEKWRATSNLRRSLKSILNRQLSSVSEFRYLNEKRSCQSSFNLIRDCKSLGQVNAIQHRCFSSASTLVQRNPSFSTLNSDDISYFKGLLGEKSVIQDEDRLETVNTDWMHKYKGSSKLLLLPRSTEEVAQILRYCNSRCLAVVPQGGNTGLVGGSVPVFDEVIVNISSMNNIISFDKVSGILVCEAGCILENLISFLDNQGFIMPLDLGAKGSCQIGGNVSTNAGGLRLVRYGSLHGNVLGLEAVLANGDVLDMLGTLRKDNTGYDLKHLFIGSEGSLGIVTKVSILTPPKLSSVNIAFLACNDYSSCQKLLMEAKRKLGEILSAFEFLDTEAMNLVLHQLDGVRNPLPASMHNFYILIETTGSDESYNREKLEAFLLSSMEGGLISDGVLAQDINQASSFWRIREGVPEALMKAGAVYKYDLSLPVEKMYDLVDDMRIRLGDLAKVVGYGHLGDGNLHLNVSAPEYDDKILEQIEPYVYEWTSKHRGSISAEHGLGLMKANKIYYSKSAETVSFPLNKKMQRISLFAGCVSPSHGCTSCLN

>GrCKX50

MQKHKMKSNLPIPLLFLSLLFSFSWAALGLTHQQQHHSFLQCLNRHFGNANSISTVIYTQTNSSFSSVLEFSLRNARFSTPNTLKPLVIVTASHVSHIQATINCSRTHGLQIRIRSGGHDYEGLSYVSQVPFVIIDLINLRSIDVDAENKTAWIQAGATIGELYYRIAEKSSTLAFPAGVCPTVGVGGHFSGGGYGMLMRKYGLAADQIIDAQLVDVNGKLLDKNSMGEDMFWAIRGGGAASFGVVVAWKVKLVPVPSTLTVFTVNRTLEENGTMLVHKWQSIAPKIHEDLYIRLFLRAVNSSQQEGKRTIQASFVSLYLGRADELIDLMQESFPELGLVKEDCIEMSWIQSIMYFPSDIPEDAPLEILLNRTGSAGIFKGKSDYVTQTIPETALEGLWQRFYEDETESLEILFSPYGGNMDDIPETETPYSHRAGNLFNIHYVVGWSEEDASESQRYINFMRRLYRYMEPYVSKSPRRAYMNYRDLDLGTNNIGPYTSYKQASKWGLPYFDKNFNRLIHVKTLVDPTNFFRYQQSIPSLSRG

>GrCKX51

MGKLKAVVVVTMISTALSILWRATLHLEDNESFVRCLLDHSHPSHPISSAIYTPKSSSFSSVLESYIRNLRFNESSTPKPFLILTALHESHIQAAVTCGKSHGVQMKIRSGGHDYEGLSYVSTLPFFLLDMFNLRCIDVDIETETAWVQTGATLGEVFYRIAEKSKTHGFPAGVCPTVGVGGHISGAGYGNMMRKYGVSADNVLDALIIDANGRLLDRQSMGEDLFWAIRGGGGASFAVVLAYKIKLVRVPETVTVFQVDRTLEEDATDIVDQWQHVAYNLPQELFIRLMLDVVVKSSGEKTLRASFVSLFLGDSESLLSIMKERFPKLGLSKSDCIETSWVKSVLFWSNIPLETDIQVLLDRTPQTLDYLKRKSDYVREPIPKAGLESLWKKMMELEKPRMYFNPYGGKMAEIAAEEIPFPHRAGNLWKIQYLANWNEAGIEAANRYIDLTRRLHEFMTPFVSKNPRQAFLNYRDADLGSSSHGKASYSEARLNGMKWFMGNFDRLVQIKTEVDPTNFFSYEQSIPLLPHQVHLDDDM

>GrCKX52

MKASQYFSMSLFLLILLISCPWLISANPHLNNFLGCLDSFYSNDISKVIYTQNNASYSSVLNATIQNLRFSTPTTSKPLVIVTPLQTSHIQATIRCSRTNGLNLRIRSGGHDFEGLSYVSQVPFVVLDLTNFRSVKIDVKNKVAWVQSGAILGEFYSEIAKRSRTLAFPAGICHTVGVGGYLSGGGYGLLLRKYGLAVDNVIDAVFIDVNGRILKRKSMGEDLFWAIRGGGGGSFGVVLSWKVKLVSVPSTVTVFTIRKTLEENATNLVHQWQSVGHKLPGDIFSAVTMRKVNRNGKPTILVAFSFFFLGETNALITLMKAGFPELGLKKEHCTEMSWIESILYFGQIQNKSIDVLLDRSYKSPLNAPWFKTKLDYVKNPIPKAGFEKIWSKLYEEDAETAAMAFIAYGGKMAEIPESATPFPHRDGNLYHIAYTVGWDGEENTKSQRYMNWIRKFYSFMTPFVSKSPRGAYVNYRDDDIGTNNKKGETSYAKASVWGRKYFKNNFDKLIYIKTKVDPHNFFKHEQSIPVSV

>GrCKX53

MLVSFPGQNNMLVLRSFMILFFSCITIKINLCFPSIPSSLKTLSIDGHFDFEQVEHAAKDFGNRYSYLPLAVLYPKSVSDVATTVNHVWQIGGGSELKVAARGHGHSLQGQAQAHRGVVINMESLQGLKMQVHTGNFPYVDVSGGELWINILRQSLKHGLAPKSWTDYLHLTVGGTLSNAGISGQAFRHGPQISNVYQLEVVTGKGEVVNCSEKQNSDLFYSVLGGLGQFGIITRARISLEPAPEMVKWIRMVYTDFATFTRDQEKLISGQSTFDYVEGFVIINRTGLLNNWRSSFNPQDPLQASQFKSDGRTLFCLELAKYFNHEDMVLVNQEIKTSLSQLNHIPSTLFISEVPYMEFLDRVHISEIKLRSRGLWEVPHPWLNLFIPKSKIHSFAQEVFGNILTDTSSGPILIYPVNKSKWDNRTSVVIPEEDVFYLVAFLSSAVPSSTGNDGLEQILIRNKRILDYCEIAGLGVKQYLPHFSTQGEWKSHFGPHWEAFIRRKSTYDPSAILAPGQRIFQKPIAYS

>GrCKX54

MLCLYIRDLLVFLCTSSVLAIELLFLLRELRAGDLHVMKDKIMLVLLEVSIKVWISVAGILYKDMLRVKALHIRDMDVVVEPGIGWMELNEYLEPYGLFFPLDPWPGATIGGMCATRCSGSLAMM

>GrCKX55

MHFLRNLIILFLICIAIKINLCVPTIPSSLKTLPIDGHFDFKQVHHAAKDFGNRYSFLPSAVLHPKSASDIATTVKHIWEMGPGSHLTVAARGHGHSLQGQAQAHRGVVINMESLQGPKMKVHTGNFPYVDVSGSELWINILHETLKHGLAPKSWTDYLHLTVGGTLSNAGISGQAFRHGPQISNVRQLEVVTGKGEVVNCSEKQNSDLFYSVLGGLGQFGIITRARISLEPAPKMVKWIRVLYTDFATFAKDQEMLISGESTFDYIEGFVIINRTGLLNNWRSSFNSNDSAQTSHFKSDGRTLFCLELAKYFNPEEMAIVNQEIMTSLSQLNHIPSTLFQSQVPYIEFLDRVHISEIKLRSKGLWEVPHPWLNLLIPRSNIHIFAQQVFGNILTNTSNGPILIYPVNKSKWDNRTSVVLPEEDVFYLVAFLSSAAPSSTGSDGLEHILNQNKRILELCEIDGLGVKQYLPHYSTNGEWRSHFGPQWEAFVHRKSTYDPLAILAPGQRIFQKAAPLSL

>GrCKX56

MAMVSSAHSYGDFLHCLSLRISNSSTISKVIYTQNNPSYSSVLNASIHNARFSTPTTPKPYAIITPRKTSHVQSTIYCSKNHGFQLRIRSGGHDVEGVSYVAQVPFVILDLVNFRDVNVDTKNEVAWVQSGATTGELYYGIASKTQTLGFPAGICHTIGIGGHLSGGGFGVLGRKYGLAVDHVIDAKLVDANGRVLRRKSMGEDLFWAIRGGGGNTFGVVLAWKVKLVPVPPVVTVFTVNKNLEQNATKIFHRWQYIAHKLPKDLFTTVWVMKVNSSQVGKKTVQASFRGMLLGGVDELIPLIQNEFPELGLAKENCTQMSWVQSILYFGGLPIQPVDILLNRHALPVSSVKEKTDFVREPMSETGIEGFMNMFLEEEADFAITMIEAFGGRMDEIRENELPYPHRAGILFESTYIVQWTNEADAGTYINWIRRLYSYMASYASKSPREAYYNYKDLDLGTNNIIGYTSYEQASVWGLKYFKNNFKRLVQVKTKVDPMNFFRNEQSIPPL

>GrCKX57

MALVSSEHEIPSTFCASISYCDIVIEWGNFSSSLWRFPSLPFSRISNSSTISKVIYTQNNPSYSSVLNASIHNSRFSTPTTPKPYAIITPLKTSHVQSTIYCSKNHGFQLRIRSGGHDVEGVSYVSQVPFVILDLVNFRDVKVDTKNEVAWVQSGATTGELYYGIASKTQTLGFPAGICHTIGIGGHLSGGGFGILGRKYGLAADHIIDAKMIDANGRVFHRKSMGEDLFWAIRGGGGNTFGVVVAWKIKLVAVPPVVTVFTVNKNLEQNATKIFHRWQNIAHKLPKDLFTSVWVMKVNSSQVGKKTVQASFKGMFLGRIDVLIPLIQYSFPELGLARENCTEMSWVQSVLYYGALPIEPVEILLNRSALPRLSLKAKTDYIREPMSETGIEGFMNMFLEEGTDFAITMIEAFGGKMDEIRESEIPFPHRSGILFESVYIVQWANEEDAGLCINWMRRLYSYMSSYASKSLRGAYYNYKDLDLGTNNINGYTSYEQASVWGLKYFRNNFKRLVRIKTMIDPMNFFSNEQSIPPLLSP

>GrCKX58

MKRNMLFLRSFMILVLSCIAIKINLCFPNILSSLKTLPIDGHFNFEQLHHAAKDFGNRYSFLPLAVLHPNSVSDIATTVKHIWQMGPGSDLTVAARGHGHSLQGQAQAHGGIVINMKSLQGLKMQFHIGNLPYVDVSGGELWINILREGLKHGLAPKSWTDYLHLTVGGTLSNAGISGQAFRHGPQISNVHQLEVVTGKGEVVTCSGKQNSDLFHGVLGGLGQFGIITRARISLEPAPEMVKWIRVLYTDFATFIRDQEELISGESTFDYVEGFVIINRTGLLNNWRSSFNPQDPVQASKFKSDGRTLFCLELAKYFNRDETAVVNREIHSSLSQLNHIPSTLFVSEVPYIEFLDRVHISEIKLRSKGLWEVPHPWLNLLVPRSKIQTFAQQVFGNILTDTSNGPILIYPVNKSKWDNRTSVVTPDEDVFYLVAFLSSAVPSSTGTDGLDHILIQNKRILEFCEIARLGVKQYLPHYSTQGEWKAHFGSRWEVFVRRKSSYDPLAILAPGQRIFQKAVPYSQ

>GrCKX59

MVGKITRAYEREVKEGDVKMASPKFGMSILFFFLCISFCSSTDQSFQQCFSSHLPPSNITYDVIFTQNSSQYSSILQSSIRNLRFSNASKPRYLVTPYNEDHIQATIICSKEHHMHVRVRSGGHDYEGLSYISDVPFIVIDLFHIRSVMVDIKNEYAWVGAGATLGELYYSISAKSNVHGFPAGSCPTVGVGGHISGGGFGTIFRKYGLAADNVIDAKMIDVNGNVLDRKSMGEDLFWAIRGGGGASFGVIFSWKLKLVRVPPTVTVFKTVKSLEQGATKLVQKWQNIAYKFHHDLFVHAVIQVTNPNSNQNPTVQVSFDCLFLGTTERLLSSIQRSFPELGVTQENCTEMSWIQSVLYFAGYSIAESADVLLNRTTQSTQSFKGKSDYVKEAIPKTGLEGLFKMVVEEETSVLILTPYGGRMKQIKSSATPFPYRSEYLYGIQYMISWDVAEETGKRIGWMRRLYKYMEPYVSTAPRAAYFNYRDLDLGRNSYPNTSYVESSEWGLKYFNHNFNRLVRVKTLADPHNFFWNEQSIPVLRFE

>GrCKX60

MSDLQAPLRPKRKKGLVDFLVQFRWIFVIFFVLPFSTLYYFLIYLGDVRSEMKSYKQRQKEHDENVLKVVKRLKQRNPKKDGLVCTARKPWIAVGMRNVDYKRARHYEVDLSAFRNILEIDKQRMIARVEPLVNMGQITRVTVPMNLSLAVVAELDDLTVGGLINGYGIEGSSHIYGLFSDTVVAYEIVLADGRVVRATKDNEYSDLFYAIPWSQGTLGFLVAAEIKLIPVKEYMRLTYTPVVGNLQDLAQGYMDSFAPRDGDQDNPEKVPDFVEGMVYSPTEGVFMTGRYASKEEAKKKGNKINNVGWWFKPWFYQHAQTALKKGEFVEYIPTREYYHRHTRCLYWEGKLILPFGDQWWFRFLLGWLMPPKVSLLKATQGESIRNYYHEMHVIQDMLVPLYKVGDALEWVHHEMEIYPIWLCPHRLFKLPVKTMVYPEPGFEQHRRQGDTPYAQMFTDVGVYYAPGPVLRGEVFDGAEAVRNLEQWLIKNHSFQPQYAVSELNEKDFWRMFDADLYEHVRRKYGAVGTFMSVYYKSKKGRKTEKEVQEAEQAHLETAYAEAD

>GrCKX61

MIACLGRIVQDTDADSIPDDDVSTLSESLDLQGTIESGGITGVAGKDFGGLYSVKPLALIKPSGTEDIARVVNAASRTSHLTVAARGNGHSINGQAMADGGFVIDMRSTEENHFKPLTIDGSHYIDVSGGALWEDVLKRCVSMFRLAPRSWTDYLSLTVGGTLSNAGVSGQAFRFGPQTSNVTELEVVTGKGEITVCSETQNSELFFGALGGLGQLGIITRARVKLQRAPDMVRWIRVVYTEFEEFARDAEFLVTQEESESFDYVEGFVFSNSDDPINGWPSVPLDPDHEFNPAYIPQTAGSVLYCLEVALHYRNSDRPSTVDTAVSRLLERLGSIQRLKFQLNVSYVEFLLRVKQVEEHAKANGNWDSPHPWLNIFISKSSIVDFDRTVFRKMLKDGVGGPMLIYPLLRSKWDSRTSVVLPEGEIFYIVALLRFVPKGPTVEKLVAQNHEIIKWCNKEGLDFKLYLPHYQSKEDWKRHFGNQWTRFVERKTNFDPMAILAPGQKIFKRTHIIKP

>GrCKX62

MVMSFQFPAYFTAIFIITRVMSIMKISKPLDVHHKDIRAVDLATKLSVDPSAIESASRDFGGIVKAEPEAVLHPSAPQDIAALIKFSYSSSVPFGIAAKGHGHSVRGQAMAENGVVVDMRSMANNRRNGTGIRVSIDRLYADVGGEQLWIDVLNATLEYGVAPVSWTDYLYLTVGGTLSNAGISGQTFRYGPQISNVLEMDVITGKADFLTCSPRMNSELFYAVLGGLGQFGIITRARIPLQPAPKGVKWVRLLYDDFSSFTKDQELLISKNGRKDKSALDYLEGSLLMDQGSPDNWRSSFFPHKDHPKIISLITKHGIIYCLEIVKHYDDRTKHTVDKGMKQVLQGLNYMPGFMFGKDVGYEEFLNRVRSGELKLKSQGLWDVPHPWLNLFIPKSQISDFNNGVFRGIVLERNITTGPVLVYPMNRQKWDDRMSAVIPDEEIFYTVGFLHSSGFDTWEAFEDQNKDIMRFCNKTGILVKQYLPHYSTKEEWVHHFGSKWKVFQHRKYQFDPRMLLSPGQRIFNNN

>GhCKX01A

MATMLLLTFLISSLIMVTVGLAIDFMEILRLGIDGQLSVDPSDVETASLDFGLLTRGQPLAVLHPVSAQDIARVVKAAYGSNQGMTVSARGHGHSINGQAQTTNGVVIQMSGWKGGNKPPRPHVWAEERYVDVWGGELWIDVLKSTLEYGLAPKSWTDYLYLSVGGTLSNAGISGQAFNHGPQISNVYELDVVTGKGEVLTCSEDENTELFHAVLGGLGQFGIITRARISLEPAPNMVRWIRVLYSNFSAFTSDQEHLISLHAEPSNQKFDYVEGFVIVAEGLINNWRSSLFSPQNPVKISTLFPTSGVLYCLEIAKNYHESTAQTIDQEVEFLLKKLNFISTSVFTTDLLYVDFLDRVHMAEMKLRSKGLWEVPHPWLNLFIPSSKIAEFDKGVFKGILGNKTSGPILIYPMNKNKWDHRSSVVTPDEGLFYLVALLRSALDSGEETQSLEYLNNQNRQILRYCDEAGIKVKRYLPHYTTQQEWMDHFGNKWDRFYEMKMEFDPRHILASGQRIFTPTFPSLSNMPS

>GhCKX02A

MNKETSYFKCSQNSSVSEKMKSIYCSLPFLFVVVCSLSWVNASANSHENFLDCLSSYHPQESSSISRVICTETNSSYSEILESSIQNYRFFTTNTPKPLVIVTPLNISHVQATIHCSKKHGLQIRIRSGGHDFEGLSYVSQVPFVVFDLVHFRSIDVDVENEEAWIQSGAITGEVYYRINERSTNLTFPGAVGHTVGIGGFISGGGYGLLFRKYGLAADNVIDAMFVDANGRILDRKSMGEDLFWAIRGGGGGSFGIVLSWKVKLVHVPSTVTVAAVRRTLEQNATQLLHRWQYVAPNLPNDVYSVVSISTTNSSENGERTVVATFVSVFQGGANEFIPLMQERFPELGLVREDYIEMTWIESILLLTGLTNQTSEVLLDRSYKNFFLSPSFKGKSDYMRKPMPEIVIQGLWSRLLEDEARISTLNIIAYGGKMDEIPETEIPFPHRKGTLYKISYYVGWQEEDNSNPQRYISWIRKVYKYMGPFVSKYPREAYLNYRDLDIGRNNNEGKASYKQASVWGRKYFKENFDRLTYVKAKIDPENFFRHEQSIPPRFH

>GhCKX03A

MESLNRSLSLLFIVVCSLSWVSASANSHDDFLECLYSYHPKESSSITQVIYTETNSSYSAVLDSSIRNHRFSMPNTPKPLVIVIPLNISHVQATIHCSKKHGLQIRTRSGGHDFEGLSYVSHVPFVVIDLVNLRSVDVDVENEEAWVQSGATVGEVFYRINERSTNLTFAAAVVRTVGIGGLISGGGDGLLFRKYGLSVDNVIDAQLVDANGRVLDRRSMGEDLFWAIRGGGGGSFGIVISWKIKLVHVPSTVTVFSVGRTLEQNATQLLHRWQYVAPNLPNDVYSLVAISTTNASENGAKTVLATFTSLFQGDANEFIPLMQERFPELGLVKEDFIEMTWIESLLLMNGVSNETSEILLDRSNRYSLLPPSFKSKSDYVREPMPEIALQGLWPQLLEVDEGGIAVQNFIAYGGIMEEISETETPFPHRKGTLYKINYNIGWLEEENNNSQRYISWMRKLYSYMGPFVSKSPREAYVNYRDLDIGSNNYYGKTSYKQASIWGRKYFKNNFDRLVYVKTKTDPKNFFKHEQSIPPRFH

>GhCKX04A

MATASIYRLRKRPTSSLIQEKFIQCFTGSSQFYIPLSTAFFTPNNASFTSVLQSTSQNLRYLVPSMPKPELIITPLHESQAQASVICAKRLDIHLRVRSGGHDYEGLSYVSQIESPFVIVDLSKLRLIKVDIQDNSAWVEAGATIGEVYYRIAEKSNIHGFPGGLCTSLGIGGHITGGAYGSMMRKFGLGADNVIDARIVDVNGRVLDRAAMGEDLFWAIRGGGGASFGIILEWKIKLVPIPATVTVFTVTKSLEQGATKLLYKWQTVADKLDEDLFIRVIIQTANAGKNNAKTVTTSYNALFLGDAERLLRVMQQSFPELGLTRKECTETSWIKSVLYIAGYSSNTPAEILLQGRSTFKNYFKAKSDFVKEAIPETALEGLWKRLLEEDSPLMIWNPYGGMMGRISESQIPFPHRQGIKFKIQYLTLWQVEDNNASKHFDWIRRLYNYMAPYVSMFPRGAYVNYRDLDLGMNKNINTSFIEASLWGVRYFKDNFMRLVKVKTRVDPNNFFRHEQSIPPLPVQARY

>GhCKX05A

MKDSNSATLPLVTIVLFLSLSWRATSDSSSQVDKFLQCLANSSALMLESIYTPSNSSFESALQAYIRNHRFLTPETPKPVAIVAPTHVSHVQATVVCAKDNGVQIRVRSGGHDYEGLSYRSNVTFVILDMFNLRSIDVDVDNEVAFVQAGATIGELYYKIANESKDHAYPAGVCLSLGTGGHFNSGGYGNMMRKYGLSVDNILDAQVVGADGNILDQASMGEDLFWAIRGGGGASFAVIVS

>GhCKX06A

MRNVDYKRARHFQVDLAFRNILEIDKVQMIARVEPIVTKGQSITVPMNLSLAVVADLDYLTIFGLINGNGIAGNSHIYGLFSYTVVAYEKVLAVALLELQTMMNILISSMLPLGLKELLAGADGGPDP

>GhCKX07A

MKVLQLSVLSFLIVILSLNGATLAHPYGDFLHCLSLRISNSSTISKVIYTQNNPSYSSVLNASIHNTRFATPTTPKPYAIITPLKTSHVQSTIYCSKNHGFQLRIRSGGHDVEGVSYVSQVPFVVLDLVNFRDVKVDTKNEVAWVQSGATTGELYYGIASKTQTLGFPAGICHTIGIGGHLSGGGFGILGRKYGLAADHVIDVKLVDANGRVLHRKSMGEELFWAIRGGGGNTFGVVLAWKVKLVPVPPVVTVFTVNKNLEQNATKIFHRWQYIAHKLPNELFTAVWVMKVNSSQVGKKTVQAGFRGMFLGGVHELIPLIQHEFPELGLAKENCTQMSWVQSILYFGGLPIQPVNILLNRSALPVSSLKAKTDFVREPMSETGIEGFMNMFLEEEADFAITMIEAFGGRMDEIRENELPYPHRAGILFESTYIVQWTNEAEAGRYISWIRRLYSYMASYASKSPREAYYNYKDLDLGTNNIVGYTSYEQASVWGLKYFKNNFKRLVQIKTKVDPMNFFRNEQSTPPL

>GhCKX08A

MKFLQFSVLPFLMVILSVSGANLTHHPHRDFLRCLSLRIENSCTITYTHNNPSYPSVLNASIQNTRFSTPTTPKPYAIITPRKTSDVQSTIFCSKNHGFQLRIRSGGHDVEGVSYVSQVPFVVLDLVNFRDVKVDTKNEVAWVQSGATTGELYYGIAAKTQTLGFPAGICHTIGIGGHLSGGGFGILGRKYGLAADHIIDAKMVDANGRVLHRKSMGEDLFWAIRGGGGNTFGVVLAWKIKLVPVPPVVTVFTVNKNLEQNATKIFHRWQQIAHKLPNDLFTTVWVMKVNSSQVGKKTVEASFKGLFLGRIDELIPLIQYAFPELGLARENCTQMSWVQSVLYFGALPIEPVEILLNRSALPRLSLKAKTDYIREPMSEAGFEGFMNMFLEEGTDLAITMMEAFGGKMDEIRENEIPFPHRSGVLFESVYIVQWINEEDAGLCINWMRRLYNYMSSYASKSLREAYYNYKDLDLGINNVNGYTSYEQASVWGLKYFKNNFKRLVRVKTMIDPTNFFSNEQSIPPLLSP

>GhCKX09A

MIAYLGRIVHDNDAESKLDDDVSSISKSLDLQGSIENGDVSSLASKDFGGLYSVKPLFLIKPSGAEDISRVVKLASRTSNLTVAARGNGHSINGQAMAEGGLVIDMRSTEKNHFEFLPINGSHYIDVSGGALWEDVLTRCVSRYGYAPRSWTDYLSLTVGGTLSNAGVSGQAFRYGPQTSNVTELEVVTGKGEITVCSETLNSELFFGVLGGLGQFGVITRARIKLQQAPDMVRWIRVVYSEFEEFTRDAEFLVTQKEGESFDYVEGFVFCNNDDPFNGWPSVPLDSGHEFNPTHISQTAGSILYCLEVAFHYRNSDHPTVDTAVNGLLGRLRFVEGLKSQVDVSYTKFLLRVNRAEEQVKANGPGDGPHPWLNLFVSKSDVVNFDRTVFKTMLKDGVGGPMLIYPLLRSKWDDRTSVVLPEGEIFYIVALLRFVPNGPSVEKLVAQNREIVNWCIKVGLDFKLYLPHYQSKGDWERHFGNRWSRFVERKASFDPMAILAPGQNIFRRDPSNIIISREF

>GhCKX10A

MATKLLLTFAICRLIVTVGLTLDPTELLLLGVDAQLSVDPTDVKAASLDFGLLIGAQPPLAVMHPASSQDVAQLVKAAYGSNFGFTVSARGHGHSINGQAQTANGVVVQMSGSKGGSGMASGRKPPHPRVWPQERFVDVWGGELWIDVLRSTLQHGLAPKSWTDYLYLSVGGTLSNAGISGQAFNHGPQISNVHELDVVTGKGELLTCSEEQNSEMFHAVLGGLGQFGIITRARISLEPAPQRVRWIRVLYSNFSIFTSDQEYLISLHEQPASQKFDYVEGFVIVDEGLINNWRSSFFSPHNPVKISSLDPNGGVLYCLEIAKNYHESTASTVDQEVESLLKKLNFIPASVFTTDLPYVDFLDRVHKAELKLRSKGLWEVPHPWLNLFVPKSKIADFDKGVFKGILGNKTSGPILIYPMNKNKWDHRSSAVTPDEDVFYLVALLRSALDNGEETHSLEYLTNQNRQILRFCDEAGITVKQYLPHYTTHQEWVDHFGNKWDRFYRLKMEFDPRHILASGQQIFTPTNMASWR

>GhCKX11A

MATKLLLTFAICRLIVTVGLTLEPTELLRLGLTVDPVDVESASVDFGLMTKVEPLAVLRPSSAEDVAQLVKAAYESSHGFTVSARGHGHSINGQAQTGTGVVVQMSGVRSSGKPSVWGDDMYVDVWGGELWIDVLKSCLEYGLAPKSWTDYLYLSVGGTLSNAGISGQAFHHGPQISNVHELDVVTGKGELMTCSKEQNPELFHAVLGGLGQFGIITRARISLEPAPQRVRWIRVLYSNFSAFTKDQEYLISLHGNQKFDYVEGFVIVDEGLINNWRSSFFSPRNPVKISSLGSNNNGGVLYCLEITKNYHESTAETIDQEIEALLKKLNFIPTSVFTTDLPYVDFLDRVHKAELKLRSKGMWEVPHPWLNLFVPKSKIADFDRGVFKGILGNKTSGPILIYPMNKNKWDDKSSVVTPDEDVFYLVAFLRSALDNGEETQSLKYLTNQNRLILKFCDEDGIKVKQYLPHYKTQSEWKEHYGSKWDWFQRMKMKFDPRHILASGQNIFSPTFLSSSKMASW

>GhCKX12A

MAVALPSFFTAIMIMSRLMAFIGISKNNDMSSKLQALDIAPKVSHDPSAIESASQDFGHIVKAAPQAVLLPSSPRDIASLVNFSYSNSVPFSIAARGNSHSLNGQAMAKNGVVIDMTSMKSGNGTGIRIASDGSYVDVGGQQLWIDVLNATLGLGLTPVSWTDYLYLTVGGTLSNAGISGQTFRYGPQISNVYEIDVITGTADFVTCSPNNNSDLFYAALGGLGQFGIITRARIPLEPAPKRVKWVRMLYTDFLDFTRDQELLISKNGRNDNKALNYLEGSLLLDQGSLDNWRSSFFPPQDQPKIISLITKFRIVYCLEIVKHYDSQTKTTVDKDLQQLLKGLSYLPRFMFEKDAKYEEFLNRVHSEELKLKAKGLWDVPHPWVNLFIPKSKISDFNDGVFKSIVLKRNITTGPVLVYPMNRKKWDDRMSAVIPDEEIFYTVGLLQSSGFDDWRTFEDQNKEILQFCEKAGIKVKQYLPHYTTKVGWVNHFGSKWSTFQKRKLQFDPKLLLSPGQRIFNNNQ

>GhCKX13A

MIACLGRIVHDSDAESKLDDDVSTIFNSLNLQGSIENGDVSGIASKDFGGLYSVKPLYLIRPSGAEDISRVVKAAAGTPHLTVAARGNGHSINGQAMADGGYVIDMRSTGENHFKLLTVNGSPCIDVSGGALWEDVLRRCVSRFGLAPRSWTDYLSLTVGGTLSNAGVSGQAFRYGPQTSNVTELEVVTGKGDITVCSETQNPELFFGALGGLGQFGIMTRARVKLQLAPDMVRWIRVVYAEFEEFTRDAEFLVSQKEDESFDYVEGFVFCNNDDPVNGWPSVQLNPDQEFNPAHLPQTAGPVLYCLELAFHYRNSDLPSTVDMAVSRLVGGLGFVDGLISQVDVSYMGFLLRVKRAEQDAKANGVWDNPHPWLNLFVSKSDIVEFDQTVFKKMVKNGIGGPMLIYPLLRSKWDSRTSVALPEGEIFYIVALLRFVPKGPSVEKKVAENREIVKWCIKEGLDFKLYLPHYRAKEDWKRHFGNQWTRFEKRKANFDPMAILAPGQRIFKRTNQ

>GhCKX14A

MAFSSTMILPLLLVLLSAFSATSKSVQENFMQCLDANSKHPIPISAFCFQTNSSFTSVLNSTAQNLRYLMPLVPKPEFIFIPVYESHVKSAVICAKKLAIHLRFRSGGHDYEGLSYASEIETPFILLDLIQLGSINVDIDDNSAWVQAGATVGEVYYRISEKSKTHGFPAGLCSSLGIGGHITGGAYGSMMRKYGLGADNVLDARIVDVNGEILDRAAMGEDLFWAIRGGGGASFGVILSWKIKLVAVPETVTVFTVPKTLEQAATKILYRWQQVADKLDDDLFIRVVIQVTKTSQKGKRTVTTAYNALYLGDAERLLQVMDQSFPELGLARKDCIETSWIKSVLYIAGFPSETPPEVLLEGKSLFKNYFKAKSDFVQQPIPETALERLWEMLLEEESPLMIWNPYGGMMANISDSAIPFPHRRGNLFKIQYVTSWYEGSKGATRKHMDWIKGLYDYMSAYVPTSPRAAYVNYRDLDLGMNHKNASFTEASVWGAMYFKGNFRRLVKIKSKVDPGNFFRHEQSIPVVLE

>GhCKX15A

MAISWPLVVSVLLSISSLVTSASNSDSVHEAFVQCLLDNSHPSHPISEAIFTPQSPSYATVLQSYIRNLRFNETYTPKPFLILTALHQSHIQAAIICAKKGNIQMKIRSGGHDYDGLSYVATVPFFVLDMFNLRSIDIDTETETVWVQSGAILGELYYRISELSKTHGFPAGVCPTVGVGGHFTGGGYGNMMRKYGLTVDNIVDAYVIDVNGRIHDRKSMGEDLFWAIRGGGAASFGVVLAYKIKLVHVPEIVTVFRVEKTLEDNATDIVDQWQHVASKLPKELFVRLVIDVVNSSTRTGGSTVRVSFISLFLGDSKTLVSIMNENLPLLGLSQSDCIETSWIRSVLFWTNITIDSPTDVLLNRTPSLSYLKRKSDYVKQPIPKTALEGIWEKMIELQPAQMIFNPYGGRMAEIESTATPFPHRAGNLWKIQYLANWNQGGAETAQRYIGLTRKLHRYMTPFVSKNPREAFLNYRDIDLGVNHNDRGSYLEGRVYGIKYFKGNFNRLVHIKTKVDPTNFFRNEQSIPTLPH

>GhCKX16A

MPNPMRPYLLLSLVFFFNLYHSMAVSDPTHQALLQCLTQSIPTDTASSIIVSKSNPSYTSVLRAYIRNARFNTSSTPKPLIIITPLDESHVSAAVICSRKLGFQLKIRSGGHDYEGLSYVFDKPFFVLDMFNLRSITVNMADETAWVGAGATLGELYYNIWKNSKVHGFPAGVCPTVGVGGHLSGAGYGTLIRKYGLSVDHVVDAKLVDVKGKILDRKAMGEDLFWAIRGGGAASFGVVLSYKIKLVPVPKTVTVFRIERLLTENATDITFKWQTIAPTTDENLFMRMLLQPVTRNKKKTARITVIALYLGDSDSLVSLLQKDFPELSIGKSNCNETTWIDSVLWWANFDLGTPPTALLDRDLNDAGFLKRKSDYVQTPIPKSGLESLWQKMIELGKVGMVFNAYGGRMDQIKPAETPFPHRAGNLYKIQYSVNWDEPGNEADKNFTTQAKLLHDFMTPFVSKNPRSAYFNYRDIDVGSTKKWSYEEGKVYGESYFNGNYERLVDVKTAVDPNNFFRNEQSIPPRSSKI

>GhCKX17A

MAVSFPIPSYFTAIFIISRLMSITGISKHLNNKLLPPLDNITDKLSLDPSAIESASQDFGHIVKSIPKAVLQPSSIADIASLINFSYNSSIPFTIAAKGHGHSVRGQAMANDGVVVDMTSMKKHRNGTGIWVSNDGVYADVGGEQLWIDVLNATLKHGVAPVSWTDYLYLTVGGTLSNGGISGQSFRYGPQISNVYEMDVITGKIFSFSNVKWIRMLYNDFTAFTRDQELLISINGRHDSHALGYLEGSLLMDHGSPDNWRSSFFPPKHHPKITSSIINHRIIYCLEVVKHYDYQTQNTVDKELEQLLKGLSYMPGFIFEKDVLYAEFLNRVQRGELKARSQGLWDVPHPWLNLFIPKSQIQRFNDGVFKGIVLERNITTGPVLVYPMNRKKVNCFASPPSILGWDDRMSAVIPDEEIFYTVGFLHSSGFDDREAFDDQNKEILKFCEEAGIGVKQYLPHFTSKDEWVHHFGSKWETFQQRKFQFDPKMILSPGQRIFNNN

>GhCKX18A

MANLSLLLLHFLISSLSVSGSAATDQRNIMSCLNYYNISNYTISSNVHNHDYSILLNFSIQNLRFAEPTIPKPIAIILPENKEQLINTVVCCTIGPWEIRVRCGGHSYEGTSSVASDGAPFVIIDMMNLNRVSVDLGNETAWVEGGATLGETYHAIAESSDIHGFAAGSCPTVGTGGHIGGGGFGFLSRKYGLAADNVIDALLLNAEGELLDRQAMGEDVFWAIRGGGGGIWGIVYAWKIKLLKVPKTVTSFIVSRPGTIAHVANLVNKWQHVAPNLEGDMYLSCAVGAGLPQAKSIGISATFNGFFLGRKREAVLILGQVFAELDVSEEACKEMSWIESVLFFSGLGDGAIVSDLKNRYLHDKHYFKAKSDYVRNPISLTGIRTAIDILEKQPKGYIIMDPYGGIMNNISNDSIAFPHRYGNLYTIQYLVEWHQEEKNRSNEYREWIRDFYDAMASHVSWGPRAAYVNYMDFDLGVMELINTSVLSEDTIEMARVWGEKYFLNNYDRLVKAKTLIDPNNVFKNQQGIPPSTTTGLKARTF

>GhCKX19A

MVNLSLLLFLISSPSVSGSASPDQRNIMSCLNSYNISNYTISSNVHNHDYSILLNFSIQNLRFAEPTIPKPIAIILPENKEQLINTVVCCTKGPWEIRVRWVEGGATLGETYHAIADSSGIHGFSAGSCPTVGTGGHIGGGSFGFLSRKYGLAADNVIDALLLNAKGELLDRQAMGEDVFWAIRGGGGGIWGIVYAWKIKLLRVPKTVTSFIVSRPSTKAHVANLVNKWQHVAPNLEGDMYLSCAVGAGLPQAKSIGISATFNGFFLGRKRGAILILGRVFAELGVAEEACKEMSWIESVLFFSGLGDGAIVSDLKNRYLHDKHYFKAKSDYVRNPISLTGIRTAIDILEKQPKGYIIMDPYGGIMNNISNDSIAFPHRYGNLNTIQYLVEWHQEEKNRSNEYREWIRDFYDAMASHVSWGPRAAYVNYMDFGLGVMELINTSVLSEDTVEMARVWGEKYFLNNYDRLVKAKTLIDPNNVFKNQQGIPPSTTTGLKARTF

>GhCKX20A

MRIAYLDRTVHETDGEPKPNGDVSAISKSIDLQGSVETGDKTTIASKDFGGLYSIKPLALIKPAGSDDISRAIKAASRIPRMTVAARGNGHSINGQAMTNGGLVIDMRSTEENHFRLLNINGSFFIDVSGGALWENVLTRCVSRFGLAPRSWTDYLSLTVGGTLSNAGVSGQAFRYGPQISNVTELEIVTGKGDITVCSETRNPELFFGSLGGLGQFGIITRARVKLQPAPDMVRWIRVVYTEFDEFTRDAEFLVSRDDGESFDYVEGFVFCNNDDPVNGWPAVPLDPVHGFNPGIIPQTGASVLYCLEVAFHYQKVDHPSTVDKAVAGLLKRLRFVEGLKSQVDLSYVEFLLRVKRAEEQAKANGIWDAPHPWLNLFISKSNIVDFDQTVFKKMVKDGIGGPMLIYPLMRSKWDNRTSVALPDSEIFYLVALLPFVSRGPSVEESVAQNREIVEWCIKEGLDFKLYLPHYQSKEGWKRHFGDQWTRFVERKASFDPMAILAPGQNIFKRTHLS

>GhCKX21A

MGSPVCGFLKQNNIIFLRFFAILVLSCIPDRTNLCSNPSFDTPTIPPHSSSSSIPSSLKTLTLDGYFSFENLKHAAKDFGNICHYLPIAVLHPKSVSDISSTIKHILYMSSVTKLTVAARGRGHSLQGQAQAYQGVVINMESLDRPSMYIENGEVPYVDVSGGELWINILHETLKYGLSPKSWTDYLHLTVGGTLSNAGISGQAFRHGPQINNVYQLEVVTGTGEVVTCSDKENADLFYGVLGGLGQFGIITRARISLGPAEKMVKWIRVLYSEFSTFSNDQEHLISSNNSFDYIEGFVMINRTGLLNNWRSSFNPKDPIQASQFSSDGKILYCLEMVKYFNPEKIDVLNQDIEKLLSELNYIPSTLFLSEVSYVEFLDRVHLSEIKLRSKGLWEVPHPWLNLLIPKSRIFDFAEGVFGNIVKDNNNGPILIYPVNKAKWNNRTSMVTPEEDIIYLVAFLSSALPGTDGLEHIMTQNQHILDFCAKAELGAKQYLPHYHTQDEWRAHFGTQWETFVQRKSAYDPLAILAPGQRIFQKAISIT

>GhCKX22A

MGFSFWLACLRSCSNKSAFSKAFRDSFYHYKSQLNTCQKNLPSTIAEKTNSHAFSWSSCLLPLAFAVSAGSLTFQSHNNHPSLCEPSNLDSRKVTIGGKASTEFVVKGTHKEVPQELIDELKAICQDNMTLDYDERFYHGKPQNSFHKAVNIPDVVVFPRSEEEVSQIVKSCNKHKVPIVPYGGATSIEGHTLSPNGGVCIDMTLMKRVKALHIRDMDVVVEPGIGWMELNEYLEPYGLFFPLDPGPGATIGGMCATRCSGSLAVRYGTMRDNVISLKVVLANGIIVKTASRARKSAAGYDLTRLMIGSEGTLGVVTEVTLRLQKIPEHSVVAMCNFPTIKDAADVAIDTMLSGIQVSRVELLDEVQVRAVNIANGKNLPEVPTLMFEFIGTEAYSHEQTQIVQRIVSEHNGSDFVFTEDPEAKKELWKIRKEALWACFAMEPNFEAMISDVCVPLSNLAELISRSKQELDASSLVCTVIAHAGDGNFHTVILFDPNEEEHRREAERLNQFMVYTALSMEGTCTGEHGVGTGKMKYLEKELGIEALQTMKRIKTALDPNNIMNPGKLIPPHVCF

>GhCKX23A

MMKFPQFSMLPFLMVVVMLSLSGTTLSHPHWDFLHCLSLRLANYSTISKVIYVQNNPSYLSVLNASIHNTRFSTPATPKPFAIITPLKTSHVRSTLYCSKKHGFQLRIRSGGHDVEGLSYVSEVPFVILDLLNFRAIKVDTKNEVAWVQSGATLGELYYAIAAKTNTLAFPAGMCHTVGAGGHFSGGGYGTLFRKYGLAADNIIDARMIDVNGRILDRKSMGEDVFWAIRGGGGNTFGIVLAWKLKLVPVPAVVTVFTVNKNLEQNATKIFHRWQYIAHKLPDDLFMNVWITKVNSSQVGTKTVQATFRGMLLGGVDELITLIQDKFPELGLAEENCNEMSWIESVLYFGGLPLQSVDILLDKNALPTLSLKAKSDYVKEPIPESGIEGLMSMFLEEEADNAVAMIPAFGGKMDEIPENELPYPHRAGNLFEATYIVQWTKEQNAESGKYISWSRRLYSYMTPYVSKSPREAYVNYRDLDLGINNINDYTSYEQASIWGFKYFKNNFKRLVQVKTKVDPMNFFRNEQSIPPLLSPWKKKGN

>GhCKX24A

MLEDKHKMLNPKCVHFLTLLISLLSLPSPTISQSSSLTNFLHCLHYGSDPMVSQSIYIASNPAFQTILQARIKNRRFLNPETLKPVAIVVPTHIDHVQGTVICAKDNGLQIRIRSGGHDYEGLSYRSNVTFIILDMSNFRSIDIDIKTETAWVQSGATLGELYYHIANKTNTHGFPSGVCPTVGIGGHFSGGGYGNLMRKYGLSVDNILDIIAVDALGNVHDRASMGEDLFWAIRGGGAASFAVVVSYKIKLVRVPNKVTVFTKGFTLEQGATDLVHKWQQIAPNINEELFIRVKLQPSFINGNQTVTVTFIGFFLGRREKLLPIISKTFPELNLTQQDCHEMRWVETTLFWAGFPIGTPIETLLNRTIWTPLFFKNKSDYVKNVIPKESLNKIWKMTMAMMDRNDINKTRFDLECSPYGGKMNVIPESNTPFPHRKGNLFLIQYSFSWIDEGNNVSFNNIEKLRKLYDGMAPYVSKDPRECFLNYRDLDIGSSRSNETSFDDAKIYGRKYFKDNYTRLTKVKASVDPNDFFKYEQSIPPIN

>GhCKX25A

MAFTSISSLFLLLLVLHFSSSTTASTCHAKSFKLNPIQEKFIQCFKANSEIPIPVSTEFFTPNNASFSTVLQSTAQNLRYLEPPVPKPEFIIMPLNESHVQAAVICSKELGIHMRVRSGGHDYEGMSYVSETESPFILVDLSKLRSVKVDIENNTAWIQAGATIGEVYYRIYEKSKIHGFPAGLCTSLGVGGHITGGAYGSMMRKYGLGVDNVIDARIVDVNGRVLDRAAMGEDLFWAIRGGAGGSFGIILQWKIQLVPVPSTVTVFTITKSLQQNGTKIFHRWIEVADNLDDDLFIRVIIQTAMINGEKTVTTSYNSLFLGEADRLVEIMQQSFPELGLTRKDCIETSWIKSVLYIAGYPSNTPPEVLLQGKSTFKNYFKAKSDFVKSNIPETALEGLWKRFMEEDTPLMIWNPYGGMMARISESETPFPHRKGNKIMIQYVSAWQDGDKNESKHIDWIRRLYNYMAPYVSMFPRTAYVNYRDLDLGTNKNASTSFIEASGWGVKYFKDNFNKLVKVKTKVDPENFFRHEQSIPPLPVEARF

>GhCKX26A

MEKWRATSNLRRSLKSILNRQLSTVSEFRYLNEKRSCQSSFNLIRDCKSLGQVNAIQHRCFSSASTLVQRNPSFSTLNSDDISYFKGLLGEKSVIQDEDRLETVNTDWMHKYKGSSKLLLLPRSTEEVAQILRYCNSRCLAVVPQGGNTGLVGGSVPVFDEVIVNVSSMKNIISFDKVSGILVCEAGCILENLISFLDNQGFIMPLDLGAKGSCQIGGNVSTNAGGLRLVRYGSLHENVLGLEAVLANGDVLDMLGTLRKDNTGYDLKHLFIGSEGSLGIVTKVSILTPPKLSSVNIAFLACNDYSSCQKLLMEAKRKLGEILSAFEFLDTEAMNLVLHQFDGVRNPLPASMHNFYILIETTGSDESYNREKLEAFLLSSMEDGLISDGVLAQDINQASSFWRIREGVPEALMKAGAVYKYDLSLPVEKMYDLVDDMRIRLGDLAKVVGYGHLGDGNLHLNVSAPEYDDKILEQIEPYVYEWTSKHRGSISAEHGLGLMKANKVYYSKSAETVQTMASIKKLLDPNGILNPYKVLPHSLNS

>GhCKX27A

MSFFFPEMKASQCFSMSLFLLILLISCPWLISANPHLNNFLGCLDSFYSNDISKVIYTQNNASYSSVLNATIQNLRFSTPATPKPLVIVTPLQTSHIQATIRCSRTNGLNLRIRSGGHDFEGLSYVSQVPFVVLDLTNFRSVKIDVKNKVAWVQSGAILGEFYSEIARRSRTLAFPAGICHTVGVGGYLSGGGYGLLLRKYGLAVDNVIDAVFIDVNGRILKRKSMGEDLFWAIRGGGGGSFGVVLSWKVKLVSVPSTVTVFTIRKTLEENATNLVHQWQSVAHKLPGDIFSAVTMRKVNRSGKPTILVAFSSFFLGDTNALIPLMKARFPELGLKKEHCTEMSWIESILYFGQIQNKSIDVLLDRSYKSPLNAPSFKTKLDYVKNPIPKAGFEKIWSKLYEEDAETAAMAFIAYGGKMAEIPESATPFPHRDGNLYHIAYTVGWDGEENTKSQRYMNWIRKFYSFMTPFVSKSPREAYVSYRDDDIGTNNKKGETSYAKASVWGRKYFKNNFDKLIYIKTKVDPHNFFKHEQSIPVSV

>GhCKX28A

MGKLKAVVVVTMISTVLLSILWRATLHLEDNENFVRCLLDHSHPSHPISSAIYTPKSSSFSSVLESYIRNLRFNESSTPKPFLILTALHESHIQAAVTCGKSHGVQMKIRSGGHDYEGLSYVSTLPFFLLDMFNLRSIDVDIETETAWVQTGATLGEVFYRIAEKSKTHGFPAGVCPTVGVGGHISGAGYGNMMRKYGVSADNVLDALIIDANGRLLDRQSMGEDLFWAIRGGGGASFAVVLAYKIKLVRVPETVTVFQVDRTLEEDATDIVDQWQHVAYNLPQELFIRLMLDVVVKSSGEKTLRASFVSLFLGDSESLLSIMKERFPKLGLSKSDCIETSWVKSVLFWSNIPLETDIQVLLDRTPQTLDYLKRKSDYVREPIPKAGLESLWKKMMELETGI

>GhCKX29A

MQKHKMKSNIPIPLLFLSILFSFSWAALGLTHQQQHHSFLQCLNRHSGDANSISTVIYTQTNSSFSSVLEFSLRNARFSTPNTLKPLVIVTPSHVSHIQATINCSRTHGLQIRIRSGGHDYEGLSYVSQVPFVIIDLINLRSIDVDAENKTAWIQTGATIGELYYRIAEKSSTLAFPAGICPTVGVGGHFSGGGYGMLMRKYGLAADQIIDAQLVDVNGKFLDKNSMGEDLFWAIRGGGAASFGVVVAWKVKLVPVPSTLTVFTVNRTLKENGTMLVHKWQSVAPKIHEDLYIRLFLRAVNSSQQEGKRTIQASFVSLYLGRADELVDLIQESFPELGIVKEDCIEMSWIQSIMYFPSDIPEDAPLEILLNRTGSAGIFKGKSDYVTQTIPETALEGLWQRFYEDETESLEILFSPYGGNMDDIPETETPYSHRAGILFNIHYVVGWSEEDASESQRYINFMRRLYRYMEPYVSKSPRRAYMNYRDLDLGTNNNGPYTSYKQASKWGLPYFDKNFNRLIHVKTLVDPTNFFRHQQSIPSLSRG

>GhCKX30A

MLVSFPGQNNMLVLRSFMILFFSCITIKINLCFPSIPSSLKTLSIDGHFDFEQVEHAAKDFGNRYSYIPLAVLYPKSVSDIATTVNHVWQIGGGSELKIAARGHGHSLQGQAQAHRGVVINMESLQGHKMQVHTGNFPYVDVSGGELWINILRESLKHGLAPKSWTDYLHLTVGGTLSNAGISGQAFRHGPQISNVYQLEVVTGKGEVVNCSEKQNSDLFHSVLGGLGQFGIITRARISLEPAPEMVKWIRMVYTDFATFTRDQEKLISGKSTFDYVEGFVIINRTGLLNNWRSSFNPQDPLQASQFKSDGRTLFCLELAKYFNHEDMVLVNQEIKTSLSQLNHIPSTLFISEVPYMEFLDRVHISEIKLRSRGLWEVPHPWLNLFIPKSKIHSFAQEVFGNILTDTSSGPILIYPVNKSKWDNRTSVVIPEEDVFYLVAFLSSAVPSSTGNDGLEQILIRNKRILDFCEIAGLGVKQYLPHFSTQGEWKSHFGPHWEAFTRRKSTYDPSAILAPGQRIFQKLIAYS

>GhCKX31A

MGTSYHEDFLQCLSLRSNDSTSISNVVYTRNNSSYSSVLESTIRNLRFNSTDTPKPLVIVTPSRTSHFQATIYCARKHRLQIRTRSGGHDYEGLSYVAKVPFVLVDLVNFRSVDVDAENRVAWVQASAIVGGVYYTIGLGSFISNRGFGLLFRKYDTGGDNVIDAQLIDVNRRILDRKSMGEDLFWAI

>GhCKX32A

MKFLQFSMLLLPILLDALLSFSMGASTHQDFLQCLSLRSNDSTSISNVIYTRNNSSYSFVLESTIRNLRFNSTDTPKPLVIITPSRTSHFQATIYCARKHGLQIRTRSGGHDYEGLSYVARVPFVVVDLVNFRSVDVDVENRVAWVQAGAILGEVYYRIAEKSRTLGFAGGIYFTVGVGGHISGGGFGLLFRKYGLACDNVIDAQFIDVNGRILDRKSMGKDLFWAIRGGGGGSFGIVLAWKVALVPVPPTVTAFSISRTLEQNATQLILRWQDIAHQLPDEMNPDVTMFSFNSTQDGRKTILVSFSSLFLGTIDELLPIMQQRFPELGLSGQDCIEMSWIEAVLYYNQLQNQPLETLLNRTFRTPSGGQYYKIKSDYVKEPISETGLNGLFSRLSDEEASSAVIIFMAYGGIMGRIPEDATPYAHRAGNLFKIYYNVNWQEQDNVNSQKYIDWSRRVYKDMTPFVSKSPREAYANYRDLDLGSNNVGITSYTQASIWGRKYFKNNFDRLVQVKTKIDPENFFKHEQSIPPLF

>GhCKX33A

MKSLQFSMLLLSLVLAALLSFSTGALPQEDFLRCLSLRSNDSSTISSVIYTRNNPSYSTVLESTIRNLRFNSTNTPKPLVIVTPSRTSHFQATIYCSRKHGLQIRTRSGGHDYEGLSYVAKVPFVVVDLVNFRTVDVDVENRVAWVQAGAILGEIYYRIAEKSRTLAFAGGIFHSIGVGGYISGGGFGLLFRKYGTAGDNVIDAQFIDVNGRILDRKSMGEDLFWAIRGGGGGSFGIVLAWKLKLVPVPAIVTVFSVNRTLEQNATQLILRWQEIAHQLPDEMNPDVTMLSVNSTQDGRKTILASFSSLFLGTIDELLPIMQQRFPELGLSRQDCSEMSWIESVLYFNQLQNQPLEILLNRTFRTSVGGQYYKIKSDYVKEPISETALNGLFSRLSDEEASSAFIIFMAYGGIMDRIPEDATPFPHRAGNLYKIYYDVNWQEQDNVNSQKYIDWSRRVYNYMTPFVSKSPREAYANYRDLDIGSNNVGITSYRQASVWGRKYFKNNFDRLVQVKTKIDPQNFFKHEQSIPPLH

>GhCKX34A

MFRSLSLKRSFKTHLKTLIHCPQQPPPPPPPSPVLFSSIRALSTASSPPSSSSDSELRKYLGYTALLAFCGVATYYSFPFPENAKHKKAQLFRYSPLPEDLHTVSNWSGTHEVQTRHFHQPENLKQLEELVKESSEKRVKLRPVGSGLSPNGIGLARGGMVNLALMDKVLEVDKEKKRVRVQAGIRVQQLVDEIKDYGITLQNFASIREQQIGGILQVGAHGTGAKLPPIDEQIISMKLVTPAKGTIELSKEKDPELFYLARCGLGGLGVVAEVTIQCVERQELVEHTTVSNLKDLKKNHKKMLSENKHVKYLYIPYTDTVVVVTCNPVSKWRGPPKFKPKHTTDEAMQDIRELYKESLKKYRARDIKTKSSDSDEPNINEFSFTELRDKLLSLDPLNKDHVMKVNHAEAEFWRKSEGYRVGWSDDILGFDCGGQQWVSETCFPAGTLSKPSMKDLEYIEELKKLIETNELPAPAPIEQRWTARSQSPMSPASSSAEDDIFSWVGIIMYLPTMDARQRKEITEEFFHYRHLTQSQLWDKYSAYEHWAKIEVPKDKEELEALQARLKARFPVDAYNKARRELDPNRILSNNILEKLFPLSDTV

>GhCKX35A

MPAIPMLFSNKFNVLLLWKCLCLLVVLVGCNPPGEPVKCSTKDSNCTVTNSYGMFPDRAICRAGNVAYPTSEQELVSIVSAATKSKRKMKVVTHFSHSIPKLVCPDGQDGLLVSTKNLNRVLKTNTEAMTMTVESGVTLRQLITEAAKAGFALPYAPYWWGLTIGGLLGTGAHGSSLWGKGSSVHDYVVEMRIVSPAKAEDGYAKVWVLNERDKDLDAAKVSLGVLGVISQVTFKLQPLFKRSITYVRKDDTDLGDEAVTFGKLHEFADIFWYPSQRKAIYRIDDRVPINVSGNGVYNFTPFRSTLSLVLALVRSSEETQESSGDAEGKCLNAKLMTSTLQSSAYGLTNNGAIFTGYPVIGFHDRLQSSGTCLDSLEDSLITACPWDPRIKGEFFHQTTFSIGLSVVKSFIQDVQKLVSMDPKSLCGLELYNGILMRYVKASTAYLGKQEDAIDFDITYYRSKDPMAPRLYQDVLEEIEQMALFKYNALPHWGKNRNLLFDGVMKRYKNGGEFLKVKNKYDPWGLFSSEWTDQVLGLRNGVTILKEGCALEGLCVCSQDVHCAPSKGYLCKFGKIFSDARVCARVNTKT

>GhCKX36A

MQRNMLFLRSFMILVLSCIAIKINLCFPNILSSLKTLPIDGHFNFEQLHHAAKDFGNRYSFLPLAVLHPNSVSDIATTVKHIWQMGPGSDLTVAARGHGHSLQGQSQAHGGIVINMKSLQGLKMQFHIGNLPYVDVSGGELWINILREGLKHGLAPKSWTDYLHLTVGGTLSNAGISGQAFRHGPQISNVHQLEVVTGKGEVVTCSEKQNSDLFHGVLGGLGQFGIITRARISLEPAPEMVKWIRVLYTDFATFTRDQEELTSGESTFDYVEGFVIINRTGLLNNWRSSFNPQDPVQARKFKSDGRTLFCLELAKYFNRDETAVVNREIHSSLSQLNHIPSTLFVSEVPYIEFLDRVHISEIKLRSKGLWEVPHPWLNLLVPRSKIQTFAQQVFGNILTDTSNGPILIYPVNKSKWDNRTSVVTPDEDVFYLVAFLSSAVPSSTGNDGLDHILIQNKRILEFCEIARLGVKQYLPHYSTQGEWKAHFGSRWEVFVRRKSSYDPLAILAPGQRIFQKAVPYSQ

>GhCKX37A

MSDLQAPLRPKRKKGLVDFLVQFRWVFVIFFVLPFSALYYFLIYLGDVRSEMKSYKQRQKEHDENVKKVVKRLKQRNPKKDGLVCTARKPWIAVGMRNVDYKRARHYEVDLSAFRNILEIDRERMIARVEPLVNMGQITRVTVPMNLSLAVVAELDDLTVGGLINGYGIEGSSHIYGLFSDTVVAYEIVLADGRVVRATKDNEYSDLFYAIPWSQGTLGFLVAAEIKLIPVKEYMRLTYTPVVGNLQDLAQGYMDSFAPRDGDQDNPEKVPDFVEGMVYSPTEGVFMTGRYASKEEAKKKGNKINNVGWWFKPWFYQHAQTALKKGEFVEYIPTREYYHRHTRCLYWEGKLILPFGDQWWFRFLLGWLMPPKVSLLKATQGESIRNYYHEMHVIQDMLVPLYKVGDALEWVHHEMEIYPIWLCPHRLFKLPVKTMVYPEPGFEQQHRQGDTPYAQMFTDVGVYYAPGPVLRGEVFDGAEAVRKMEQWLIKNHSFQPQYAVSELNEKDFWRMFDADLYEHVRRKYGAVGTFMSVYYKSKKGRKTEKEVQEAEQAHLETAYAEAD

>GhCKX38A

MIVCLGRIVQDTDADSIPDDDVSTLSESLDLQGTIESGGITGVAGKDFGGLYSVKPLALIKPSGTEDIARVVKAASQTSHLTVAARGNGHSINGQAMADGGFVIDMRSTEENHFKPLTIDGSHYIDVSGGALWEDVLRRCVSMFRLAPRSWTDYLSLTVGGTLSNAGVSGQAFRFGPQTSNVTELEVVTGKGEITVCSATQNSELFFGALGGLGQLGIITRARVKLQPAPDMVRWLRVVYTEFEEFTRDAEFLVTQEEGESFDYVEGFVFSNSDDPINGWPSVPLDPDHEFNPAYIPQTASSVLYCLEVALHYRNSDRPSTVDTAVSRLLERLGFIQRLKFQLNVSYVEFLLRVKQVEEHAKANGNWDSPHPWLNIFISKSSIVDFDRTVFRKMLKDGVGGPMLIYPLLRSKWDSRTSVVLPEGEIFYIVALLRFVPKGPTVEKLVAQNHEIIKWCNKEGLDFKLYLPHYQSKEDWKRHFGNQWTRFVERKTSFDPMAILAPGQKIFKRTHTIKP

>GhCKX39A

MVMSFQFPAYFTAIFIITRVMSIMKISKPLDVHHKDIRAVDLATKLSVDPSAIESASRDFGGIVKAEPEAVLHPSAPQDIAALIKFSYSNSVPFGIAAKGHGHSVRGQAMAENGVVVDMRSMANKRRNGTGIRVSIDRLYADVGGEQLWIDVLNATLEYGLAPVSWTDYLYLTVGGTLSNAGISGQTFRYGPQISNVLEMDVITGKADFLTCSPRMNSELFYAVLGGLGQFGIITRARIPLQPAPKRVKWVRLLYDDFSSFTKDQELLISKNGRKDKSALDYLEGSLLMDQGSPDNWRSSFFPYKDHPKIISLITKHGIIYCLEIVKHYDDRTKHTVDKEMEQVLQGMNYMPGFIFGKDVGYEEFLNRVRSGELKLKSQGLWDVPHPWLNLFIPKSQISDFNNGVFRGIVLERNITTGPVLVYPMNRQK

>GhCKX40D

MATMLLLTFLISSLIMVTVGLAIDSMEILRLGIDGQLSVDPSDVETASLDFGLLTRGQPLAVLHPVSAQDISQVVKAVYGSNQGMTVSARGHGHSINGQAQTTNGVVIQMSGWKGGNKPPRPRVWAEERYVDVWGGELWIDVLKSTLEYGLAPKSWTDYLYLSVGGTLSNAGISGQAFNHGPQISNVYELDVVTGKGEVLTCSEDENTELFHAVLGGLGQFGIITRARISLEPAPKMVRWIRVLYSNFSAFTSDQEHLISLHAEPSNQKFDYVEGFVIVDEGLINNWRSSLLSPQNPVKISTLFPSGGVLYCLEIAKNYHESTVQTIDQEVEFLLKKLNFISTSVFTTDLLYVDFLDRVHKAELKLRWDHRSSVVTPDEGVFYLVALLRSALDNGEETQSLEYLNNQNRQILRYCDEAGIKVKRYLPHYTTQQEWMDHFGNKWDRFYEMKMEFDPRHILASGQRIFTPTFPSSSNMPS

>GhCKX41D

MKSLYCSLPFLFVVVCSLSWVNASANSHENFLDCLSSYHPQESSSISKVIYTETNSSYSEILESSIQNYRFFTTNTPKPLVIVTPLNNSHVQATIHCSKKHGLQIRIRSGGHNFEGLSYVSQVPFVVIDLVHFRSIDVDVENEEAWIQSGAITGEVYYRINERSTNLTFLGVVGHTVGISGFISGGGYGLLFRKYGLAAANVIDAMFVDANGRVLDRKLMGEDLFWAIRGGGGGSFGIVLSWKVKLVHVPSTVTVAAI

>GhCKX42D

MKSLHDCLPLLFVVVFSLSWVNASANSNDYFLDCLSSYHPDEFTSFSKVIYTETNSSYSAVLESSTRNPRFSTPNTPKPLVIVTPSNISHVQATIHCSKKHGLQIRTRSGGHDFEGLSYVSQVPFVVIDLVHFRSIDVDVKKEEAWIQSGAITGEVYYRINERTTNLTFPGALCHTVGIGGFISGGGYGFLFRKYGLAADNVIDAQFVDANGRVLHRRLMGEDLFWAIRGGGGGSFGIVLSWKVKLVHVPSTVTVFSVGRTLEQNATQLLHRWQYVAPNLPNDVYSLVSISSMNSTENGERTVLATFTSVFQGVADELIPLMQERFPELGLLKEDYIEMTWIESILFWNQLSNETSEILLDRSNRNSLVPLSYKSKSDYVRKPMPEIALQGLWSRLLEVNETSTAVVNIISYGGKMDEIPETETPFPHRKGTLYKINYNIVWQEEENSNPQRYISWMRTLYSYMGPFVSKSPRAAYVNYRDLDIGRNNDDGKTRYKQASVWGRKYFKNNFDRLVYVKTKIDPENFFKHEQSIPPRFH

>GhCKX43D

MKSLHRSLSLLFIVMAVLDSSIRNQRFSTPNTPKPLVIVTPLNISHVQATIHCSKKHGLQIRTRSGGHDFEGLSYVSHVPFVVIDLVNLRSVDVDVENEEAWVQSGATVGEVYYRINERSTNLTFPAAVGRTVGIGGSISGGGDGLLFRKYGLSADNVIDAQLVDANGRVLDRRSMGEDLFWAIRGGGGGSFGIVISWKIKLVHVPSTVTVFSVGRTLEQNATQLLHRWQYVAPNLPNDVYSVVTISTTNASENRTKTVLATFISLFQGGANDFIPLMQERFPELGHVKEDFIEMTWIESLLLLNGASNETSEILLDRSNRYSILPPSFKSKSDYVRKPMPEIALQGLWPQLLEVDEGGIGVHYFIAYGGIMEEISETETPFPHRKGTLYKIYYNIGWLEEESNNSQRYISWMRKLYSYMGPFISKSPREVYLNYRDLDIGRNNYYGKTSYKQASIWGRKYFKNNFDRLVYVKTKTDPKNFFKHEQSIPPRFH

>GhCKX44D

MKFLHRSLSLLFIVVCSLSWVTASANSHDDFLECLYSYHPNESSSITQVIYTETNSSYLALLNSSIRNQRFSTPNTPKPLVIVTPLNISHVQATIYCSKKHGLEIRTRSGGHDFEGLSFVSPVPFAVIDLVNLRSVDVDVENEEAWVQSGATVGEVYYRINERSTNLSFPAAVFRTVGIGGSISGGGDGSLFRKYGLSADNVIDAQLVDANGRVLDRRSMGEDLFWAIRGGGGGSFGIVISWKIKLVHVPSTVTIFSVGRTLEQNATQLLHRWQYVAPNLPNDVYPVVTISTTNASENGTKTVLATFVSLFQGGANEFIPLMQERFPELGLVKEDFIEMTWVESLLFMNGLSNETSEILLDRSNRYTILPPFVKSKSDYVREPMPEIALQGLLLHLLEVDEGGTAVQNFYAYGGIMDEISETETPFPHRNGTLYKISYVIAWQEEENNNSQRYISWMSKLYSYMGPFVSKSPREAYVNYRDLDIGSNNYYGKTSYKQASNWGRKYFKNNFDRLVYVKTKTDPKNFFKHEQSIPPRLHYST

>GhCKX45D

MKDSNSATFQLVTIVLFLSLSWRATSDSSSQVDKFLQCLANNSALMLESIYTPSNSSFENALQAYIRNRRFLTPETPKPVAIVAPNHVSHVQATVICAKDNGVQIRVRSVGHDYEGLSYRSNVTFVILDMFNLRSIDVDVDNEVAFVQAGATIGKLYYKIANESKGHAYPAGVCPSLGTGGHFSGGGYGNMMRKYGLSVDNILDAQVVGADGNILDRASMGEDLFWAIRGGGGASFAVIVSWKIKLVQVPEKVTVFTVPFTLEQGATDVAFKWQQVAPKLPEDLYIRQIASVINGSCPLEKKILVSFMGLFLGEADKLVPLVNQSFPELNLTKEDCKEMSWLESTVYWAGFPVGTPVQVLLNRTQGAPNIFKVKSDVKTVIPKEGLETIWQMLIGMGNTSAANISMQWNPYGGKMSEIPESSTAFPHRSVNLFLIQYVVLVRRGAQHPREAFLNYRDIDIGSSPSDQTNFTHAQVYGSKYFKDNFQRLTMVKAEMDPSNFFKNEQSIPPNFNFK

>GhCKX46D

MAFWRISICSLLLLLLSSSTMATASIYRPRKRPTSSLIQEKFIQCFTGSSQFYIPLSTAFFTPNNASFTSVLQSTAQNLRYLVPSMPKPEFIITPQHESQVQASVICAKRLDIHLRFRSGGHDYEGLSYVSQIESPFVIVDLSKLRSIKVDIQDNSAWVEAGATIGEVYYRIAEKSNIHGFPAGLCTSLGIGGHITGGAYGSMMRKFGLGADNVIDARIVDVNGRVLDRAAMGEDLFWAIRGGGGASFGIILEWKIKLVPVPATVTVFTVTKSLEQGATKLLYKWQTVADKLDEDLFIRVIIQKANAGKNNAKTVTTSYNALFLGNAERLLRVMQQSFPELGLTGKDCTETSWIKSVLYIAGYSSNTPAEILLQGRSTFKNYFKAKSDFVKEAIPETALEGLWKRLLEEDSPLMIWNPYGGMMARISGSQIPFPHRQGTKFKIQYLTLWQDEDNNASKHFDWIRRLYNYMAPYVSMFPRGAYVNYRDLDLGMNKNINTSFIEASLWGVRYFKDNFMRLVKVKSRVDPNNFFRHEQSIPPLPVQARY

>GhCKX47D

MHFLRNLIILFLICIAIKINLCVPTIPSSLKTLPIDGHFDFKQVHHAAKDFGNRYSFLPSAVLHPKSASDIATTVKHIWEMGPGSHLTVAARGHGHSLQGQAQAHRGVVINMESLQGPKMKVHTGNFPYVDVSGSELWINILHETLKHGLAPKSWTDYLHLTVGGTLSNAGISGQAFRHGPQISNVRQLEVVTGKGEVVNCSEKQNSDLFYSVLGGLGQFGIITRARISLEPAPKMVKWIRVLYTDFATFAKDQEMLISGESTFDYIEGFVIINRTGLLNNWRSSFNSNDSAQASHFKSDGRTLFCLELAKYFNPEEMAIVNQEIMTSLSQLNHIPSTLFQSQVPYIEFLDRVHISEIKLRSKGLWEVPHPWLNLLIPRSNIHIFAQQVFGNILTNTSNGPILIYPVNKSKWDNRTSVVLPEEDVFYLVAFLSSAAPSSTGSDGLEHILSQNKRILELCEIDGLGVKQYLPHYSTNGEWRSHFGPQWEAFVHRKSTYDPLAILAPGQRIFQKAAPLSL

>GhCKX48D

MKFLQHSVLPFLIVTLSLNGATLAHSYGDFLHCLSLRISNSSTISKVIYTQNNPSYSSVLNASIHNARFSTPTTPKPYAIITPRKTSHVQSTIYCSKNHGFQLRIRSGGHDVEGVSYVSQVPFVILDLVNFREVKVDTENEVAWVQSGATTGELYYGIASKTQTLGFPAGICHTIGIGGHLSGGGFGVLGRKYGLAVDHVIDAKLVDANGRVLRRKSMGEDLFWAIRGGGGNTFGVVLAWKIKLVPVPPVVTVFTVNKNLEQNATKTFHRWQYIAHKLPRDLFTTVWVMKVNSSQVGRKTVQASFKGMFLGRIDVLIPLIQYAFPELGLARENCTEMSWVQSVLYFGALPIEPVEILLNRSALPRLSLKAKTDYIRQPMSETGIEGFMNMFLEEGTDFAITMIEAFGGKMDEIRENELPFPHRSGILFESVYIVQWTNEEDAGLCINWMRRLYSYMSSYASKSLRGAYYNYKDLDLGTNNINGYTSYEQASVWGLKYFRNNFKRLVRIKTMIDPMNFFSNEQSIPPLLSP

>GhCKX49D

MIAYLGRIVHDNDAESKLDDDVSSISKSLDLQGSIEKGDVSSLASKDFGGLYSVKPLFLIKPSGAEDISRVVKLASRTSNLTVAARGNGHSINGQAMAEGGLVIDMSSTEKNHFEFLPINGSHYIDVSGGALWEDVLTRCVSRYGYAPRSWTDYLSLTVGGTLSNAGVSGQAFRYGPQTSNVTELEVVTGKGEITVCSETLNSELFFGVLGGLGQFGIITRARIKLQQAPDMVRWIRVVYSEFEEFTRDAEFLVTQKEGESFDYVEGFVFCNNDDPFNGWPSVPLDPGHEFNPTHISQTAGSVLYCLEVAFHYRNSDHPTVDTAVNGLLGRLRFVEGLKSQVDVSYTKFLLRVNRAEEQVKANGTWDGPHPWLNLFVSKSDVVNFDRTVFKTMLKDGVGGPMLIYPLLRSKWDDRTSVVLPEGEIFYIVALLRFVPNGPSVEKSVAQNREIVNWCIKVGLDFKLYLPHYQSKEDWERHFGNRWSRFVERKASFDLMAILAPGQNIFRRDPPNIIISREF

>GhCKX50D

MRGLPILVSIILFSISLVTSKSYPNSVVDNFLQCLPKHYRNPSKPIAKAIYTPTHPSFQSVYELHTYNLRVLTQANATHKPMAIVAALHESHVQAAVICAKESGLQVRIRSGGHDYEGLSFSSSVPFVIIDLSNLRSIKIDMKTETAWVQAGATTGELYYRIAEKSNVHAFPAGVCTTLGIGGHFTGGGYGNMLRKFGLSIDNVVDAQLINAKGRILNRKSMGEDVFWAIRGGGGTSFGIILSWKIKLVRVPPKVTVFQVAKTLEQGATVLVHKWLQVSHKLDKDIFIRIMPVTVAGTGNGNSTVRVSFIGHYLGRTNRLLPSVNASFPELGLQRKDCTEMSWIESTLYWAGFPNGTSTDVLLNRVPNKVFFKTKSDYLKNVMPKAGLETLWKVMMEIGNMWMQMSPYGGRMAEISESETAFAHRAGTLYLVQYTAHWSEGSSEATKKYVELMRKLYAEMAPYVSTKPREAFLNYRDLDIGSNNTDFEAAKVYGAKYFKGNFQRLAEPSNPVWGAIYRPSNASFPTILQAYAKNLRFSTPKPLAIVTTMQESHVQATVICAKSQGLQIRIGSGGHDYEGLSYVSEIPFVVLDMFNLRSKTVSYGFPAGAYPTLGVGEHFSGGGYGNLMRKYGLAVDNIFDARLVDVNGTILNRESMGEDLFWDIRGGGGGSFGVILSWKIKLGLQCTKNLKPGLNRYCLSMATSCPEVARRSIHKAQRLQRMSWMESTVFYPGFPRGTSIEVLLRRPQNGEDFYVVESLWRKDGWDLGI

>GhCKX51D

MATKLLLTFAICRLIVTVGLTLDPTELLLLGVDAQLSVDPTDVKAASLDFGLLTGAQPPLAVMHPASSQDVAQLVKAAYGSNFGFTVSARGHGHSINGQAQTANGVVVQMSGSIGGSGVASGRKPPYPRVWPQERFVDVWGGELWIDVLRSTLQHGLAPKSWTDYLYLSVGGTLSNAGISGQAFNHGPQISNVHELDVVTGKGELLTCSEEQNSEMFHAVLGGLGQFGIITRARISLEPAPQRVRWIRVLYSNFSTFTRDQEYLISLHEQPASQKFDYVEGFVIVDEGLINNWRSSFFSPHNPVKISSLEPNGGVLYCLEIAKNYHESTASTIDQEVESLLKKLNFIPASVFTTDMPYVDFLDRVHKAELKLRSKGLWEVPHPWLNLFVPKSKIADFDKGVFKGILGNKTSGPILIYPMNKNKWDHRSSAVTPDEDVFYLVALLRSALDNGEETHSLEYLTNQNRQILRFCDEAGITVKQYLPHYTTHQEWVDHFRNKWDRFYRLKMEFDPRHILASGQQIFTPTNMASWQ

>GhCKX52D

MATKLLLTFAICRLIVTVGLTLEPTELLRLGLTVDPFDVESASVDFGLMTKVEPLAVLRPSSAKDVAQLVKAAYESSHGFTVSARGHGHSINGQAQTGTGVVVQMSGVGSGGKPRVWGGDMYVDVWGGELWIDVLKSCLAYGLAPKSWTDYLYLSVGGTLSNAGISGQAFHHGPQISNVHELDVVTGKGELMTCSKEQNPELFHAVLGGLGQFGIITRARISLEPAPQRVRWIRVLYSNFSAFTKDQEYLISLHGNQKFDYVEGFVIVDEGLINNWRSSFFSPRNPVKISSLGSNNNGGVLYCLEITKNYHESTAETIDQEIEALLKKLNFIPTSVFTTDLPYVDFLDRVHKAELKLRSKGMWEVPHPWLNLFVPKSKIADFDRGVFKGILGNKTSGPILIYPMNKNKWDGKSSVVTPDEDVFYLVAFLRSALDNGEETQSLEYLTNQNRQILKFCAEDGMKVKQYLPHYKTQSEWKEHYGSKWDRFQRMKMKFDPRHILASGQNIFTPTFLSSSNMMVMDIPIPIKQKARESIDF

>GhCKX53D

MAVALPSFLTAIMIMSRLMAFIGITRNNDMSSKLQSLVIAPKLSYDPSAIESASQDFGHIVKAVPQAVLLPSSPWDIASLVNFSYSNSVPFIIAARGNSHSVNGQAMAKNGVVIDMTSMKNGNGTGIRIASDGSYADVGGQQFWIDVLNATLGLGLTPVSWTDSLYLTVGGTLSNAGISGQTFQYGPQISNVYEIDVITGTADFVTCSPNNNSDLFYAALGGLGQFGIITRARIPLEPAPKRVKWVRMLYTDFSDFTRDQELLISKNGRNDNKALNYLEGSLLLDQGSLDNWRSSFFQPQDQPKIISLITKFRIVYCLEIVKHYDGQTKTTVDKDLQQLLKGLSYLPGFMFEKDAKYEEFLNRVHSEELKLKAKGLWDVPHPWLNLFIPKSKISDFNDGVFKSIVLQRNITTGPVLVYPMNRKKWDDRMSAVIPDEEIFYTVGLLQSSGFDDWRTFEDQNKEILQFCEKAGIKVKQYLPHYTTKEGWVNHFGSKWSTFQKRKLQFDPKLLLSPGQRIFNNNE

>GhCKX54D

MIACLGRIVHDSDAESKLDDDVSTIFNSLNLQGSIENGDVSGIASKDFGGLYSVKPLYLIRPSGAEDISRVVKAAAGTPHLTVAARGNGHSINGQAMADGGYVIDMRSTGENHFNLLTVNGSPCIDVSGGALWEDVLRRCVSRFGLAPRSWTDYLSLTVGGTLSNAGVSGQAFRYGPQTSNVTELEVVTGKGDITVCSETQNPELFFGALGGLGQFGIITRARVKLQPAPDMVRWIRVVYAEFEEFTRDAEFLVSQKEDESFDYVEGFVFCNNDDPVNGWPSVQLNPDQEFNPAHLPQTAGPVLYCLELAVHYRNSDQPSTVDMAVSRLVGGLGFVDGLISQVDVSYMGFLLRVKRAEQDAKANGVWDNPHPWLNLFVSKSDIVEFDRTVFKKMVKNGIGGPMLIYPLLRSKWDSRTSVALPEGEIFYIVALLRFAPKGPSVEKKVAENREIVKWCIKEGLDFKLYLPHYRAKEDWKRHFGNQWTRLEKRKANFDPMSILAPGQRIFKRTNQ

>GhCKX55D

MAFSSTMILPLLLVLLSAFSATSKSVQENFMQCLDANSEHPIPISAFCSQTNSSFTSVLNSTAQNLRYLMPLVPKPEFIFIPVYESHAKSAVICAKRLAIHLRFRSGGHDYEGLSYASEIETPFILIDLIQLRSINVDIDDNSAWVQAGATVGEVYYRISEKSKTHGFPAGLCSSLGIGGHITGGAYGSMMRKYGLGADNVLDARIVDVNGEILDRAAMGEDLFWAIRGGGGASFGVILAWKIKLVAVPETVTVFTVPKTLEQGATKILYRWQQVADKLDDDLFIRVVIQVTKTSQTGKRTVTTAYNALYLGDAERLLQVMDQSFPELGLARKDCIETSWIKSVLYIAGFPSETPPEVLLEGKSLFKNYFKAKSDFVQQPILETALEKLWEILLEEESPLMIWNPYGGMMANISDSAIPFPHRKGNLFKIQYVTSWYEGSKDATRKHMDWIKGLYDYMSAYVPTSPRGAYVNYRDLDLGMNHNNASYTEASVWGAMYFKGNFRRLVKIKSKVDPGNFFRHEQSIPVVLE

>GhCKX56D

MAISWPLVVSLLLSISSLATSASNSDSVHEAFVQCLLDNSHPSHPISEAIFTPQSPSYATVLQSYIRNLRFNETYTPKPFLILTALHQSHIQAAIICAKKGNIQMKIRSGGHDYDGLSYVATVPFFVLDMFNLRSIDIDMETETVWVQSGAILGELYYRISELSKTHGFPAGVCPTVGVGGHFTGGGYGNMMRKYGLTVDNIVDAYFIDVNGRIHDRKSMGEDLFWAIRGGGAASFGVVLAYKIKLVRVPEIITVFRVEKTLEDNATAIVDQWQHVASKLPKELFVRLVIDVVNSSTRTGGSTVRVSFISLFLGDSKTLVSIMNENLPLLGLSQSDCIETSWIRSVLFWTNITIDSPTDVLLNRTPSLSYLKRKSDYVKQPIPKTVLEGIWEKMIELQPAQMIFNPYGGRMAEIASTATPFPHRAGNLWKIQYLANWNQGGAETAQRYIELTRKLHRYMTPFVSKNPREAFLNYRDIDLGVNHNDRGSYLEGRVYGIKYFKGNFNRLVHIKTKFDPTNFFRNEQSIPTLPY

>GhCKX57D

MNSSSSKLFILSTSFLLSISSITSNSVLDNFLQCLPIHSNTSNPITNSIYVPNNSSFQYLYELRANNLRIISSSTISKPLAILTARHASHVQAAVICAKIHSIQLRIRSGGHDYEGLSYVSDIPFVILDLFNLRSIKINITSETAWVQAGATTGELYYKIAEKSKVHGFPSGVCTTLGIGGHFTGGGYGNMIRKYGLSIDNVIDACLIDVNGTIHNRKSMGEDVFWAIRGGGGTSFGVILSWKIKLVHVPRKVTVFKVQRTLDQGATDIAYSWQHIAPKLPKYLFIRLQPEPITIGNGNKTIRVSFIGQFLGRSRKLMNLMNEEFPELRLQQNDCIEMSWVESTLFWAGFTNGTSIDVLLNRVVENKVFFKTKSDYYKNVIPKQGLVMLWEMLMDIGNIFVQLNPHGGRMDEISETETAVHQRGGYLFKVQYTVYWSESDGGIGAAKRYVEMSRRLYGAMAQYASSDPREAFLNYRDLDIGCNESNDTDFGVAEVYGTKYFNNNFMRLARVKAMVDPENFFKNEQSIPPLPSSH

>GhCKX58D

MPNPMRPYLILSVVFFFNLYHSMAVPDPTHQALLQCLTQSIPTDTASSIIVSKSNPSYTSVLRAYIRNARFNTSSTPKPLIIITPLDESHVSAAVICSQKLGFQLKIRSGGHDYEGLSYVFDNPFFVLDMFNLRSITVNMADETAWVGAGATLGELYYNIWKNSKVHGFPAGVCPTVGVGGHLSGAGYGTLIRKYGLSVDHVVDAKLVDVNGKILDRKTMGEDLFWAIRGGGAASFGVVLSYKIKLVPVPETVTVFRIERLLTENATDITFKWQTIAPTTDENLFMRMLLQPVTRNKKKTARISVIALYLGDSDSLVSLLQKDFPELSIGKSNCNETTWIDSVLWWANFNLGTPPTALLDRDLNDAGFLKRKSDYVQTPIPKSGLESLWQKMIELGKVGMVFNAYGGRMDQIKPDETPFPHRAGNLYKIQYSVNWDQPGSEADKNFTTQAKLLHDFMTPFVSKNPRSAYFNYRDIDVGSTKKWSYEEGKVYGESYFNGNYERLVDVKTAVDANNFFRNEQSIPPRSSKI

>GhCKX59D

MAVSFPIPSYFTAIFIISRLMSIIGISKHWNNKLLPPLDNITDKLSLDPSAIESASQDFGHIVKSIPKAVLQPSSIADIASLINFSYNSSIPFTIAAKGHGHSVRGQAMASDGVVVNMTSMKKHRNGTGIWVSNDGVYADVGGEQLWIDVLNATLKHGVAPVSWTDYLYLTVGGTLSNGGISGQSFRYGPQISNVYEMDVITGKAEIVTCSPNKNSELFYAALGGLGQFGIITRARIPLEPAPKRVKWIRMLYNDFTAFTRDQELLISINGRHDSHALDYLEGSLLMDHGSPDNWRSSFFPPKHHPKITSSITNHRIIYCLEVVKHYDDQTQNTVDKELEQLLKGLSYMPGFMFEKDVLYAEFLNRVLRGELKARSEGLWDVPHPWLNLFIPKSQIEGFNDGVFKGIVLERNITTGPVLVYPMNRKKWDDRMSAVIPDEEIFYTVGFLHSSGFDDWEAFDDQNKEILKFCEDAGIGVKQYLPHFTSKDEWVHHFGSKWETFQQRKFQFDPKMILSPGQRIFNNN

>GhCKX60D

MYYFRWLLGFLHLLVWGSIILVHAIPAPDPVQCNRTICTLSNSYGAWGDRKDCSVKSVVYPTTEEELRSAVAHANKNKLKVKVVSKFSHTIPKLACPSSLGHDSLLISTAKYDSGIEIDSVNLAVTADAGVALRDVIDKVEEAGLSLVAAPYWEGVSVAGMISTGAHGSSWWGKGGAVHDHVIGLSMIVPGNESEGYAKVKQIGAQDQLLNAAKVSLGILGVISKVKLSLERGFKRSITYNFTSDSSIENNFMEHGKKYEFGDITWYPSKHTAVYRYDSRVPMDTPGDGINDFLGFQSNEILISKSVRASEKLFESTKSVNGECTLADTTLWYKKQIGNGLKNNGQIFTGYPVVGRQGKMQTSGSCLYSPKTRIDASCAWDPRIKGLFFYESTAIFTATKFVDFIKDVKKLRDLKPENFCGIDHYNGFLIRYIKASKAYLGQSEDSIVVDFNYYRADEASTPRLNQDVMEEVEQMAFFKYGARPHWAKNRNLAFLKVQSHPRAP

>GhCKX61D

MVNLSLLLLHFLISSLSVSGSAATDQRNIMSCLNYYNISNYTISSNVHNHDYSILLNFSIQNLRFAEPTIPKPIAIILPENKEQLINTVVCCTKGPWEIRVRCGGHSYEGTSSVASDGAPFVIIDMMNLKSVSVDLGNETAWVEGGATLGETYHAIAESSGIHGFSAGSCPTVGTGGHIGGGGFGFLSRKYGLAADNVIDALLLNAEGELLDRQAMGEDVFWAIRGGGGGIWGIVYAWKIKLLRVPKTVTSFIVSRPGTKAHAANLVNKWQHVAPNLEGDMYLSCAVGAGLPQAKSIGISATFNGFFLGRKREAVLILRRVFAELGVAEEDCKEMSWIESVLFFSGLGDGALVSDLKNRYLHDKHYFKAKSDYVRNPISLTGIRTAIDILEKQPRGYIIMDPYGGIMNNISNDSIAFPHRYGNLYTIQYLVEWHQEEKNRSNEYREWIRDFYDAMASHVSWGPRAAYVNYMDFDLGVMELINTSVLSEDTVEMARVWGEKYFLNNYDRLVKAKTLIDPNNVFKNQQGIPPSTTIGLKARTF

>GhCKX62D

MRIAYLDRTVHETDGEPKPNGGVSTLSKSIDLQGSVETGDKTTIASKDFGGLYSTKPLALIKPAGSDDVSRVIKAASRIPRMTVAARGNGHSINGQAMTNGGLVIDMRSTEENHFRLLNINGSFFIDVSGGALWENVLTRCVSRFGLAPRSWTDYLSLTVGGTLSNAGVSGQAFRYGPQISNVAELEIITGKGDIMVCSETRNPELFFGSLGGLGQFGIITRAMVKLQPAPDMVRWIRVVYTEFDEFTRDAEFLVSRDDGESFDYVEGFVFCNNDDPVNGWPSVPLDPVHGFNQGIIPQTGASVLYCLEVAFHYQKGDHPSTVDKAVAGLLKPLRFVEGLKSQVDLSYVEFLLRVKRAEEQAKANGIWDAPHPWLNLFISKSDIVDFDQTVFKKMVKDGIGGPMLIYPLMRSKWDNRTSVALPDSEIFYLVALLRFVSRGPSVEESVAQNREIVEWCIREGLDFKLYLPHYQSKEQWKRHFGNQWTRFVERKASFDPMAILAPGQNIFKRTHLS

>GhCKX63D

MGSPVCGFLKQNNIIFLRFFAILVLSCIPDGTNLCSNPSFDTLTIPPHSSSSSIPSSFKTLTLDGYFSFENLKHAAKDFGNICHYLPIAVLHPKSVSDISSTIKHILYMSSVTKLTVAARGRGHSLQGQAQAYQGVVINMESLDRPSMYIENGEVPYVDVSGSELWINILHETLKYGLSPKSWTDYLHLTVGGTLSNAGISGQAFRHGPQIDNVYQLEVVTGTGEVVTCSDKENADLFYGVLGGLGQFGIITRARISLGPAEKMVKWIRVLYSEFSTFSNDQEHLISSNNSFDYIEGFVMINRTGLLNNWRSSFNPKDPIQASQFSSDGKILYCLEMVKYFNPEKIDVLNQDIEKLLSELNYIPSTLFLSEVSYVEFLDRVHLSEIKLRSKGLWEVPHPWLNLLIPKSRILDFAEGVFGNIVKDNNNGPILIYPVNKAKWNNRTSMVTPEEDIIYLVAFLSSALPGTDGLEHIMTQNQHILDFCAKAQLGAKQYLPHYHTQDEWQAHFGTQWETFVQRKSAYDPLAILAPGQRIFQKAISIT

>GhCKX64D

MGFSFWLARLRSCSNKSAFSNAFRDSFYHYKSQLNTCQKNLPSTIAEKTNSHAFSWSSCLLPLAFAVSAGSLTFQSHNNHPSLCEPSNLDSRKVTIGGKASTEFVVKGTHKEVPQELIDELKAICQDNMTLDYDERFYHGKPQNSFHKAVNIPDVVVFPRSQEEVSQIVKSCNKHKVPIVPYGGATSIEGHTLSPNGGVCIDMTLMKRVKALHIRDMDVVVEPGIGWMELNEYLEPYGLFFPLDPGPGATIGGMCATRCSGSLAVRYGTMRDNVISLKVVLANGDIVKTASRARKSAAGYDLTRLMIGSEGTLGVVTEVTLRLQKIPEHSVVAMCNFPTIKDAADVAIDTMMSGIQVSRVELLDEVQVRAINIANGKNLPEVPTLMFEFIGTEAYSHEQTQIVQRIVSEHNGSDFVFAEDPEAKKELWKIRKEALWACFAMEPNFEAMISDVCVPLSNLAELISRSKQELDASSLVCTVIAHAGDGNFHTVILFDPNEEEHRREAERLNQFMVYTALSMEGTCTGEHGVGTGKMKYLEKELGIEALQMMKQIKTALDPNNIMNPGKLIPPHVCF

>GhCKX65D

MGELYYEIAQKSGTLAFPGALCHGVAFGRYISGEGYGLLFRKYGLAADNVIDAEFIDANGRILKTENQWGKICFGIFVAAVVEALGLSFHGRLN

>GhCKX66D

MGFQSLVIIFLLCISMANSDHLKQNKTILQCLTDHSIASPSISSVTFFPTDPSFTSTLQSYTRNLRFTSTTTPKPLFIVVPSHVSHIQASIICCKTHGLEMRIRSGGHDYDGLSYVSKAPFMILDLFNLRSVIVDNGTAWVESGATLGELFYAISQKSKIHGFPAGVCPTVGVGGHFSGGGYGNMMRKFGLSVDNVIDAKLVDVNGNVLDRESMGEDLFWAIKGGGGASFGIIISWKIKLVSVPEIVTVFKIEKTLEQGVTGIVHKWQYIADKIDPNLFIRVVLLPVNKKHLQSIKAKFIGLFLGNGQELSSLMNEAFPELGLSFDQCIEMSWIESILFWSNYPKGTSLDALLDRQPQQEKYLKKKSDYVQEPISKENLEGIWNKMIELKRPALTLNPYGGKMSEISEFETPFPHRAGNIYKIQYSVTWKDDGVEASGRSLDQIRKLYDYMTPYVSKSPRSSYLNYRDVDIGINENGNASYSEGVIWGRKYFKGNFERLVQVKSKVDPGNFFRYEQSIPCLGSWKSITAE

>GhCKX67D

MLNPKCFNFLTLFISLLSLPSPTISQSSSLTNFLHCLHYGSDPIVSQSIYIASNPAFQTILQARIKNRRFLNPETLKPVAIVVPTRIDHVQGTVICAKDNGLQIRIRSGGHDYEGLSYRSNVTFIILDMSNFRSIDIDVKTETAWVQSGATLGELYYHIANKTNMHGFPSGICPTVGIGGHFSGGGYGNLMRKYGLSVDNILDIVAVDALGNVHDRASMGEDLFWAIRGGGAASFAVVVSYKIKLVRVPNKVTVFRKGFTLEQGATDLVHKWQQVAPNINEEFFIKVKLEPSFINGNQTVTATFIGFFLGRREKLLPIISKTFPELNLTQQDCHEMRWVETTLFWAGFPIGTPIETLLNRTIWTPLFFKNKSDYVKNVIPKESLNKIWKMTMAMMNRNDINKTRFDLECSPYGGKMNVIPESNTPFPHRKGNLFLIQYAFSWIDEGNNVSFNNIKKLRKLYDGMTPYVSKDPRECFLNYRDLDIGSNRSNETSFDDAKIYGRKYFKDNYTRLTKVKASVDPNNFFKYEQSIPPIK

>GhCKX68D

MPPLNHYSNAPISSKNPKKKEMKISSSIFPIISIFILLSISSVTPSDSDDSDDFDDFFQCLPKQSDSSIPITDAILTPNNSSFQYIYQLRANNLRTFLSATSRPVAIITARHPSHAQAAVICAKRHDFQLRIRSGGHDYEGLSYTSDVPFVILDMFNLNSIDIDMSTETAWVQAGATTGELYYRIAEKSNVHGFPSGVCTTLGIGGHFSGGGYGFLIRKYGLSIDNVIDAQLIDANGRILNRKSMGEDVFWAIRGGGTTSFGIILSWRIKLVRVPPRVTVFTVQRTLEQGATELAYRWQQVAPKLPKDLFIRLQPEPINNGGNNKTVRVSFIGHFLGQADVLFRLMNVSFPELGLTRNDCLQMSWVESTLYWAGFSNGSSIDVLLDRVAVNKVFAKEKSDYYKAVIPKQGLETLWQVLMDIENIFVQMNPYGGRMEEISDSETAFAHRAGNLFKVLYGIQWSESEGGVNATARYVELSRRLYNAMAPYASSNPREAFINYRDLDIGSNESDETDFEDAKEYGAKYFRNNFIRLADVKAKIDPKNFFKNEQSIPPLPSH

>GhCKX69D

MKASSYTCFIVSIFVLFSISSAASYNPVDFDAFLQCLPQHSDHTVSIAGAILTPNNASFQSTYQLRANNLRILLSATSRPVAIITALHPSHAQAAVICAKRHGFQLRIRSGGHDYEGLSYISDVPFVILDMFNLKSIDIDMKTETAWVQAGATTGELYYSIAQKSDVHGFPSGVCTTLGIGGHFSGGGYGFLMRKYGLSIDNVIDAQLIDANGRILDRKSMGEDVFWAIRGGGTTSFGIILSWRIKLVRVPPRVTIFTVQRTLEQGATELAYRWQQVAPKLPKDLFIRLQLVPINNGGNNKTVTVSFIGHFLGQADGLLRLMNVRFPELGLTRNDCSEMSWVESALNWAGFPNGTSIDVLLNRVQVDRVFYKTKSDYYKAVIPKQGLETLWQVLMDIEDIFVQFNPYGGRMEEISESETAFAHRRGNLFKAQYGIQWSESDGGINATGRYVEMSRRLYNVMAPYASSNPREAFFNYRDLDVGSNESGTDFEVAKEYGAKYFRNNLMRLASVKAKIDPENFFKNEQSIPPLPTPPSH

>GhCKX70D

MTPPLSPPSLLPLLLVAFNICFSLAASNSVYESFVQCLKTRSNSSDNISDIVYSHSNATYETVLEQYIRNARFNTSSTPKPVIIITPLTESHVSAAVICSNNIGFQLRIRSGGHDFEGVSYVSDQPFFILDMFNLRSISINMADQSVWVQSGATLGELYYRIWEESKVYGFPAGVCPTVGVGGHISGAGYGNMVRKYGLSVDYVVDAKIVDVNGNILDRKAMGEDLFWAIRGGGGASFGVILAFNIKLVDVPETVTVFKLERTLEQNATDVVYKWQSVAPTTDDNLFMRMLVQPVTLNKQKTIKISIMALYLGDVNSVVPLLAEDFPELGLVTEDCFEMSWIESALWWASFGKGTSPTVLLDRESYHVKFMKRKSDYVKTPISKDGLQWLWKKMIELEEPGLVFNPYGGKMNEIKETETPFPHRAGNLFKIQYSINWKDMGIEADKRSRSLVNRLHSYMTSFVSKNPRSAYLNYRDLDIGITKNWSYQEGKVYGESYFNGNFERLVDVKTVVDPHNFFRNEQSIPPRTIKAWNEKNEGSIPPSTSKAWNKSKPYVMIILFMAIGHII

>GhCKX71D

MEISKPLLVFFSLVFFNLSFSWAAPDPTYQSLLQCLSEIIPSPNVSAVIVSNNNPSFASILESRIHNARFNRTSTLKPTIIITPSDESHVSAAVICSQKVGFQLKIRSGGHDYEALSYTSDKPFFLLDMYNLRDVSVDIPDESAWVQTGATLGELYYHIWEKSNVHGFPAGVCPTVGVGGHIGGAGYGTMIRKYGLTTDYVIDAKIVDVNGKILDRKAMGEDLFWAIRGAGGTNFGVVTAYKIKLVKVPEKVTVFRVERFLDNNGTEVAFKWQTVGATTDPNLFTRMLLQPNMKDKQRTVKVTVMGLYLGDINGLLTLLNKDFPELRLNKENCTEMPWIDSVLCWANFDLGTPPNVLLDRNNTDTKFVKRKSDYVQTPIPRDGLESLWQKMVQNEKDDPSIEADIKYTTQAKAVHEFMTQFVSKNPRRAYLNYRDIDIGSAKTWSYEEGKVYGESYFTENFDRLVDVKTAVDPNNFFRNEQSIPPRSTKTA

>GhCKX72D

MSISMAISLLFSLLFLNISISSAASNPTYQSLLQCLSQSINPSQNVSTILFSNTNPSYASVLQAYIRNARFNTSSTPKPVIIITPLEESHVSAAVICSQKVGFQLKIRSGGHDYEGLSYVSDKPFFVLDMFNLRSISIDMTDESAWVETGATLGELYYNIWEKSNVHGFPAGLCPTVGVGGHLSGAGYGTLMRKYGLSSDYIVDAKIVNVDGKILDRKAMGEDLFWAIRGGGAASFGVVLAYKVKLVRVPETVTVFRLERLLADNATDIALKWQSIAPTTDENLFTRMLLQPVTRNRQRTMRVTVNGLYLGNADGVAALLSKDFPELGLKNENCTEMRWIDSVLWWANFDAGTPPTALLDRNVNDADFLKRKSDYVQTPISKNGLESLWQKMVELGNVGLACNAYGGRMDEIDDKETPFPHRKGNLYKIQYSVNWNEPGNETEMNRTSQAKALHEFMTQFVSKNPRRAYLNYRDIDIGVAENWSYEEGKVYGESYFAGNYERLVDVKTAVDPNNFFRNEQSIPPRTK

>GhCKX73D

MAFPSISSLFSLLLVLHLSSSTTASTCHAKSFKLNPIQEKFIQCFKANSEIPIPASTEFFTPNNASFFTVLQSTAQNLRYLEPSVPKPEFIIMPLNESHVQAAVICSKELGIHMRVRSGGHDYEGMSYVSAIESPFILVDLSKLRSVKVDIEDNSAWIEAGATIGEVYYRIYEKSKIHGFPAGLCTSLGVGGHITGGAYGSMMRKYGLGVDNVIDARIVDVNGRVLDRAAMGEDLFWAIRGGAGGSFGIILQWKIQLVPVPSTVTVFTITKSLQQNSTKIFHRWIEVADNLDNDLFIRVIIQTAMINGEKTVTTSYNSLFLGEADRLVEIMQQSFPELGLTRKDCIETSWIKSVLYIAGYPSNTPPDVLLQGKSTFKNYFKAKSDFVKSNIPETALEGLWKRFMEEDIPLMIWNPYGGMMARISESETPFPHRKGNKIMIQYVSAWQDGDKNESKHIDWIRRLYNYMAPYVSMFPRTAYVNYRDLDLGTNKNASTSFIEASGWGVKYFKDNFNKLVKVKTKVDPENFFRHEQSIPPLPVEARF

>GhCKX74D

MEKWRATTNLRRSLKSILNRQLSSVSEFRYLNEKRSCQSSFNLIRDCKSLGQVNAIQHRCFSSASTLVQRNPSFSTLNSDDISYFKGLLGEKSVIQDEDRLETVNTDWMHKYKGSSKLLLLPRSTEEVAQILRYCNSRCLAVVPQGGNTGLVGGSVPVFDEVIVNVSSMTNIISFDKVSGILVCEAGCILENLISFLDNQGFIMPLDLGAKGSCQIGGNVSTNAGGLRLVRYGSLHGNVLGLEAVLANGDVLDMLGTLRKDNTGYDLKHLFIGSEGSLGIVTKVSILTPPKLSSVNIAFLACNDYSSCQKLLMEAKRKLGEILSAFEFLDTEAMNLVLHQLDGVRNPLPASMHNFYILIETTGSDESYNREKLEAFLLSSMEGGLISDGVLAQDINQASSFWRIREGVPEALMKAGAVYKYDLSLPVEKMYDLVDDMRIRLGDLATVVGYGHLGDGNLHLNVSAPEYDDKILEQIEPYVYEWTSKHRGSISAEHGLGLMKANKIYYSKSTETVQTMASIKKLLDPNGILNPYKVLPHSLNS

>GhCKX75D

MQKHKMKSNLPIPLLFLSLLFSFSWAALGLTHQQQHHSFLQCLNRHFGNANSISTVIYTQTNSSFSSVLEFSLRNARFSTPNTLKPLVIVTPSHVSHIQATINCSRTHGLQIRIRSGGHDYEGLSYVSQVPFVIIDLINLRSIDVDAENKTAWIQAGATIGELYYRIAEKSSTLAFPAGVCPTVGVGGHFSGGGYGMLMRKYGLAADQIIDAQLVDVNGKLLDKNSMGEDLFWAIRGGGAASFGVVVAWKVKLVPVPSTLTVFTVNRTLEENGTMLVHKWQSIAPKIHEDLYIRLFLRAVNSSQQEGKRTIQASFVSLYLGRADELIDLMQESFPELGLVKEDCIEMSWIQSIMYFPSDIPEDAPLEILLNRTGSAGIFKGKSDYVTQTIPETALEGLWQRFYEDETESLEILFSPYGGNMDDIPETETPYSHRAGNLFNIHYVVGWSEEDASESQRYINFMRRLYRYMEPYVSKSPRRAYMNYRDLDLGTNNIGPYTSYKQASKWGLPYFDKNFNRLIHVKTLVDPTNFFRYQQSIPSLSRG

>GhCKX76D

MGKLKAVVVVTMISTVLLSILWRATLHLEDNESFVRCLLDHSHPSHPISSAIYTPKSSSFSSVLEFYIRNLRFNESSTPKPFLILTALHESHIQAAVTCGKSHGVQLKIRSGGHDYEGLSYVSTLPFFLLDMFNLRSIDVDIETETAWVQTGATLGEVFYRIAEKSKTHGFPAGVCPTVGVGGHISGAGYGNMMRKYGVSADNVLDALIIDANGRLLDRQSMGEDLFWAIRGGGGASFAVVLAYKIKLVRVPETVTVFQVDRTLEEDATDIVDQWQHVAYNLPQELFIRLMLDVVVKRSGEKTLRASFVSLFLGDSESLLSIMKERFPKLGLSKSDCIETSWVKSVLFWSNIPLETDIQVLLDRTPQTLDYLKRKSDYVREPIPKAGLESLWKKMMELEKPRMYFNPYGGKMAEIAAEEIPFPHRAGNLWKIQYLANWNEAGIEAANRYIDLTRRLHEFMTPFVSKNPRQAFLNYRDADLGSSSHGKASYSEARLNGMKWFMGNFDRLVQIKTEVDPTNFFSYEQSIPLLPHQVHLDDDM

>GhCKX77D

MSFFFPEMKASQYFSMSLFLLILLISCPWQISANPHLNNFLGCLDSFYSNHISQVIYTQNNASYSSVLNATIQNLRFSTPTTPKPLVIVTPLQTSHIQATIRCSRTNGLNLRIRSGGHDFEGLSYVSQVPFVVLDLTNFRSVKIDVKNKVAWVQSGAILGEFYSEIAKRSRTLAFPAGICHTVGVGGYLSGGGYGLLLRKYGLAVDNVIDAVFIDVNGRILKRKSMGEDLFWAIRGGGGGSFGVVLSWKVKLVSVPSTVTVFTIRKTLEENATNLVHQWQSVGHKLPGDIFSAVTMRKVNRNGKTTILVAFSSFFLGETNALITLMKAGFPELGLKKEHCTEMSWIESILYFGQIQNKSIDVLLDRSYKSPLNAPWFKTKLDYVKNPIPKAGFEKIWSKLYEEDAETAAMAFIAYGGKMAEIPESATPFPHRDGNLYHIAYTVGWDGEENTKSQRYMNWIRKFYSFMTPFVSKSPRGAYVNYRDDDIGTNNKKGETSYAKASVWGRKYFKNNFDKLIYIKTKVDPHNFFKHEQSIPVSV

>GhCKX78D

MLVSFPGQNNMLVLRSFMILFFSCITIKINLCFPSIPSSLKTLSIDGHFDFEQVEHAAKDFGNRYSYLPLAVLYPKSVSDVATTVNHVWQIGGGSELKVAARGHGHSLQGQAQAHRGVVINMESLQGLKMQVHTGNFPYVDVSGGELWINILRQSLKHGLAPKSWTDYLHLTVGGTLSNAGISGQAFRHGPQISNVYQLEVVTGKGEVVNCSEKQNSDLFYSVLGGLGQFGIITRARISLEPAPEMVKWIRMVYTDFATFTRDQEKLISGQSTFDYVEGFVIINRTGLLNNWRSSFNPQDPLQASQFKSDGRTLFCLELAKYFNHEDMVLVNQVSHSLIINGSSNGNNGTDKKGIDVLQEIKTSLSQLNHIPSTLFISEVPYMEFLDRVHISEIKLRSRGLWEVPHPWLNLFIPKSKIHSFAQEVFGNILTDTSSGPILIYPVNKSKWDNRTSVVIPEEDVFYLVAFLSSAVPSSTGNDGLEQILIRNKRILDYCEIAGLGVKQYLPHFSTQGEWKSHFGPHWEAFIRRKSTYDPSAILAPGQRIFQKPIAYS

>GhCKX79D

MFRSLSLKRSFKTHLKTLIHCPNHPQPPPPPSPVLFSSIRALSTASPPPSSSSDSELRKYLGYTALLAFCGVATYYSFPFSENAKHKKAQLFRYAPLPEDLHTVSNWSGTHEVQTRHFHQPENLKQLEELVKESNEKRVKLRPVGSGLSPNGIGLARGGMVNLALMDKVLEVDKEKKRVRVQAGIRVQQLVDEIKDYGLTLQNFASIREQQIGGILQVGAHGTGAKLPPIDEQIISMKLVTPAKGTIELSKEKDPELFYLARCGLGGLGVVAEVTIQCVERQELVEHTTVSNLKDLKKNHKKMLSENKHVKYLYIPYTDTVVVVTCNPVSKWRGPPKFKPKHTTDEAMQDIRELYKESLKKYRARDITTKSSDSNEPNINDFSFTELRDKLLSLDPLNKDHVMKVNHAEAEFWRKSEGYRVGWSDDILGFDCGGQQWVSETCFPAGTLSKPSMKDLEYIEELKKLIETNELPAPAPIEQRWTAHSQSPMSPASSSAEDDIFSWVGIIMYLPTMDARQRKEITEEFFHYRHLTQSQLWDKYSAYEHWAKIEVPKDKEELEALQARLKTRFPVDAYNKARRELDPNRILSNNILEKLFPLSDNV

>GhCKX80D

MPIIPVLYSNKLNALVLLKCLFLLVVLVGCSPPGEPVKCSTKDSNCTVTNSYGAFPDRTVCRAGNVVYPTSEQELVFIVSAATEAQRKMKVVTHFSHSIPKLVCPDGQDGLLISTKNLNRVVKINLAAMTMTVESGVTLRQLINEVAKAGLALCTVLVGTDHWRAFRYRSSWELVVGERELRS

>GhCKX81D

MPAIPMLFSNKFNVLLLWKCLCLLVVLVGCNPPGEPVKCSTKDSNCTVTNSYGMFPDRAICRAGNVAYPTSEQELVSIVSAATKSKRKMKVVTHFSHSIPKLVCPDGQDGLLVSTKNLNRVLKTDTDAMTMTVESGVTLRQLINEAAKAGLALPYAPYWWGLTIGGLLGTGAHGSSLWGKGSSVHDYVVEMRIVSPAKAEDGYAKVWELNERDKDLDAAKVSLGVLGVISQVTFKLQPLFKRSITYVRKDDTDLGDEAVTFGKLHEFADIFWYPSQRKAIYRIDDRVPINVSGNGVYNFTPFRSTLSLVLALVRSSEETQESSGDAEGKCLNAKLVTSTLQSSAYGLTNNGAIFTGYPVIGFHNRLQSSGTCLDSLEDSLITACPWDPRIKGEFFHQTTFSIKLYNGILMRYVKASTAYLGKQEDAVDFDITYYRSKDPMAPRLYQDVLEEIEQMALLKYNALPHWGKNRNLVFDGVMKRYKNGGEFLKVKNKYDPWGLFSSDWTDQVLGLRNGVIVLKEGCALEGLCICSQDVHCAPSKGYLCKPGKTFPDARVCARVNTKT

>GhCKX82D

MLFLRSFMILVLSCIAIKINLCFPNILSSLKTLPIDGHFNFEQLHHAAKDFGNRYSFLPLAVLHPNSVSDIATTVKHIWQMGPGSDLTVAARGHGHSLQGQAQAHGGIVINMKSLQGLKMQFHIGNLPYVDVSGGELWINILREGLKHGLAPKSWTDYLHLTVGGTLSNAGISGQAFRHGPQISNVHQLEVVTGKGEVVTCSGKQNSDLFHGVLGGLGQFGIITRARISLEPAPEMVKWIRVLYTDFATFIRDQEELISGESTFDYVEGFVIINRTGLLNNWRSSFNPQDPVQASKFKSDGRTLFCLELAKYFNRDETAVVNREIHSSLSQLNHIPSTLFVSEVPYIEFLDRVHISEIKLRSKGLWEVPHPWLNLLVPRSKIQTFAQQVFGNILTDTSNGPILIYPVNKSKWDNRTSVVTPDEDVFYLVAFLSSAVPSSTGTDGLDHILIQNKRILEFCEIARLGVKQYLPHYSTQGEWKAHFGSRWEVFVRRKSSYDPLAILAPGQRIFQKAVPYSQ

>GhCKX83D

MVGKITRAYEREVKEGDVKMASPKFGMSILFFFLCISFCSSTDQSFQQCFSSHLPPSNITYDVIFTQNSSQYSSILQSSIRNLRFSNASKPRYLVTPYNEDHIQATIICSKEHHMHVRVRSGGHDYEGLSYISDVPFIVIDLFHIRSVMVDIKNEYAWVGAGATLGELYYSISAKSNVHGFPAGSCPTVGVGGHISGGGFGTIFRKYGLAADNVIDAKMIDVNGNVLDRKSMGEDLFWAIRGGGGASFGVIFSWKLKLVRVPPTVTVFKTVKSLEQGATKLVQKWQNIAYKFHHDLFVHAVIQVTNPNSNQNPTVQVSFDCLFLGTTERLLSSIQRSFPELGVTQENCTEMSWIQSVLYFAGYSIAESADVLLNRTTQSTQSFKGKSDYVKEAIPKTGLEGLFKMVVEEETSVLILTPYGGRMKQIKSSATPFPYRSEYLYGIQYMISWDVAEETGKRIGWMRRLYKYMEPYVSTAPRAAYFNYRDLDLGRNSYPNTSYVESSEWGLKYFNHNFNRLVRVKTLADPHNFFWNEQSIPVLRLE

>GhCKX84D

MSDLQAPLRPKRKKGLVDFLVQFRWIFVIFFVLPFSTLYYFLIYLGDVRSEMKSYKQRQKEHDENVLKVVKRLKQRNPKKDGLVCTARKPWIAVGMRNVDYKRARHYEVDLSAFRNILEIDKQRMIARVEPLVNMGQITRVTVPMNLSLAVVAELDDLTVGGLINGYGIEGSSHIYGLFSDTVVAYEIVLADGRVVRATKDNEYSDLFYAIPWSQGTLGFLVAAEIKLIPVKEYMRLTYTPVVGNLQDLAQGYMDSFAPRDGDQDNPEKVPDFVEGMVYSPTEGVFMTGRYASKEEAKKKGNKINNVGWWFKPWFYQHAQTALKKGEFVEYIPTREYYHRHTRCLYWEGKLILPFGDQWWFRFLLGWLMPPKVSLLKATQGESIRNYYHEMHVIQDMLVPLYKVGDALEWVHHEMEIYPIWLCPHRLFKLPVKTMVYPEPGFEQHRRQGDTPYAQMFTDVGVYYAPGPVLRGEVFDGAEAVRKLEQWLIKNHSFQPQYAVSELNEKDFWRMFDADLYEHVRRKYGAVGTFMSVYYKSKKGRKTEKEVQEAEQAHLETAYAEAD

>GhCKX85D

MIACLGRIVQDTDADSIPDDDVSTLSESLDLQGTIESGGITGVAGKDFGGLYSVKPLALIKPSGTEDIARVVNAASRTSHLTVAARGNGHSINGQAMADGGFVIDMRSTEENHFKPLTIDGSHYIDVSGGALWEDVLKRCVSMFRLAPRSWTDYLSLTVGGTLSNAGVSGQAFRFGPQTSNVTELEVVTGKGEITVCSETQNSELFFGALGGLGQLGIITRARVKLQRAPDMVRWIRVVYTEFEEFTRDAEFLVTQEEGESFDYVEGFVFSNSDDPINGWPSVPLDPDHEFNPAYIPQTAGSVLYCLEVALHYRNSDRPSTVDTAVSRLLERLGSIQRLKFQLNVSYVEFLLRVKQGEEHAKANGNWDSPHPWLNIFISKSSIVDFDRTVFRKMLKDGIGGPMLIYPLLRSKWDSRTSVVLPEGEIFYIVALLRFVPKGPTVEKLVAQNHEIIKWCNKEGLDFKLYLPHYQSKEDWKRHFGNQWTRFVERKTSFDPMAILAPGQKIFKRTHIIKP

>GhCKX86D

MVMSFQFPAYFTAIFIITRVMSIMKISKPLDVHHKDIRAVDLATKLSVDPSAIESASRDFGGIVKAEPEAVLHPSAPQDIAALIKFSYSSSVPFGIAAKGHGHSARGQAMAENGVVVDMRSMANNRRNGTGIRVSIDRLYADVGGEQLWIDVLNATLEYGLAPVSWTDYLYLTVGGTLSNAGISGQTFRYGPQISNVLEMDVITGKADFLTCSPRMNSELFYAVLGGLGQFGIITRARIPLQPAPKGVKWVRLLYDDFSSFTKDQELLISKNGRKDKSALDYLEGSLLMDQGSPDNWRSSFFPHKDHPKIISLITKHGIIYCLEIVKHYDDRTKHTVDKEMKQVLQGLNYMPGFMFGKDVGYEEFLNRVRSGELKLKSQGLWDVPHPWLNLFIPKSQISDFNNGVFRDIVLERNITTGPVLVYPMNRQKWDDRMSAVIPDEEIFYTVGFLHSSGFDTWEAFEDQNKDIMRFCNKTGILVKQYLPHYSTKEEWVHHFGSKWKVFQHRKYQFDPRMLLSPGQRIFNNN

>GhCKX87A

MKISSSIFSLISIFILLLISSATSSDFDGFLQCLLQQSNSSLPIIDAILTPNNSTFQSIYQLRANNLRNFLSAISRPVAIITALHPSHAQAAVICAKRHDFQLRIRSGGHDFEGLSYTSKVPFVILDMFNLNSIDIDMSTETAWVQAGATTGELYYRIAEKSNVHGFPSGVWTTLGIGGHFSGDVFWAIRGGGITSFGIILSWRIKLVRVPPRVTVFTVQRTLEQGATELAYRWQQVAPKLPKDLFIRLQPVPINNGGNNNTVRVSFIGHFLGQADGLLRLMNVSFPELGLTRNHCLGMSWVESTVYCANFPNGTSIDMLLDRVQENKVFSKSKSDYYKALIPKQGLETLWQGLMDIEDILVQMNPYGGRMEEILDSETAFAHRAGNLFMVLYRVQWSESHGGINTTERYVEMSRRLYEAMAPYTSSNPREAFLNYRDLDIGSNESDETDFEDAQEYGAKYFRNNFIRLAKAKATIDPENFFKNEQSIPPLPH
